# Phage Therapy in the 21st Century: Is There Modern, Clinical Evidence of Phage-Mediated Efficacy?

**DOI:** 10.3390/ph14111157

**Published:** 2021-11-13

**Authors:** Stephen T. Abedon, Katarzyna M. Danis-Wlodarczyk, Diana R. Alves

**Affiliations:** 1 Department of Microbiology, The Ohio State University, Mansfield, OH 44906, USA; dianinha.alves@gmail.com; 2 Department of Microbial Infection and Immunity, The Ohio State University, Columbus, OH 43210, USA; danis-wlodarczyk.1@osu.edu

**Keywords:** bacteriophage therapy, case study, clinical study, combination therapy, compassionate use, expanded access

## Abstract

Many bacteriophages are obligate killers of bacteria. That this property could be medically useful was first recognized over one hundred years ago, with 2021 being the 100-year anniversary of the first clinical phage therapy publication. Here we consider modern use of phages in clinical settings. Our aim is to answer one question: do phages serve as effective anti-bacterial infection agents when used clinically? An important emphasis of our analyses is on whether phage therapy-associated anti-bacterial infection efficacy can be reasonably distinguished from that associated with often coadministered antibiotics. We find that about half of 70 human phage treatment reports—published in English thus far in the 2000s—are suggestive of phage-mediated anti-bacterial infection efficacy. Two of these are randomized, double-blinded, infection-treatment studies while 14 of those studies, in our opinion, provide superior evidence of a phage role in observed treatment successes. Roughly three-quarters of these potentially phage-mediated outcomes are based on microbiological as well as clinical results, with the rest based on clinical success. Since many of these phage treatments are of infections for which antibiotic therapy had not been successful, their collective effectiveness is suggestive of a valid utility in employing phages to treat otherwise difficult-to-cure bacterial infections.

## 1. Introduction

While earlier studies had hinted at the existence of invisible agents that could negatively impact on bacteria [1,2], discovery of these agents as microorganisms is credited to the French Canadian, Felix d’Hérelle [3,4,5,6]. Subsequently, credit for the discovery of phages was also given to the Englishman, Frederick Twort [7]. Though Twort did not follow up on his finding, d’Hérelle initiated the use of these bacteriophages (or phages) as anti-bacterial infection agents [8,9]. What followed was a blossoming of this technology, led to a significant extent by d’Hérelle himself. The first primary publication documenting clinical phage therapy was not d’Hérelle’s, however, but that of Bruynoghe and Maisin [10]. A number of reviews of human phage therapy discuss this history [2,8,9,11,12,13,14,15].

Notwithstanding its long history, the question persists of whether phage therapy actually works as an anti-bacterial infection strategy, though with the caveat that what “actually works” means is often inconsistent between individual studies, not always straightforward to determine from supplied evidence and, indeed, often is arguable even when ostensibly indisputable. In an important, highly critical review of phage therapy from 1934, Eaton and Bayne-Jones [16,17,18] answered the question, does phage therapy actually work, with a qualified yes. A key line of theirs, amidst much skepticism, stated (p. 1848, emphasis added), “The prevention of recurrence in such conditions as furunculosis and the healing of chronic suppurative conditions of long standing under bacteriophage therapy are *perhaps* the most convincing effects observed”.

Notwithstanding their opinion that phage therapy in some cases could demonstrably contribute to the curing of bacterial infections, the Eaton and Bayne-Jones [16,17,18] report nevertheless contributed, likely along with the widespread introduction of antibiotics in the 1940s, to a substantial decline in the practice of phage therapy by many European, and especially North American practitioners [11,13,19]. This decline did not, however, occur in the former Soviet Union, where phage therapy continued to be researched and developed [2,11,20]. In response to concerns over the diminishing utility of many antibiotics in treating bacterial infections, the Eastern Bloc experience—particularly as centered in former-Soviet Georgia, as well as in Poland [21]—served to inspire a worldwide revival of phage use to treat bacterial infections.

This revival has been ongoing now for approximately 25 years, with an increasing number of clinical studies being published. Here, we focus on clinical efficacy studies published over roughly the last two decades (2000 through approximately the first half of 2021), as no such studies appear to have been published in English during all of the 1990s [14]. Our primary goal in this effort is to address the key question regarding phage treatment of bacterial infections: have phages in clinical studies delivered anti-bacterial infection efficacy in this modern, presumably more medically rigorous era? We summarize our answers to this question in Table 1, which also serves as a guide to the rest of the manuscript. We seek primarily to justify as well as highlight in the main text (Section 3 and Section 4) and Supplementary Materials (Sections S7 and S8) the assertions presented in this table.

We note that these assertions in many cases should be viewed as informed opinions rather than as uncontroversial facts. Consistently, we have not attempted to be shy about our uncertainty despite our otherwise critical analyses of individual studies. Indeed, we not only invite other opinions but feel strongly that the field of phage therapy would benefit from discussing individual studies more critically from a perspective of whether phages have actually contributed to observed treatment successes or, indeed, whether in specific cases treatment successes have been observed at all. Lastly, this opinion piece is not intended to be a review of phage therapy and its implementation, so though we have sought to provide sufficient detail so that an appreciation of phage impact in specific studies can be appreciated, we point the reader to the actual publications for additional information (see Table S1 found in the Supplementary Materials for a summary of many additional treatment details). In the section that follows we provide some context to our analyses as presented in subsequent sections.

### Complicating Factors

Phage treatments generally are thought to be more effective against newer, acute bacterial infections, e.g., when phage treatments are initiated days rather than months after the start of infections or indeed prophylactically [22], and this preference presumably stems from target bacteria not having yet entered into more phage-resistant physical or physiological states (e.g., [23]). In practice, however, published clinical treatments are typically of chronic or persistent bacterial infections [24], and particularly infections which antibiotic treatments have failed to cure [25,26,27,28]. Published clinical phage therapy also has primarily been undertaken under the auspices of compassionate use or treatment (a.k.a. expanded access or, in Australia, the Special Access Scheme [29,30,31,32,33]), rather than in the course of clinical trials. Thus, a typical scenario has infection treatments beginning not with phage treatments but with antibiotic therapy. Only if antibiotic treatments are not successful in resolving an infection might phages then be introduced. The main goal of this approach, however, has been patient recovery rather than testing of phage responsibility for treatment successes.

**Table 1:** Proposed Study Contributions to Evidence of Phage-Mediated Clinical Efficacy ^1,2^.

Phage-Mediated Efficacy Demonstrated?	Publications
Better evidence of phage involvement in observed efficacy (PIS ^3^ = 2; *n* = 14). See Section 3 for narratives	Johri et al., 2021 [34], Bao et al., 2020 [35], Jault et al., 2019 [36], Rogóz et al., 2019 [25], Fish et al., 2018 [37], Jennes et al., 2017 [38], Łusiak-Szelachowska et al., 2017 [39], Fish et al., 2016 [40] ^4^, Międzybrodzki et al., 2012 [41], Letkiewicz et al., 2010 [42], Letkiewicz et al., 2009 [43], Kutateladze and Adamia, 2008 [44], Leszczynski et al., 2006 [45], Weber-Dąbrowska et al., 2003 [46]
Evidence of phage involvement in observed efficacy (PIS = 1; *n* = 21). See Section 4 for narratives	Cano et al., 2021 [47], Wu et al., 2021 [48], Corbellino et al., 2020 [49], Rubalskii et al., 2020 [50], Aslam et al., 2019 [51], Dedrick et al., 2019 [52], Febvre et al., 2019 [53], Kuipers et al., 2019 [54], Maddocks et al., 2019 [55], Nir-Paz et al., 2019 [56], Ooi et al., 2019 [57], Chan et al., 2018 [58], Hoyle et al., 2018 [59], Morozova et al., 2018 [60], Zhvania et al., 2017 [61], Fadlallah et al., 2015 [62], Lecion et al., 2013 [63], Kvachadze et al., 2011 [64], Wright et al., 2009 [65], Jikia et al., 2005 [66], Weber-Dąbrowska et al., 2000 [67]
Insufficient evidence of phage involvement in observed efficacy (PIS = 0; *n* = 35) ^5^. See Section S7 for narratives (Supplementary Materials)	Doub et al., 2021 [68], Ferry et al., 2021 [69], Lebeaux et al., 2021 [70], Leitner et al., 2021 [71], Łusiak-Szelachowska et al., 2021 [72], Ramirez-Sanchez et al., 2021 [73] ^6^, Rostkowska et al., 2021 [74], Tan et al., 2021 [75], Aslam et al., 2020 [76], Doub et al., 2020 [77], Ferry et al., 2020 [78], Ferry et al., 2020 [79], Gainey et al., 2020 [80] ^6^, Petrovic Fabijan et al., 2020 [81], Qin et al., 2020 [82], Aslam et al., 2019 [83], Gupta et al., 2019 [84], Law et al., 2019 [85], Onsea et al., 2019 [86], Tkhilaishvili et al., 2019 [87], Duplessis et al., 2018 [88], Ferry et al., 2018 [89], Ferry et al., 2018 [90], Fish et al., 2018 [91], LaVergne et al., 2018 [92], Patey et al., 2018 [29], Ujmajuridze et al., 2018 [93], Schooley et al., 2017 [94], Kutateladze, 2015 [95], Khawaldeh et al., 2011 [96], Marza et al., 2006 [97], Weber-Dąbrowska et al., 2006 [98], Markoishvili et al., 2002 [99], Weber-Dąbrowska et al., 2001 [100], Weber-Dąbrowska et al., 2000 [101]
Little or no efficacy observed (PIS = NA; *n* = 14). See Section S8 for narratives (Supplementary Materials)	Dedrick et al., 2021 [102], Grubb et al., 2020 [103], Gilbey et al., 2019 [104], Gindin et al., 2019 [105], McCallin et al., 2018 [106], Sarker et al., 2017 [107], Sarker et al., 2016 [108], Łusiak-Szelachowska et al., 2014 [109], Rose et al., 2014 [110], McCallin et al., 2013 [111], Sarker et al., 2012 [112], Rhoads et al., 2009 [113], Bruttin and Brüssow, 2005 [114], Weber-Dąbrowska et al., 2002 [115]

^1^: Summary of etiologies treated: *Acinetobacter baumannii*, *Achromobacter* spp., *Achromobacter xylosoxidans*, *Burkholderia dolosa*, *Citrobacter* spp., *Enterobacter* spp., *Escherichia coli*, *Enterococcus faecium*, *Enterococcus faecalis*, *Enterococcus* spp., *Escherichia* spp., *Enterobacter cloacae*, *Gram negative*, *Klebsiella* spp., *Klebsiella oxytoca*, *Klebsiella pneumoniae*, *Mycobacterium abscessus*, *Morganella morganii*, *Pseudomonas aeruginosa*, *Proteus mirabilis*, *Proteus* spp., *Pseudomonas* spp.; *Staphylococcus aureus*, *Staphylococcus epidermidis*, *Staphylococcus haemolyticus*, *Staphylococcus homis*, *Salmonella* spp., *Serratia marcescens*, *Streptococcus mitis*, *Stenotrophomonas* spp., *Streptococcus* spp., *Staphylococcus simularans*, *Streptococcus agalactiae*, *Staphylococcus saprophyticus*, *Staphylococcus* spp. ^2^: In total, *n* = 84 with 70 publications indicating anti-bacterial infection efficacy and at least 35 indicating evidence of phage contribution to that efficacy. ^3^: PIS = Phage Impact Score, as is also used in Table S1. ^4^:Fish et al., 2016 [40] is discussed under the heading of Fish et al. [37], Section 3.5. ^5^: This row shows studies for which there is evidence of treatment efficacy but, in our opinion, there is insufficient evidence to suggest that this efficacy was phage-mediated. See Supplementary Materials for discussion of those studies. ^6^: These references we suggest should be described as having a PIS of ‘0.5’, that is, somewhere between 0 and 1, though they are discussed in Sections S7.6 and S7.12 (Supplementary Materials), where Section S7 otherwise covers studies for which a PIS designation of 0 is suggested.

Introduction of phages to the treatment of bacterial infections may be (i) in association with a continuation of previous antibiotic treatments, (ii) in association with use of new-to-the-infection antibiotics, (iii) following the discontinuation of antibiotic treatments, or (iv), more rarely, without prior antibiotic treatment. When new antibiotics are introduced during phage treatments, it can be difficult to distinguish antibiotic anti-bacterial infection action from phage anti-bacterial infection action. If antibiotic treatments have not been successful over long periods but are not discontinued prior to the start of phage treatments, then the likelihood that subsequent efficacy is phage-associated, rather than solely antibiotic-associated, should be assumed to be greater. It is still possible, though, that long-continued antibiotic treatments ultimately could have been efficacious, however. Lastly is the case where phages are used alone, particularly after a reasonable delay since antibiotics were last applied, or without prior antibiotic treatment at all. Only in these latter cases can one with reasonably high assurance rule out antibiotics as being responsible for observed anti-bacterial infection activity.

In many cases, it is the temporal relationship between the start of new treatments and observed infection improvement that is especially relevant toward inferring a phage role in mediating that outcome. It is always important to keep in mind, however, that even long-standing bacterial infections can spontaneously improve, though the longer the duration of an infection prior to phage treatment, such as many months or even years, then the higher the likelihood that an infection is refractory to spontaneous improvement. There is also the question of for how long a lack of infection recurrence has been measured following the conclusion of therapies, i.e., so as to confirm treatment success, with months and years more convincing of infection curing than days or weeks. Lastly, there have been suggestions in the literature that phages may not directly supply anti-bacterial infection efficacy but instead that bacterial debris—such as found in minimally purified phage lysates or as generated by phage-induced bacterial lysis in situ—might provide a ‘vaccine’ effect [11,17,18]. Rapid improvements immediately following phage treatments, however, may suggest less of a role of at least active immunity in resulting efficacy, and more of a role of phage virions infecting and then directly killing targeted bacteria. In any case, we are not able to distinguish vaccine effects from more direct phage impacts on bacteria in our analyses.

Our goal here, as based on these various notions, is one of identifying evidence of phage contribution to treatment efficacy as found in the primary clinical phage therapy literature. This may be evidenced especially by rapid treatment success that is both long lasting and unlikely to be a consequence primarily of antibiotic action. Our goal, however, is not to criticize treatments when such ‘proof’ does not appear to be present. In many cases it could very well be that phages are providing a positive and even overwhelmingly significant contribution to treatment success, but this is not the same as those studies being convincing of phage-mediated anti-bacterial infection activity in a clinical setting. For completeness, we reviewed all recent English-language clinical phage therapy cases that we could identify, and in some cases, there is indeed, in our opinion, evidence of phage-associated efficacy, which we document (as found particularly in Section 3 and Section 4, below.)

We are intentionally cautious in bestowing claims of evidence of phage-mediated treatment efficacy. Nonetheless, we are not demanding either randomized double blinding or use of negative-treatment controls before claiming evidence of phage-mediated efficacy. Instead, we suggest that there is value in considering what information case studies may be providing to us about the impact of phage therapy on bacterial infections. We present narratives of these studies per section in a descending-year, alphabetical order, with headings indicating authors, date of publication, the etiology or etiologies targeted, and the nature of the bacterial infection targeted (species names of targeted etiologies are spelled out in full in a footnote to Table 1). The primary goal of these narratives, as noted, is to justify which category a study has been placed in within Table 1. Studies that in our opinion provide evidence of phage-mediated efficacy, we discuss in Section 3 and Section 4, with those supplying superior evidence presented in Section 3. Alternatively, studies that in our opinion supply insufficient evidence of phage-mediated efficacy we present in Section S7 (Supplementary Materials). Many 2018 and earlier studies have been described elsewhere by one of us [14,24,116,117], so are presented here as narratives in less detail.

## 2. Methods

The phage therapy literature is extremely diverse in terms of types of infections treated, bacterial etiologies involved, phages employed, phage titers used, phage delivery routes, cotreatments, and clinical measures of outcomes. As a consequence, no single metric exists which, to our knowledge, allows for uniform assessment of treatment success, particularly short of demonstration of the elimination of targeted bacteria along with substantial clinical improvement that persists for long periods, e.g., such as one year. Our approach here, therefore, is to assess, through careful reading, individual studies on their own, individual merits.

Toward that end, we provide narratives of each study (Section 3 and Section 4, Sections S7 and S8, the latter two found in the Supplementary Materials). Our primary goals in doing so, however, is solely to indicate what information exists that can be used to answer two questions. These are, (i) was anti-bacterial infection activity associated with treatments? and (ii) is evidence provided which can attribute some amount of that activity to the use of phages? In other words, what evidence exists that had a treatment not included phages, positive treatment outcomes would have differed? Importantly, we do not rely solely on the conclusions provided by authors in making these assessments.

What is not being attempted is to advocate one treatment approach over another, to compare the usefulness of different approaches to the phage therapy of different infection types, to assess the use of different phage types, or to determine the effectiveness of different phage-antibiotic combinations. In addition, we are not attempting to assess superiority of phage therapies over comparable standard-of-care treatments. That is, our concern here is not in how phage therapies should be used in clinical medicine but whether there is clinical evidence to justify further testing of phage therapies clinically. Thus, in particular, is the medical use of phages as anti-bacterial infection agents justified based on available clinical evidence? We also suggest how publications may be improved toward better answering that question.

Note that a listing of phage-related clinical trials can be found via the website, ClinicalTrials.gov. A total of 25 potential phage-therapy efficacy trials were identified. Of these, one was withdrawn, two were terminated [108], three have a status listed as “Unknown” [36,41], five are not yet recruiting, four are recruiting, one is active, one is “Available”, six are indicated as having been completed [53,71], and two are listed as “No longer available”. All five trials for which publications reporting trial results have been listed are reviewed here. These are Międzybrodzki et al. [41] (Section 3.9), Febvre et al. [53] (Section 4.7), Sarker et al. [108] (Section S8.7, Supplementary Materials), Jault et al. [36] (Section 3.3), and Leitner et al. [71] (Section S7.4, Supplementary Materials). An additional efficacy clinical trial that is not listed on ClinicalTrials.gov but is reviewed here is that of Wright et al. [65] (Section 4.19).

## 3. Better Evidence of Clinical Phage-Mediated Efficacy

In this section, we provide summaries of clinical phage-therapy studies that we have categorized in Table 1 as having a Phage Impact Score (PIS) of 2. Our primary aim in this section is to justify our inclusion of these studies in that category.

### 3.1. Johri et al., 2021, Various Etiologies, Chronic Prostatitis

Johri et al. [34] treated a 33-year-old male who was diagnosed with chronic bacterial prostatitis following digital rectal examination, though no bacteria were initially cultured from his urine and antibiotic treatments were not effective. Subsequently, prior to the initiation of phage therapy at the Eliava Phage Therapy Center, in Tbilisi, Georgia, bacteria were cultured and these included *E. faecalis*, *S. aureus*, *S. epidermidis*, and *S. haemolyticus*. Subsequently, *S. mitis* was cultured as well, though *S. aureus* was absent at that time. Treatment involved multiple phages and routes: so-called Intesti-phage and Fersis-phage cocktails along with “Staphylococcal phage”, and oral dosing as well as rectal and urethral delivery. Initially this was over an at least three-month period with symptomatic improvement. Over subsequent months, phages targeting *S. mitis* were added to the treatment, with further symptomatic improvement. Ultimately, cultures were negative of pathogenic bacteria. The failure of antibiotics during initial treatments and lack of indication of continued antibiotic treatments in association with the phage therapy, both in combination with the apparently symptomatic and microbiological success of the subsequent phage treatments, is highly suggestive of phage-associated antibacterial and anti-bacterial infection efficacy.

### 3.2. Bao et al., 2020, K. pneumoniae, Urinary Tract

Bao et al. [35] treated a 63-year-old female for a *K. pneumonia* infection of her urinary tract and this was following unsuccessful treatment with various antibiotics. This was done serially, via bladder instillation, and employing three different phage cocktails, all derived from an in-house phage collection. During the first round of phage treatment, lasting 5 days, resistance developed to the initial five-phage cocktail (consisting of phages SZ-1, SZ-2, SZ-3, SZ-6, and SZ-8). Treatment using a second, five-phage cocktail (consisting of phages Kp158, Kp165, Kp166, Kp167, and Kp169) to which the strain resistant to the first cocktail was sensitive, was then attempted. Again, resistance to the cocktail developed. The infection was then treated with a third, now six-phage cocktail (Kp152, Kp154, Kp155, Kp164, Kp6377, and HD001), and this was done in combination with sulfamethoxazole-trimethoprim.

Though without phages present the etiology was found to be resistant to sulfamethoxazole-trimethoprim in vitro, in the presence of these antibiotics phage resistance failed to develop, also in vitro. Following another 5 days of treatment with this third cocktail and sulfamethoxazole-trimethoprim, the bacterium was eliminated, and no symptoms were present for at least 6 months. Given that previous treatment with sulfamethoxazole had been unsuccessful in resolving the infection and the infecting bacterium was found to be resistant to sulfamethoxazole-trimethoprim in vitro, along with development of resistance by the etiology to the first two phage cocktails, it seems likely that the infection responded to treatment with the third phage cocktail. Furthermore, phage-resistant mutants may have become susceptible to sulfamethoxazole-trimethoprim, perhaps similarly to that seen by Chan et al. [58], as discussed in Section 4.12. Thus, phage-mediated anti-bacterial infection efficacy appears to have been demonstrated in this case study.

### 3.3. Jault et al., 2019, P. aeruginosa, Burn Wound

Jault et al. [36] present a double-blinded, randomized, phase I/II phage-treatment clinical trial of burn wounds taking place in Belgium and France with *P. aeruginosa* as the target etiology. A topically applied cocktail consisting of 12 phages was used (called PP1131) with a titer estimated at only 10 to 100 PFUs/mL rather than the intended 10^6^ PFUs/mL. Efficacy was determined by (p. 39) “the time taken for a sustained reduction in bacterial burden of two quadrants or more assessed by semiquantitative culture results”. Sustained reductions were observed with phage treatments alone, though there was less efficacy relative to the standard-of-care sulfadiazine silver emulsion cream treatment. For two patients of a total of 12 or of 13 depending on the treatment group (phage vs. standard-of-care sulfadiazine silver), antibiotic treatment was used as well. Overall, the results of this study are suggestive of phage-mediated anti-bacterial infection efficacy—even if inferior to standard-of-care treatment—and this effectiveness was seen despite issues of excessive treatment phage instability during storage prior to treatment.

### 3.4. Rogóz et al., 2019, S. aureus, Orthopedic

Rogóz et al. [25] document a number of orthopedic, mostly *S. aureus* infections treated by the Phage Therapy Unit of the Hirszfeld Institute in Wrocław, Poland, with two cases discussed in detail. These are treatments (p. 199) of “patients suffering from chronic bacterial infections where antibiotics have failed or are contraindicated.” Phage dosing was oral and/or topical, the latter “via fistular irrigation and/or wet compresses on the external orifice of the fistula” (p. 199). The breakdown of results for 76 patients treated for orthopedic, mostly implant-associated infections were 11 (14.5%, but shown as “1” in the publication) showing “pathogen eradication and/or recovery”, 5 (6.6%) showing “good clinical result”, 17 (22.4%) showing “clinical improvement”, 9 (11.8%) showing “questionable clinical improvement”, 13 (17.1%) showing “transient clinical improvement”, 18 (23.7%) showing “no response to treatment”, and 3 (3.9%) showing “clinical deterioration”. Together, the top three categories add up to a 43.4% success rate, which is consistent with the success documented in a previous study from the same institute [41]. Indeed, a number of cases reported by Rogóż et al. [25] appear to have been from the latter publication.

Importantly, most of the successes, 24 of 33, did not involve antibiotic treatment during phage therapy, including seven of the eleven cases resulting in “pathogen eradication and/or recovery”. We therefore view this study as indicative of demonstrating phage-mediated anti-bacterial infection effectiveness. Another group of patients treated for other infection types is reported, with a top-three-categories success rate of 3.4%, though it is not as clear that antibiotics were not used in conjunction with those phage treatments.

### 3.5. Fish et al., 2018, S. aureus, Diabetic Toe Ulcers

Fish et al. [37] is a methods-type study that discusses two additional cases from those discussed in Fish et al. [40]. For the sake of documentation, we consider these publications to constitute a single study, though in Table 1 and Table S1 we list them separately. Fish et al. [40] treated a series of patients with *S. aureus*-infected diabetic toe ulcers using a single phage isolate. Treatments consisted of compassionate use and employed a single phage type (Sb-1) that is commercially available in Georgia, with a titer ranging from 10^7^ to 10^8^ PFU/mL. Toes were deemed from experience by the physician (Fish) not to be salvageable, with amputation indicated. Antibiotic cotreatments were not used, given patient comorbidities. Phages were applied topically once per week, with numerous examples of successful healing presented. We feel that these case reports provide strong evidence for phage-mediated anti-bacterial infection efficacy. See Abedon [14,24,117] for further discussion of these cases.

### 3.6. Jennes et al., 2017, P. aeruginosa, Septicemia

Jennes et al. [38] treated a 61-year-old male with phages for a septicemic *P. aeruginosa* infection, via compassionate use. Treatment with a two-phage cocktail (BFC1) was intravenous (once daily) and topical to wounds (50 mL, 3 times daily), for a total of 10 concurrent days each. This was preceded by 3 weeks with a 2-week treatment with colistin, ending 1 week prior to the start of phage treatment. As the authors note, following implementation of phage treatments, as corroborated in their Figure 2 (p. 2), “Immediately, blood cultures turned negative, [C-reactive protein] levels dropped and the fever disappeared. Kidney function recovered after a few days.” Pressure sore infection with *P. aeruginosa* persisted, however, and the patient died 4 months later of an unrelated (*K. pneumoniae*) septicemia. Though the colistin treatment resulted in reductions in body temperature (from 39 °C to 37 °C), the patient’s temperature returned to 39 °C less than 1 week after colistin treatment was discontinued, but then dropped down to 37 °C again after phage therapy was initiated. Overall, given the timing of improvement following the start of phage therapy, as well as the gap between colistin treatment and phage therapy, it seems likely that treatment success here can be attributed to the phage treatment.

### 3.7. Łusiak-Szelachowska et al., 2017, Various Etiologies, Various Infection Types

Łusiak-Szelachowska et al. [39] explored the impact of serum antibodies on phage therapy efficacy conducted under the auspices of compassionate use. Though not explicitly indicated in the study, these generally are patients for which conventional antibacterial treatments were not previously found to be effective [41]. In total, 62 adults subject to phage therapy were examined. Phages were applied intrarectally, locally, and/or per os, and without coadministered antibiotics. A good efficacy response was seen in ~40% of patients where “Good” was defined as (p. 115), “…almost complete subsidence of the infection symptoms confirmed by the results of laboratory assays, together with a significant improvement of the patient’s condition…” As these results were achieved without antibiotic cotreatment, and were of infections that had a history of absence of recovery, they are indicative of phage-mediated anti-bacterial infection efficacy.

### 3.8. Fish et al., 2016, S. aureus-Infected Diabetic Toe Ulcers

Fish et al. [37] is a continuation of the Fish et al. [40] study, with the latter discussed there (Section 3.5). See also Fish et al. [91] (Section S7, Supplementary Materials).

### 3.9. Międzybrodzki et al., 2012, Various Etiologies, Various Infection Types

Międzybrodzki et al. [41] present the results of phage treatment of 153 patients for various pathogens and diseases, as taking place within a compassionate use context in the Phage Therapy Unit of the Hirszfeld Institute in Wrocław, Poland. Treatment especially was of infections that had not previously responded sufficiently well to antibiotics. Overall, phage treatments were associated with better results for some categories of infections, administration routes, or targeted pathogens vs. others.

Elimination of target bacteria was seen in 18.3% of cases. Clinical results that were described as good were seen in 8.5% of cases. Clinical improvement was seen in 13.1% of cases. This is a total of about 40% of cases displaying some improvement. Antibiotic and other medicament antibacterial treatments were not discontinued prior to phage treatment for 28.8% of cases. By their Table IV, however, “pathogen eradication and/or recovery” was seen in 22 patients of [109] for which medicaments were not used, a “good clinical result” but not eradication was seen for seven patients with phage use alone, and “clinical improvement” was seen with 15 patients also without comedicament treatment. Additional discussion of this article is provided in Abedon [24,117].

### 3.10. Letkiewicz et al., 2009, 2010, E. faecalis, Chronic Prostatitis

Letkiewicz et al. [43] present the compassionate-use treatment with phages of three patients with chronic prostatitis, treating *E. faecalis*. This was done at the Phage Therapy Unit of the Hirszfeld Institute in Wrocław, Poland. Previous antibiotic treatments were unsuccessful, with no antibiotic treatment occurring over the month prior to phage therapy. Twice daily, 10 mL of phages with titers in the range of 10^8^ PFU/mL were delivered rectally. The duration of treatments ranged from 28 to 33 days. Prostate fluid was found to be negative for *E. faecalis* for the three different patients at weeks 1 and 8, 8 and 25, and 3 and 10 after phage therapy, but not prior to the start of phage treatment. Following phage treatment, for all three cases, prostate volumes also declined, prostate consistency improved, and urinary flow rate increased. These results all point to a role of phages in the resulting treatment efficacy. Letkiewicz et al. [42] describe the treatment of an additional 22 patients for various pathogens and using various routes of delivery, with pathogen eradication seen in at least half of the cases. Both studies are also discussed in Abedon [24].

### 3.11. Kutateladze and Adamia, 2008, Various Etiologies, Various Infection Types

Kutateladze and Adamia [44] present a compilation of phage therapy results obtained by the Eliava Institute, Tbilisi, Georgia. A key element of the reporting is whether phages were used in combination with antibiotics, with antibiotics only, or untreated controls included in the studies. One example is the following (p. 428): “…results of treatment of staphylococcal sepsis… From the group treated only by phage, 41.3% of the patients recovered totally, 13.1% of the patients from this group had improvement, 45.6% of the patients had no effects.” From this list of results, we conclude that therapy success was probably a consequence of phage action. Further (pp. 428–429), “From the group treated simultaneously by phages and antibiotics, 77.5% recovered totally, 10% had improvements, 12.5% with no effect. From the control group, 11.1% recovered totally, 11.1% had improvement, 77.8% no effect.” We include only the above-quoted results from this publication in our Table 1. This publication was partially summarized in Abedon [14].

### 3.12. Leszczynski et al., 2006, S. aureus, Gut Decolonization

Leszczynski et al. [45] describe the elimination of *S. aureus* from the gastrointestinal tract of a 30-year-old nurse. This was done to limit the potential for future urinary tract infections in this patient, who previously had been successfully treated using antibiotics. Phages were used because of a desire by the patient not to be treated with antibiotics to effect *S. aureus* decolonization. Treatment consisted of application of a three-phage cocktail (phages 676/F, A3/R, and A5/80) via the oral route, three times a day for 4 weeks, and resulted in an absence of the *S. aureus* strain (methicillin resistant) in faeces (determined via rectal swabs) for at least six months. The absence of cotreatment with antibiotics is strongly suggestive that the decolonization was accomplished through phage action alone. This study is briefly discussed in Abedon [14].

### 3.13. Weber-Dąbrowska et al., 2003, Various Etiologies, Sepsis

Weber-Dąbrowska et al. [46] describe the treatment with phages of 94 patients with sepsis caused by various bacterial pathogens, including 33 monoinfections and 61 mixed infections. In all cases, antibiotic treatments were tried prior to phage treatments and were not successful. In all but 23 cases, antibiotic treatment was continued along with phage therapy. Phages were supplied orally, thrice daily, with prior gastric acid neutralization and, we presume, were supplied from an in-house collection. Complete recovery was indicated for 85% of cases, with no statistical difference in results between, with and without associated antibiotic treatment. This result we feel is consistent with significant phage contribution to treatment success, particularly without antibiotic cotreatment but also with it.

## 4. Evidence of Clinical Phage-Mediated Efficacy

In this section, we provide summaries of clinical phage-therapy studies that we categorized in Table 1 as having a Phage Impact Score (PIS) of 1. Equivalent to the summaries provided in Section 3, our primary aim in this section is to justify our inclusion of these studies in that category, which in this case indicates that evidence of phage-mediated efficacy was presented.

### 4.1. Cano et al., 2021, K. pneumoniae, Prosthetic Joint

Cano et al. [47] describe the treatment of a 62-year-old male with a prosthetic knee infected with *K. pneumoniae*. Following multiple infections, antibiotic treatments, and surgeries, intravenous phage therapy via expanded-access was undertaken as an alternative to amputation. Phage KpJH46Φ2 was isolated for this case and produced “clear plaques of good size” on the *K. pneumoniae* strain isolated from the patient. The resulting formulation (22 endotoxin units/mL) was applied a total of 40 times in 50 mL aliquots, each containing 6.3 × 10^10^ plaque-forming units (PFU)/mL. This was administered once daily over 8 weeks. The antibiotic, minocycline, was dosed along with the phages, starting prior to the beginning of phage therapy by roughly 3 months. Success was measured as 34 weeks without symptoms of infection following the end of phage treatment, with minocycline treatment nevertheless continued for the sake of prevention of relapse.

For this study, it is possible that minocycline alone was responsible for the patient’s recovery, though there would have been a multiple month lag between the start of minocycline treatment and observation of recovery. Unfortunately, no timeline is reported for improvements following the start of phage treatment, other than that improvement in symptoms did start after the initiation of phage treatment. Overall, this study seems to point to an efficacy role for phages, though the possibility could still exist that treatment success instead had been a consequence of a delayed impact of minocycline. An important argument against that conclusion, however, is that a number of proinflammatory cytokines declined in concentration following the start of phage therapy from a baseline measured prior to the start of phage therapy. Thus, it appears reasonably likely that phages were responsible for the treatment success.

### 4.2. Wu et al., 2021, A. baumannii, Lung

Wu et al. [48] employed phage therapy to treat secondary *A. baumannii* (carbapenem-resistant, i.e., CRAB) pulmonary infections. These were in four COVID-19 patients within a context of compassionate use. A single phage (ΦAb124) or a two-phage cocktail was used that had been dialyzed against and then diluted into saline, with treatments consisting of two nebulized 10 mL doses of 10^8^ PFU/mL provided one-hour apart. The phages were chosen based on producing large, clear plaques (phage ΦAb124) and an ability to infect a phage ΦAb124-resisant *A. baumannii* mutant, also producing clear plaques (phage ΦAb121).

Treatment of patient 1 with just the single phage resulted in (p. 615) “A decline in semi-quantitative CRAB burden in suctioned sputum…” This, however, was followed by a rise (“a resurgence was soon emerged”) in phage-resistant isolates. Following treatment of patient 1, the next three patients were treated with the phage cocktail. Microbiologically, a similar time course as with the first patient ensued. In terms of clinical status scores, patient 1 improved substantially after 14 days, patient 2 started improving within the first week after treatment, patient 4 did not appreciably improve and died of respiratory failure one month later, and patient 3 died after 10 days, though presumptively of a *K. pneumoniae* infection, as their *A. baumannii* infection was reported as eliminated. Patient 1 also experienced a cytokine storm and transient fever starting 4 h after phage treatment.

In our opinion, the elimination of *A. baumannii* from patient 3 represents a reasonably good indication that phage therapy may have been effective in that case. Patient 2 also displayed an additional, nonpulmonary infection, also with *A. baumannii*, that was treated separately with the phage cocktail, resulting in elimination of this other infection. In their semiquantitative assays, *A. baumannii* burden in the lungs of all patients was found to have decreased as measured 24 h after treatment. Thus, Wu et al. [48] seem to supply evidence of an association between phage treatment and reductions in bacterial loads as well as rise of phage-resistant bacterial strains. In addition, they note that (p. 617) “conventional antibiotic treatment had been tried previously and failed to suppress [*A. baumannii*] infection or improve the condition of these patients.” Thus, we categorize these results as being sufficiently promising of phage-mediated efficacy to deserve follow up.

### 4.3. Corbellino et al., 2020, K. pneumoniae, Gut Decolonization

Corbellino et al. [49] reported on the phage treatment of a 57-year-old female infected at several sites with *K. pneumoniae*. Phage vB_KpnM_GF was applied orally (10 mL of 10^6^ PFU/mL, twice daily for 3 weeks) and intrarectally (10^6^ PFU daily over the first two weeks of treatment). This was on an out-patient basis beginning a little less than six months after initial detection of the infection. This phage treatment resulted, after 15 days of dosing, in an inability to culture the bacterium from the patient or to detect associated carbapenemase genes molecularly. Since bacterial elimination potentially could have been initiated prior to clearance of ceftazidime-avibactam from the patient, the authors did not want to claim that the antibiotic treatment did not play a role in treatment success nor that the bacterium could not have spontaneously decolonized. Nonetheless, it is our opinion that this study is supportive of the phage treatment being responsible for the reported anti-bacterial infection efficacy. Note as an aside, however, that though the authors suggest that this study could be the first publication reporting phage-mediated gut decolonization to date, at least in the modern era, there is, in fact, an earlier, relatively recent report of gut decolonization of methicillin-resistant *S. aureus* [45] (Section 3.12).

### 4.4. Rubalskii et al., 2020, Various Etiologies, Cardiothoracic Surgery

Rubalskii et al. [50] treated eight patients (seven males and one female with ages ranging from 13 to 66) for a variety of infections caused by a variety of bacterial species (*E. coli*, *E. faecium*, *K. pneumoniae*, *P. aeruginosa*, or *S. aureus*), with the commonality being their association with cardiothoracic surgery outcomes (implants or transplants). All cases were initiated following failure of standard antibiotic treatments to eradicate infecting bacteria, and three cases involved phage application in association with “Fibrin glue”, which is a means of phage immobilization and sustained release involving fibrinogen and thrombin. Either single phages or phage cocktails were used, depending on the case, with either single or multiple dosings, and either one or two routes of phage application per patient. Treatment success was reported in all but one case, as determined microbiologically, though antibiotic treatments that were in place prior to the start of phage treatment were continued throughout phage treatment. It is possible that prior infection resistance to these antibiotics was lost coinciding with phage treatment. It is unlikely, however, that this was true over the entire case series. Therefore, we feel that this study supplies evidence of phage therapy anti-bacterial infection efficacy.

### 4.5. Aslam et al., 2019, S. aureus, Localized and Disseminated

Aslam et al. [51] treated a 65-year-old male for an *S. aureus* infection that was associated with an implanted device as well as osteomyelitis and bacteremia. A three-phage cocktail was supplied by AmpliPhi (AB-SA01) and delivered intravenously as 3 × 10^9^ PFU twice daily for 4 weeks. Following previous debridements (the last occurring 8 weeks prior to the start of phage treatment) and “prolonged” antibiotic treatment (starting ~2.5 years prior to the start of phage treatment), phage therapy was initiated in association with continued treatments with cefazolin (started ~8 weeks prior to phage treatment) and minocycline (started ~4 weeks prior to phage treatment).

Just before the start of phage therapy (2 days prior), the associated sternal wound was still *S. aureus*-positive whereas 5 days after the start of phage therapy the same site was found to be *S. aureus*-negative. Two weeks later, the wound was again *S. aureus*-positive as well as *S. epidermidis*-positive, but returned to negative for both the following week, without any change in treatment approach. During subsequent heart transplantation, “scant” *S. aureus* was noted in two locations internally. No isolates had been found to have become either phage resistant or changed in their antibiotic susceptibility, and all *S. aureus* was found to have been eliminated upon eight further weeks of cefazolin treatment. We are of the opinion that this study demonstrated phage-mediated anti-bacterial infection efficacy, particularly given the timing of declines in bacterial counts soon after the start of phage treatment, while declines in bacterial counts were not previously observed despite multiple weeks of treatment with cefazolin and minocycline. It appears, nevertheless, that antibiotic treatment may have played a role, perhaps particularly following phage-mediated disruption of *S. aureus* infection foci.

### 4.6. Dedrick et al., 2019, M. abscessus, Disseminated Infection

Dedrick et al. [52] treated a 15-year-old cystic-fibrosis, lung-transplant recipient for a disseminated *M. abscessus* infection using a three-phage cocktail (BPs, Muddy, and ZoeJ). Phages were derived from the SEA-PHAGES program [118], with BPs and ZoeJ modified using combinations of recombinant genetics and selection. Phages were supplied predominantly intravenously, twice daily for 32 weeks with a dose of 3 × 10^9^ PFU. Resulting serum titers were measured at over 10^9^ PFU/mL early in treatment. The antibiotics amikacin, bedaquiline, clofazimine, imipenem-cilastatin, and tigecycline were used concurrent to phage treatment, but all had been in continuous use in the patient for over 150 days prior to the start of phage therapy. Side effects following phage application were minor and did not include fever.

An initial topical dose was associated with skin lesion improvement, and this prompted further topical application to skin lesions starting one month after the initiation of treatment. The patient improved further over the course of six months, which was up until the time of the writing of the communication. From p. 733, “*M. abscessus* was not isolated from serum or sputum at any point after initiation of phage treatment, although *M. abscessus* was cultured from swabs of slowly resolving skin nodules at 1, 3, 4, and 5 months.”

The authors note, on p. 733, that “Phage treatment was associated with clinical improvement, although we cannot exclude the possibility that patient gains would have occurred without phage treatment. However, we note that patients with similar clinical conditions typically have high morbidity and mortality, that improvement was not temporally associated with cessation or initiation of other drug administrations, and we show evidence to support in vivo phage replication.” Due to the lack of sufficient antibiotic effectiveness for many months prior to the start of phage therapy, we feel that this case report provides evidence for phage-mediated anti-bacterial infection efficacy.

### 4.7. Febvre et al., 2019, E. coli, Gastrointestinal Tract Health Trial

Febvre et al. [53] tested the impact of oral dosing of a four-phage cocktail (PreforPro, which is commercially available as “Perfect flora”, e.g., such as via Amazon.com) on gastrointestinal tract health as well as targeted gut *E. coli*. Antibiotic use within the previous 2 months served as an exclusion criterion for the study. Daily dosing involved consumption of a single capsule, though with no indication of whether the capsule supplied protection from gastric juices. The results from a total of 36 individuals, each serving as their own placebo-treatment control, either 4 weeks of placebo then ≥2 weeks of washout then 4 weeks of phage treatment, or vice versa, were analyzed over the course of this randomized, double-blinded study. The phage cocktail consisted of *E. coli* phages LH01, LL5, LL12, and T4D, provided as 10^6^ PFU.

In addition to differences such as in levels of certain gut bacterial taxa, levels of stool *E. coli* were determined in terms of numbers of amplicon sequence variants. By that criterion, numbers of *E. coli* were reduced on average by about 40% with phage treatment and not significantly with placebo treatment. Though this result seems clearly indicative of phage-mediated reductions in densities of target bacteria, a 40% reduction is not a substantial decrease in numbers and certainly not eradication, and authors noted that the results were associated (p. 6) with “a great deal of variability between the participants”. Nonetheless, the inclusion of a placebo control as well as double blinding makes this experimental result relevant as an indication of phage treatment impact on numbers of target bacteria.

### 4.8. Kuipers et al., 2019, K. pneumoniae, Urinary Tract

Kuipers et al. [54] describe a 58-year-old male recipient of a transplanted kidney with a recurring *K. pneumoniae* urinary tract infection. The etiology was susceptible to meropenem but previous treatments (seven) with meropenem alone were insufficient to prevent recurrence of the infection. Compassionate-use treatment with a combination of meropenem and phages, however, was successful, with cultures remaining negative 14-months after treatment. Phages, supplied by the Eliava Institute in Tbilisi, Georgia, were taken orally in combination with bladder irrigation over a 12-week period, initially twice daily and ultimately every day orally and every other day for the bladder irrigation. The failure of meropenem treatments to prevent recurrence until phages were also used, in combination with the resulting long remission, is suggestive of an important phage anti-bacterial infection contribution to the resolution of this case.

### 4.9. Maddocks et al., 2019, P. aeruginosa, Lung

Maddocks et al. [55] treated a 77-year-old female via Special Access Scheme with a four-phage cocktail supplied by AmpliPhi (AB-PA01) and produced using good manufacturing practices. The phages were delivered twice daily for 1 week, both intravenously (4 × 10^9^ PFU/dose) and via nebulization (1.6 × 10^10^ PFU/dose). This was done to treat a variety of lung-associated infections, i.e., from p. 1180: “…of extensive, necrotizing, pulmonary pseudomonal infection.” Ciprofloxacin and gentamycin treatment were initiated 6 days prior to the start of phage treatment and C-reactive protein declined substantially at the same time, leveling off coincident with the start of phage treatment. Treatment with both was stopped 4 days after the start of phage therapy when resistance to these antibiotics was detected, though this also appears to be the last day that *P. aeruginosa* was detected, from sputum. Further, on the last day of phage treatment, ceftolozane/tazobactam treatment was started. It is possible given this time course that a majority of the bacterial burden was reduced by ciprofloxacin and gentamycin treatment, with remaining bacteria resistant to these antibiotics removed by the phage treatment. We are encouraged in this conclusion especially by the relatively high titers of phages supplied via nebulization, which should have been sufficient—assuming both good penetration of the aerosol and sufficient survival of the phages—to adsorb and thereby eliminate remaining possibly relatively low concentrations of bacteria.

### 4.10. Nir-Paz et al., 2019, A. baumannii and K. pneumoniae, Osteomyelitis

Nir-Paz et al. [56] treated an *A. baumannii*-*K. pneumoniae* mixed-infection osteomyelitis of a 42-year-old male. Phages ΦAbKT21phi3 (5 endotoxin units/mL) and ΦKpKT21phi1 (35 endotoxin units/mL) were used, respectively, in combination with colistin and meropenem. Prior to phage therapy, the patient was treated also with colistin and meropenem as well as with piperacillin-tazobactam, but the infection recurred. Above-the-knee amputation was recommended. Phages were administered intravenously with a total of 5 × 10^7^ PFU of each phage type supplied per treatment. Treatments were over 5 days, then one week without phage dosing, then 6 days with.

Healing was noted (p. 2016) “within a few days after the initiation of phage therapy.” The wound subsequently closed, and the infection did not recur over the following 8 months. Cultures taken at 8 months were negative for the targeted bacteria, though the authors note that (p. 2018) “it is too early to know whether the infection has resolved completely.” Though numbers of phages supplied in this case were fairly low for intravenous delivery [22], the infections targeted were localized. Therefore, it is certainly possible that phage replication in association with infecting bacteria could have made up for this delivered phage-titer deficit [119]. Furthermore, the same antibiotics had been used previously to treat this patient but with infection recurrence. It is possible that the same antibiotics just happened to be effective when they were used at the same time that phages were applied. Nevertheless, we feel that this study is suggestive that the phages contributed to the apparent infection elimination.

### 4.11. Ooi et al., 2019, S. aureus, Chronic Rhinosinusitis

Ooi et al. [57] describe an open label phase I trial taking place in Australia treating chronic rhinosinusitis associated with *S. aureus* colonization in nine patients ranging in age from 18 to 70 years old. The patients had previously undergone endoscopic sinus surgery and (p. 724) “received routine twice-daily saline irrigations before study entry.” If taking intranasal corticosteroids, they were instructed to keep doing so. Oral use of antibiotics was an exclusion criterion, however. A three-phage cocktail (AB-SA01, supplied by AmpliPhi and produced using good manufacturing practices) was applied 2 times per day via intranasal irrigation in doses of 3 × 10^8^ PFU for 7 days, 3 × 10^8^ PFU for 14 days, or 3 × 10^9^ PFU also for 14 days. Unfortunately, the authors note that (p. 728) “no clinically meaningful changes occurred in the validated symptoms scores relevant to sinus disease”, but also that “clinical improvements seen endoscopically may be explained by a reduction in bacterial load and the suspected anti-inflammatory effects of phages.” Elimination of targeted bacteria, however, was seen with two patients based on a semiquantitative analysis. Indeed, results of these latter analyses were from heavy down to negative (bacterial load prior to and following treatment, respectively; 1 patient), from heavy down to light (2 patients), from heavy down to moderate (2 patients), from moderate down to light (3 patients), and from light down to negative (1 patient). These results in combination with patient histories, including prior, routine saline irrigations, are suggestive of phage-mediated reductions in bacterial counts.

### 4.12. Chan et al., 2018, P. aeruginosa, Aortic Graft

Chan et al. [58] present a unique case of expanded access phage therapy of a *P. aeruginosa* aortic graft infection. A 76-year-old male was treated with a phage (OMKO1) that uses the bacterial efflux pump as its adsorption receptor. As a consequence of this, bacterial mutants, which evolved phage resistance by eliminating display of this pump, became antibiotic sensitive to ceftazidime and ciprofloxacin. Treatment with ceftazidime along with ciprofloxacin had been successful in suppressing the infection previously, but not in curing it. Ceftazidime treatment in combination with phage OMKO1—the latter involving just a single direct application to the graft with 10 mL of 10^7^ PFU/mL and 12.5 endotoxin units/mL—resulted in no sign of infection after 4 weeks, nor subsequent sign of recurrence.

An issue with this study is that the duration over which no infection recurrence was seen was relatively short, at least as explicitly described. What is also unclear is whether the *P. aeruginosa* being treated with both the phage and ceftazidime was or was not sensitive to ceftazidime at the start of treatments, particularly given the long history of prior ceftazidime treatment of the patient. In vitro tests with biofilms, however, indicated an ability of treated bacteria to display at least tolerance to ceftazidime. Thus, it is possible that a majority of bacteria were eliminated by the action of ceftazidime, with remaining ceftazidime-resistant bacteria then eliminated by the phage, or instead that a majority of bacteria were eliminated by phage action, with remaining phage-resistant but now ceftazidime-sensitive bacteria removed by the antibiotic. In either case, if it is true that the infection truly was cured by this combined treatment, then as previous ceftazidime treatment had not been similarly successful, it is reasonably likely that phage treatment played at least some role in eliminating at the least the phage-sensitive bacteria.

### 4.13. Hoyle et al., 2018, A. xylosoxidans, Lung with Cystic Fibrosis

Hoyle et al. [59] treated a 17-year-old female with cystic fibrosis afflicted with an *A. xylosoxidans* lung infection. Rounds of treatment were for 20 days using a two-phage cocktail supplied by the Eliava Institute (Tbilisi, Georgia). These phages were delivered daily via nebulizer (6 × 10^8^ PFU in ~5 mL) as well as orally twice daily. This regimen was repeated four times over the course of 1 year. The patient displayed improved symptoms and was subsequently subject to additional antibiotic as well as phage treatment, which resulted in further improvement in symptoms. Previous antibiotic treatments had not been sufficiently effective, thereby leading to the use of phages, and antibiotic treatment was discontinued prior to introduction of phages (N. Hoyle, personal communication). Treatment took place at the Eliava Phage Therapy Center, also in Tbilisi, Georgia. Given that antibiotic treatment was discontinued prior to the start of phage treatment, it appears that observed clinical improvement can be attributed to the phage treatment.

### 4.14. Morozova et al., 2018, S. aureus, Diabetic Toe Ulcers

Morozova et al. [60] is a methods paper that describes the phage treatment of diabetic toe ulcers infected with *S. aureus*. Although it is not stated explicitly that antibiotics were not used as cotreatments, the authors note for one of the two cases (p. 154) that the “wound was infected by MRSA resistant to other tested antibiotics.” In one of two case studies presented, they also note that after treatment by a few weeks, “Wound continues to improve, MRSA infection is not detected”. This, therefore, would suggest that the supplied phages contributed substantially to the dramatic wound healing in at least that case. This study is discussed further in Abedon [24,117].

### 4.15. Zhvania et al., 2017, S. aureus, Skin

Zhvania et al. [61] treated a 16-year-old male with Netherton syndrome for *S. aureus* skin as well as eye and nose infections. Treatment was with phage Sb1 as well as with Pyophage, a Georgia-sourced commercially available phage cocktail, though the latter was later updated. Daily topical treatment consisted of soaked gauze as well as phage-containing cream. Treatment initially took place over 20 days, then 14 days off, then another 20 days of phage treatment. It appears that antibiotic treatments were discontinued prior to the start of phage treatment and, indeed, antibiotic resistance was a primary factor in turning instead to phage therapy. Phages were delivered orally as well.

From p. 3, “Microbiological testing was done before intervention; qualitative bacterial cultures and culture sensitivities of the eyes, nostrils, and the worst, overtly infected lesions on the skin were obtained. At 3 and 6 months after the initiation of phage therapy, repeat swabs were obtained. The results showed a significant decrease in *S. aureus* in the eye and nostrils, while strong growth from the skin swab remained, indicating the need for prolonged treatment with periodic microbiological testing.” In addition, at 3 months resistance was seen to the Pyophage cocktail. A replacement cocktail therefore was used to which the bacterial isolates were sensitive. This was done for another 3-month period with the patient receiving phage treatment for 2 weeks alternating with 2-week breaks.

Their conclusion (p. 1) is that: “Treatment with several antistaphylococcal bacteriophage preparations led to significant improvement within 7 days and very substantial changes in his symptoms and quality of life after treatment for 6 months, including return visits to the Eliava Phage Therapy Center after 3 and 6 months of ongoing use of phage at home.” The initial improvement in symptoms and the development of resistance to one or more of the treatment phages, along with the likely lack of antibiotic co-treatment and some reduction in bacterial counts, is suggestive of a phage impact on the targeted bacteria.

### 4.16. Fadlallah et al., 2015, S. aureus Ocular Infection

Fadlallah et al. [62] treated a 65-year-old female for a corneal abscess caused by a *S. aureus*. The commercially available (in Georgia) phage, SATA-8505, was delivered both topically and intravenously for 4 weeks, with cultures negative 3 and 6 months later. Though treated with various antibiotics over the previous 11 years, there is no description of treatment with antibiotics during phage treatment. Therefore, it seems likely that the infection clearance was a consequence of the phage treatment. This case is discussed with additional detail in Abedon [14,24].

### 4.17. Lecion et al., 2013, S. aureus, Various Infection Types

Lecion et al. [63] present a primarily bacterial quantification following phage-treatment study. This was of fistula and purulent-wound, chronic *S. aureus* infections. Six patients were studied based on compassionate use, with titers ranging from 4 × 10^7^ to 6 × 10^8^ PFU/mL. Application was oral, via irrigation, and/or using wet compresses, one or more times each day for at least 4 weeks, with no additional antibacterial agents used. Greater than 1-log reductions in numbers of bacteria were seen in at least 4 cases, including a 3-log reduction during phage therapy for one patient. Clinical improvement was seen for half of the patients. Given the lack of concomitant antibiotic treatment, this study is suggestive of phage-mediated efficacy, particularly of patient 4, where bacterial counts were reduced from ~10^4^ to ≤10 CFU/mL. This study is discussed as well by Abedon [24].

### 4.18. Kvachadze et al., 2011, P. aeruginosa and S. aureus, Lung (with Cystic Fibrosis)

Kvachadze et al. [64] treated a 7-year-old female with cystic fibrosis using Pyophage cocktail. This was done once every 4 or 6 weeks via nebulization, for a total of nine treatments. *P. aeruginosa* in sputum was initially reduced from 8 × 10^6^ down to 7 × 10^3^ CFU/mL though then recovered during a period when phages were not being applied to 7 × 10^4^ CFU/mL. *S. aureus*, by contrast, was initially reduced only eight-fold to 1 × 10^6^ CFU/mL. For the latter result, the authors suggest that (p. 646), “This reflected a weak susceptibility of the strain to Pyophage in vitro.” Nebulization using phage Sb-1 along with Pyophage (5 treatments) reduced *S. aureus* down to between 10^3^ to 10^5^ CFU/mL, ultimately falling below detection limits. *P. aeruginosa* levels also further declined, down to 10^1^ to 10^2^ PFU/mL. Ambiguously, however, the authors mention (p. 647) a “medication-free month”. They also mention that antibiotic use, following phage therapy, was reduced by 50%. Though antibiotics thus appear to have likely been present during phage treatments, nonetheless, reductions in numbers of bacteria seem to have coincided with phage application rather than with prior to antibiotic application, suggesting a role for phages in treatment efficacy. This case study is also briefly discussed in Abedon [14,24,116].

### 4.19. Wright et al., 2009, P. aeruginosa, Chronic Otitis

Wright et al. [65] was a follow up to the Marza et al. [97] dog study (see also Hawkins et al. [120]). A total of 24 patients were treated in a randomized, double-blinded, United Kingdom-based phase I/II study for *P. aeruginosa*-associated chronic otitis. A six-phage cocktail (Biophage-PA; 6 × 10^5^ PFU in 0.2 mL for a titer of 3 × 10^7^) was supplied in a single dose to 12 patients in one ear, and a phage-less placebo dose was supplied to the other 12 patients. Current use of antibiotics was used as an exclusion criterion. Phage replication was found to occur in situ following application. Clinical improvement was seen with both the placebo and phage-treated group, though improvement was greater in the phage-treated group.

*Pseudomonas* counts were somewhat reduced in phage-treated individuals and were reduced to below detectable limits in three of the phage-treated individuals as measured on day 7. These remained not detectable in two of those individuals through day 42. For a total of two cases in the phage-treated group, however, “Not detected” was followed by CFU counts similar to those present prior to treatment. Furthermore, by day 42 there were three “Not detected” in the placebo group, including one where there was no bacteria detected on both days 21 and 42. On average, though, by days 21 and 42 post-treatment, CFU reductions in the treated group averaged approximately 80% lower than prior to treatment.

Overall, there appears to be reasonable hints of phage-mediated efficacy, including better clinical improvement and reduced bacterial loads. When elimination of CFUs occurred, it occurred sooner with the phage group. Given that this trial was done without associated antibiotic treatments, it appears reasonable to conclude that there were some phage-mediated efficacy effects. This study is discussed further in Kutter et al. [12] and also in Abedon [14,24].

### 4.20. Jikia et al., 2005, S. aureus Radiation Burn Infections

Jikia et al. [66] used PhagoBioDerm, an artificial skin impregnated with the Pyophage cocktail as well as with ciprofloxacin, to treat *S. aureus* infections of radiation burns of two male patients, 45 and 52-years-old. The *S. aureus* treated was ciprofloxacin-resistant but either “resistant” or “moderately susceptible” to gentamicin. After 23 days of intravenous gentamicin treatment along with ceftriaxone (etiology sensitivity not indicated for the latter), “…wound healing was only moderately successful in both patients, and purulent drainage was not eliminated… Moreover, *S. aureus* was consistently isolated from swab samples taken from both patients’ wounds.” (p. 25). After ~30 days following hospital admission, PhagoBioDerm treatment was initiated.

From p. 25: “Two days after PhagoBioDerm application, purulent drainage from both patients’ ulcers decreased significantly (to almost none), and they also noted a decrease in pain in their wounds. On the seventh day of treatment, the ulcers tested negative for *S. aureus*.” Though it cannot be ruled out that the antibiotics supplied intravenously and discontinued prior to the start of phage treatment were responsible for subsequent treatment success, it still seems likely, based on previous insufficiency of those treatments and the timing of improvement relative to phage introduction, that the phages in PhagoBioDerm played some role in that success. These cases are discussed in greater detail in Abedon [14,117].

### 4.21. Weber-Dąbrowska et al., 2000, Various Etiologies, Suppurative

Weber-Dąbrowska et al. [67] describe the treatment of 1307 patients between 1987 and 1999. Infections are described as suppurative and etiologies as multidrug resistant. This follows a report by Ślopek et al. [121], which covered the period from 1981 to 1986. In total, 85.9% of the treatments reported in the later report are described as having resulted in full recovery. An important summary is as follows (p. 548): “Noteworthy is that [bacteriophage] therapy was most effective in purulent meningitis and furunculosis (100% cured). High effectiveness was also noted in septicemia of different origin, purulent otitis media, suppurative peritonitis, pyogenic arthritis and myositis, osteomyelitis of the long bones, suppurative osteitis after bone fractures, pyogenic infections of burns, purulent mastitis and chronic suppurative fistulas.” It is uncertain from reading the article, however, that antibiotic treatment was discontinued upon the start of phage treatment, though the authors are clear in stating that (p. 548), “The results confirm the high effectiveness of bacteriophage therapy in combating bacterial infections that do not respond to treatment with the available antibiotics.” Thus, the study seems to be consistent with a role for phages in treatment successes. This article is discussed as well in Abedon [24,116,117].

## 5. Enzybiotics

In addition to the use of whole phages in phage therapy, substantial effort has been put into exploring the potential of phage-encoded enzymes, called enzybiotics, as stand-alone antibacterial agents. These enzymes can be divided into different categories based on various criteria, e.g., where they act under natural conditions, which is from within bacterial cells vs. from outside of bacterial cells, or what serves as their macromolecular substrate.

Acting from within bacterial cells are the endolysins, which are peptidoglycan hydrolases, e.g., [122,123,124,125,126,127]. These enzymes are used by phages at the end of their lytic infection cycles to destroy the bacterial cell wall and release of newly produced virions [128,129]. Endolysins can be recombinantly manufactured in pure form and applied from the outside of bacterial cells [130,131,132,133]. In the case of Gram-positive bacteria, these enzymes can directly reach and then digest the bacterial cell wall, resulting in osmotic lysis. To target Gram-negative bacteria, naturally occurring endolysins need to be engineered for them to be able to reach the cell wall, as it is protected by an outer membrane barrier [134,135]. Importantly, endolysins have been found to be nontoxic, rapidly acting, effective at killing targeted bacteria, and very difficult for bacteria to evolve resistance to, e.g., [136,137,138,139,140,141,142,143,144].

Various endolysin clinical trials have been or are being undertaken including two phase I trials (registry numbers NCT01855048 and NCT02439359), one phase I/II trial (registry number NCT02840955), two phase II trials (registry numbers NCT03089697 and NCT03163446), and one phase III trial (registry number NCT04160468), all of which target *S. aureus*. See for example Fowler et al. [144], who present results of a phase II, randomized, double-blind, intravenous, placebo-controlled, single-dose clinical trial of Exebacase endolysin used in combination with antibiotics (registry number NCT03163446). Effectiveness superior to antibiotics alone was noted, especially against methicillin-resistant *S. aureus* infections. See also Totté et al. [141] who present three dermatological *S. aureus* cases. In each case suppression of symptoms during topical application of using Staphefekt SA.100 was observed, and this was without concurrent antibiotic treatment in at least two of the cases. This endolysin preparation is available commercially in Europe. De Wit et al. [145], by contrast, were not able to demonstrate in their randomized, double-blind phase I/II, topical treatment study excess reductions in numbers of *S. aureus*, also with Staphefekt SA.100, over the placebo control. They speculate, however, that this could have been a consequence of recolonization by *S. aureus*, as was also observed by Totté et al. [141], once treatments had concluded.

Another group of enzybiotics are ectolysins [146]. These are phage-encoded, virion-associated peptidoglycan hydrolases which act from the outside of bacteria rather than from the inside under natural conditions. Ectolysins can be used similarly to endolysins, however, and both types of enzymes are generally described as lysins. We are aware of a single ectolysin, phase I/II, clinical trial (registry number NCT01746654, targeting *S. aureus*).

In recent years, another enzybiotic group has been intensively studied. These are phage polysaccharide depolymerase enzymes that can degrade bacterial capsular polysaccharides, exopolysaccharides, or lipopolysaccharide. They are relevant especially in terms of their potential to breakdown biofilm matrix, thereby either directly disrupting biofilms or increasing the potential for antibacterial agents to reach and impact biofilm bacteria [147,148]. These enzymes can be present on phage virions as tail spike proteins [147,149] and can also exist as free enzymes within phage-infected bacteria, which are then released upon lysis [149]. We are not aware of human studies using purified polysaccharide depolymerases, but in vitro anti-biofilm studies have shown promise [148,150,151,152].

## 6. Conclusions

All case studies involving phage treatments were conducted with an aim toward successful patient treatment rather than to supply evidence of phage-mediated therapeutic efficacy; hence, the often inclusion of antibiotics in addition to phages in those therapies. Nonetheless, generally speaking, it is difficult to accept that phage therapy done in conjunction with antibiotics, antibiotics to which the targeted bacterium is sensitive could constitute unequivocal proof of phage therapy efficacy. Therefore, other treatment approaches done in combination with phage therapies, such as debridement or irrigation of the bladder, could serve as possible complications in attributing anti-bacterial infection effectiveness to phages. We therefore consider independence from cotreatment effects to be a more important criterion for demonstration of phage therapy efficacy than even randomization and double-blinding of studies. Particularly important is independence of phage treatments from newly initiated applications of antibiotics to which the targeted etiology is sensitive. An exception is if phage-negative, antibiotic-only controls are provided, and these are found to be inferior to treatments including phages.

This latter point brings up the issue of use of proper negative-treatment controls, which essentially represents the primary concern of Eaton and Bayne-Jones [17] (p. 1848): “A consideration of many of the detailed case reports of what are stated by the investigators to be dramatic cures reveals that the process of recovery has not run an unusually swift course. Many of the conditions treated are self limiting, and, in those which are not, the usual methods of surgical intervention have been practiced. The fact that the patient recovered has, therefore, little significance.” Strictly speaking, clinical case studies do not tend to include parallel, no-phage-treatment controls. Alternatively, we might consider the relevance of previous experience in terms of spontaneous remission of infections—particularly if that experience has been well documented—when comparing the effectiveness of phage treatment. That is, with previous cases representing the equivalent to a nonphage-treatment control and especially dramatic differences considered to represent promising results.

It is our opinion that long durations of lack of success in the treatments of infections, such as treatments with antibiotics, which are followed by rapid or at least relatively rapid resolution of an infection upon initiation of phage treatment—especially given an absence of additional new treatments—should not be disregarded as possible indications of phage therapy effectiveness. This too, to a degree, was considered by Eaton and Bayne-Jones [17], p. 1848: “The speed of recovery as compared with other similar conditions not treated by bacteriophage may have some significance…” Ooi et al. [57], p. 727, also noted that “Patients… had failed all other conventional medical therapies and therefore served as their own controls,” whereas Fish et al. [40] states (p. S31): “…we compared our experimental phage treatments to previous treatments in each patient’s history. These individual historical controls, which showed an unsatisfactory response to conventional treatment, provided limited controls”.

Patey et al. [29] point out the importance simply of making sure that targeted bacteria are sensitive to the phages applied along with detection of in situ phage amplification. The latter—assuming no nontarget bacteria are also phage sensitive (e.g., commensal *E. coli* in the gastrointestinal tract)—may serve as an indicator of such sensitivity measured *during* treatments. Given these various considerations, in our opinion half of the studies discussed here that demonstrated anti-bacterial infection efficacy, including all of those reviewed in the main text of this article (Section 3 and Section 4), provide reasonably good evidence for phage therapy efficacy, with about one-third of the latter providing particularly good evidence (Section 3).

For the sake of easing future analyses of the human phage therapy literature, we hope to see more emphasis in publications on unambiguously describing (i) what antibiotics have been introduced in association with phage therapies, (ii) whether those antibiotics are new to the treatment or treatments being described, (iii) at what point in time those antibiotics are introduced especially relative to both the start of phage treatment and the timing of microbiological and clinical improvements, and also (iv) if targeted bacteria were found to be sensitive in vitro to both treatment phages and antibiotics prior to their application.

Lastly, we would like to reiterate the point that whenever antibiotics are administered in combination with phages, particularly antibiotics to which a targeted etiology is sensitive, there is always a chance that all of treatment success is due to this antibiotic use.

## Data Availability

No new data were created or analyzed in this study. Data sharing is not applicable to this article.

## Supplementary Materials

The following are available online at https://www.mdpi.com/article/10.3390/ph14111157/s1. Section S7: Insufficient evidence of phage-mediated efficacy. Section S8: Little or no efficacy observed. Table S1: Summary of clinical phage therapy studies, 2000 to present.
