# Supplementary material for: Phage Therapy in the 21st Century: Is There Modern, Clinical Evidence of Phage-Mediated Efficacy?
Source: Pharmaceuticals (Basel). 2021 Nov 13;14(11):1157. doi: 10.3390/ph14111157 (PMC8625828; doi:10.3390/ph14111157)
Supplement: Supplementary file 1 [file pharmaceuticals-14-01157-s001.zip › pharmaceuticals-1439557-supplementary/Supplementary Files/Supplementary Materials (002, as finalized).pdf]

pharmaceuticals

MDPI

Opinion

Phage Therapy in the 21st Century: Is There Modern, Clinical Evidence of Phage-Mediated Efficacy?

—Supplementary Materials

Stephen T. Abedon [1,*], Katarzyna M. Danis-Wlodarczyk [2] and Diana R. Alves [1]

[1] Department of Microbiology, The Ohio State University, Mansfield, OH 44906, USA; dianinha.alves@gmail.com
[2] Department of Microbial Infection and Immunity, The Ohio State University, Columbus, OH 43210, USA; danis-wlodarczyk.1@osu.edu
* Correspondence: abedon.1@osu.edu

Citation: Abedon, S.T.; Danis-Wlodarczyk, K.M.; Alves, D.R. Phage Therapy in the 21st Century: Is There Modern, Clinical Evidence of Phage-Mediated Efficacy? *Pharmaceuticals* **2021**, *14*, 1157. https://doi.org/10.3390/ph14111157

Academic Editor: Gill Diamond

Received: 12 October 2021
Accepted: 10 November 2021
Published: 13 November 2021

Publisher's Note: MDPI stays neutral with regard to jurisdictional claims in published maps and institutional affiliations.

Copyright: © 2021 by the authors. Licensee MDPI, Basel, Switzerland. This article is an open access article distributed under the terms and conditions of the Creative Commons Attribution (CC BY) license (https://creativecommons.org/licenses/by/4.0/).

In these supplemental materials we provide further discussion of human phage therapy articles, starting from the year 2000. The articles summarized below are ones which, in our opinion, do not supply sufficient evidence of phage-mediated anti-infection efficacy to have been included in the main text nor to have been included in either the first or second rows of Table 1. We present this material in a form similar to discussions of individual studies presented in the main text, however, and do so primarily to further justify the assertions presented in Table 1. As we note in the main text, the intention of these additional studies were generally not to provide proof of phage-mediated anti-infection efficacy, so discussion here rather than in the main text should not be construed as a criticism but instead serves only as an indication that either anti-infection efficacy was not observed or that efficacy could not be readily attributed, in our opinion, to the actions of treatment phages.

For completeness, we include all human phage therapy studies that we could identify, even if those studies do not provide evidence of anti-infection efficacy, or were even designed to provide such evidence. As noted also in the main text, as our primary aim is to ascertain whether evidence exists that phages are responsible for anti-infection efficacy, this is rather than our necessarily describing other aspects of articles such as whether phage treatments have been found to be lacking in side effects during safety studies. Two sections are presented, 7 and 8, that respectively correspond to studies in which treatment efficacy is evident but is difficult to conclude is phage mediated (row 3 in Table 1) versus studies in which it is difficult to ascertain treatment efficacy (row 4 in Table 1). In many of the latter cases, treatment obtaining treatment efficacy was not a primary aim of the study.

As also with discussions of individual studies in the main text, additional technical details of studies not provided in narratives may be found instead in Table S1. Lastly, note that we provide a separate reference list in this Supplemental Materials, as well as in association with Table S1, so reference numbering will differ between these as well as with what is found in the main text.

7. Insufficient Evidence of Phage-Mediated Efficacy

In this section we provide summaries of clinical phage-therapy studies that we have categorized in Table 1 as having a Phage Impact Score (PIS) of 0. Equivalent to the summaries provided in Section 3, our primary aim in this section is to justify our inclusion of

these studies in that category, which in this case indicates that we found it difficult to conclude with sufficient certainty that phages contributed to observed treatment efficacy.

7.1. Doub et al., 2021, S. epidermidis, prosthetic joint

Doub et al. [1] treated a 79-year-old female with an *S. epidermidis* infection via expanded access. A single phage dose was applied directly to the infection following surgical debridement. The infection began after surgical knee replacement (arthroplasty). Initial treatment consisted of 6 weeks of intravenous vancomycin but within 6 weeks following cessation of vancomycin treatment the infection recurred. This infection was subsequently considered to be recalcitrant since it again recurred following 2-stage revision surgery that involved prosthetic removal and 6 weeks of intravenous daptomycin therapy, as took place following the failure of vancomycin treatment. Continued joint culturing of *S. epidermidis* resulted in a further 6 months of treatment with doxycycline but with no improvement of the infection. The patient also suffered from aplastic anemia for which she was receiving biweekly transfusions of platelets and packed red blood cells. Chemotherapy was indicated but considered unsafe until the S. epidermidis infection had been successfully cleared.

Phage PM448—a bred phage [2] as well as recombinant product of multiple phage types—was obtained from PhagoMed in Austria, though in the laboratory the *S. epidermidis* target strain evolved resistance to this phage. Phage purification involved concentration using a centrifugal concentrator and dilution into phosphate buffered saline followed by endotoxin testing, resulting in <1 endotoxin units/dose. Following debridement as well as "modular exchange of the polyethylene line", a titer of 2×10^9 PFU/ml were locally applied, via injection within a volume of 10 ml of normal saline, into the intraarticular space surrounded the prosthesis. Culturing prior to phage application confirmed the continued presence of *S. epidermis*. Following surgery, daptomycin treatment was reinitiated intravenously in combination with newly introduced ertapenem, the latter due to a suspected *S. marcescens* contaminant that showed up in one culture. Oral rifampin was also initiated one week after surgery but was not continued due to patient nausea. Intravenous doxycycline was replaced with oral doxycycline 6 weeks after surgery, which was then continued indefinitely. Five months after surgery, "the patient has full range of motion of her knee and no clinical signs of [infection] recurrence". There were "no severe adverse events" that were seen in association with the phage treatment.

Independent of phage application, the circumstances leading up to the reinitiation of daptomycin intravenous treatment were not identical to those seen with the earlier treatment (an initial 2-stage revision surgery), at least as reported. It is possible in any case that daptomycin treatment alone during its second round of treatment was responsible for improvement in the patient's condition, and indeed intravenous daptomycin was supplied presumably in the hope that it would be efficacious in some manner. Therefore, it is difficult to conclude that the phage treatment alone was responsible for the observed infection improvement nor even that the phage contributed to improved daptomycin activity, though both are possibilities. Notwithstanding that conclusion, the patient's condition did improve following phage treatment, including in terms of their aplastic anemia, though it again is difficult to appreciate the degree to which the phage treatment contributed to those improvements.

7.2. Ferry et al., 2021, P. aeruginosa, prosthetic joint

Ferry et al. [3] compassionate-use treated an 88-year-old male for a *P. aeruginosa* prosthetic joint infection (knee) using a three-phage cocktail, supplied by Pherecydes, that consisted of 10^9 PFUs/ml for each phage (the three phages were PP1450, PP1777, and PP1792). Phage treatment was followed with intravenous ceftazidime and ciprofloxacin. These—acting alone, without phage co-treatment—previously had eliminated an equivalent infection in this patient for six months. Following phage treatment, also in combination with ceftazidime and ciprofloxacin intravenous treatment, and in each case in association with debridement toward implant retention, ciprofloxacin treatment apparently was never

stopped, rather than previously having been stopped after 12 weeks (p. 2): "The dose of ciprofloxacin [at six months?] was reduced to 250mg bid as suppressive antimicrobial therapy to prolong the remission of symptoms." At six months and one year (at which point the patient had died of unrelated causes) after this phage-antibiotic combination treatment, the knee remained free of infection. It is difficult to conclude, however, that continued ciprofloxacin treatment was not responsible for the preventing reoccurrence of the infection.

7.3. Lebeaux et al., 2021, A. xylosoxidans, lung

Lebeaux et al. [4] treated a 12-year-old male cystic fibrosis patient following lung transplantation for *A. xylosoxidans* present in the respiratory tract. This was done via compassionate use, starting on September 8, 2017. Phage treatment consisted initially of nebulization of 5 ml, three times daily, of a 4×10^9 PFU/ml (400 endotoxin units/ml). This initial phage treatment involved the use of a three-phage cocktail (phages 2-1, JWDelta, and JWT) administered over two days. Imipenem treatment was also initiated, about 40 days (July 31, 2017) prior to the start of phage treatment (September 8, 2017). Reduction in bronchoalveolar lavage counts of *A. xylosoxidans* did not change substantially over the course of this initial treatment.

A second phage application took place on January 23, with the patient still being treated with imipenem. Added to the original cocktail was a fourth phage, JWalpha. This treatment involved instillation of 30 ml into each pulmonary lobe during therapeutic bronchoscopy, with phage titer of 5×10^8 PFU/ml (and 176 endotoxin units/ml). This was then followed with nebulization at home with 5 ml, three times daily, for two weeks. Again, there were no reductions in bronchoalveolar lavage counts of *A. xylosoxidans* and imipenem treatment was discontinued, after phage treatment was discontinued, on February 16, 2018.

On February 19, 2018, there were still no obvious reductions in bronchoalveolar lavage counts relative to the start of treatments, though these counts had peaked slightly earlier at 10- to 100-fold higher on January 23 and February 8. Sputum counts were not reduced in association with the first phage treatment, but were 100- to 1,000-fold lower on March 5 and May 18, 2018, as well as on June 19, 2019, though were never measured at zero. Following the February 19 determination of bronchoalveolar lavage counts, the next determinations, on August 27, 2019, and April 30, 2020, found that these latter counts had been reduced to zero, roughly 1.5-plus years after the termination of phage and antibiotic treatment.

Though (p. 4) "the patient's respiratory condition slowly improved and oxygen therapy was stopped on 15 February 2018", the lack of documented improvement in *A. xylosoxidans* bronchoalveolar lavage counts until over a year after the cessation of both phage and antibiotic treatments make it difficult to conclude that phage treatment had been effective in this case. The study also obtained eight similar *A. xylosoxidans* isolates infecting this patient, including ones that were phage resistant, so it is possible that phage treatment had modified what *A. xylosoxidans* were present, resulting in improved conditions, but this conjecture we feel provides insufficient proof of phage-mediated anti-infection efficacy.

7.4. Leitner et al., 2021, various etiologies, urinary tract

Leitner et al. [5] applied phages directly into the bladder using a suprapubic catheter (intravesical therapy). This was done in combination with scheduled transurethral prostrate resectioning. See Leitner et al. [6] for initial publication of the study protocol. Patients were treated for 7 days either with 20 ml of Pyophage (twice daily, 28 patients receiving at least one dose, double blinded), a 20 ml of a placebo (also twice daily, with 32 patients receiving at last one dose, and also double blinded), or systemic antibiotics, the latter varying depending on etiology sensitivities (37 patients). Pyophage is normally employed to treat infections of the skin or wounds [7] but also urinary tract infections [8]. In this case, it was active against *Enterococcus* spp., *E. coli*, *P. mirabilis*, *P. aeruginosa*, *Staphylococcus* spp.,

and *Streptococcus* spp.; from p. 429, the phage product "was not produced under good manufacturing practice conditions but was subject to thorough monitoring and produced in accordance with the legislative guidelines of the Georgian Pharmaceutical Agencies."

The Pyophage cocktail also was supplemented with phages active against *Enterococcus* spp. with minimum titers of 10^4 PFU/ml, had a minimum titer of 10^5 PFU/ml for all other phages, and was modified with new phages over the course of the study. Neither minimum titer, however, could be considered to be high for phage therapy purposes [9]. Phages were suspended in and the placebo solution consisted of (p. 430) "standard bacteriology media and 0·9% saline, as well as quinazoline as a conservator." Instillations were in part patient administered with an intended retention in the bladder of one-half to one-full hour. Adverse reactions (usually sudden onset of fever) were lower with the phage treatment (21%) than in the placebo treatment group (41%) or in the antibiotic-treatment group (30%).

The approach used for this study is commendable due to a combination of both the randomized double blinding (at least for the phage and placebo treatments) and the explicit lack of antibiotic treatment during either the phage or placebo treatments, though the study did lack a fully no-treatment control. Neither phage nor antibiotic treatments were found to be superior to the placebo treatment in terms of efficacy, however, and treatment success was otherwise (p. 434) "unexpectedly low for both active treatment groups". The also unexpected effectiveness of the placebo was attributed to (p. 428) "mechanical reduction of the bacterial load following repeated bladder irrigation over 7 days." Therefore, efficacy cannot be attributed to the phage treatment over the span of treatments. It is possible that the phage treatment could have had a greater impact than the placebo if measured over longer time spans, though this was not reported.

7.5. Łusiak-Szelachowska et al., 2021, various etiologies, chronic rhinosinusitis

Łusiak-Szelachowska et al. [10] determined correlations between neutralizing serum antibody levels against treatment phages and compassionate use phage therapy efficacy. A total of 25 patients were treated topically (4) or both topically and orally (21), in both cases with locally produced phage preparations. A total of 8 of the treatments, or 32%, were deemed to have been successful, ranging from clinical improvement to etiology eradication. Though patients being treated all presumably possessed antibiotic-resistant infections, it is not obvious from the study whether antibiotics were used concomitantly with phages nor what antibiotics were used, if used, or what patient antibiotic use history might have been. Thus, though we are appreciative that the successful treatments likely were associated with phage action, there is insufficient information presented to conclude that there is proof of that activity.

7.6. Ramirez-Sanchez et al., 2021, S. aureus, prosthetic joint

Ramirez-Sanchez et al. [11] treated via compassionate use a 61-year-old female for an *S. aureus* infection of her right knee as associated with a prosthetic joint. Here ongoing systemic cefazolin treatment was unsuccessful in permanently resolving the infection, but co-treatment first with a three-phage cocktail and then with a single phage (SaGR51ø1) ultimately was successful. The cocktail was referred to as AB-SA01 and contained phages J-SA36, Sa83, and Sa87, which was delivered intra-articularly and then intravenously twice daily for two weeks, with a combined titer (as we calculate) of about 2×10^9 PFU/ml. This cocktail did not appear to directly result in successful *S. aureus* eradication and was discontinued due to a failure to obtain additional cocktail from the provider. The single phage, by contrast, was delivered also intra-articularly and then intravenously for six weeks, in this case with a titer of about 2.9×10^{10} PFU/ml. Although we are partly swayed by the observation that *S. aureus* was eradicated over longer periods (over a year) only upon the second round of phage treatment in combination with cefazolin, we note that two previous rounds of cefazolin treatment, one initially in combination with the phage cocktail, also resulted in temporary eradication of this bacterium (as according to their Figure 1). Thus, we are not sufficiently convinced that this study provides adequate proof

phage-mediated anti-infection efficacy, though we are attentive of the possibility that we may be being overly conservative in this assessment.

7.7. Rostkowska et al., 2021, K. pneumoniae, urinary tract

Rostkowska et al. [12] treated a 60-year-old male for a *K. pneumoniae* infection of the urinary tract, which had developed following kidney transplantation. Multiple antibiotic treatments were followed by infection relapses. Phages were applied in 10 ml volumes intrarectally, twice daily. After five days, meropenem co-treatment was initiated, which was found to be required for ongoing suppression of symptoms. Cysts in the left kidney were identified as the reservoir of the bacterium, and curing of the infection followed cyst removal. As the authors note (p. 4), "Due to the concomitant use of meropenem, it is difficult to assess the efficacy of [phage therapy] alone."

7.8. Tan et al., 2021, A. baumannii, lung

Tan et al. [13] treated an 88-year-old male with a carbapenem-resistant lung infection caused by *A. baumannii*. This was in association with chronic obstructive pulmonary disease. A single phage type, Ab_SZ3, was delivered by nebulizer for 30 min once daily for the first two doses and then every 12 hours for a total of 16 days of treatment. The phage was purified using either centrifugal filtration or cesium chloride density gradient centrifugation. Endotoxin units/ml were 10^5 and 10^4, respectively. Phage doses ranged from a calculated 10^6 PFU/ml to 10^{10} PFU/ml, as increased over the course of treatment. A maximum of 10^7 PFU/ml in bronchoalveolar lavage fluid was measured. Phage-resistant bacteria appeared early on over the course of treatment, though what fraction of total numbers of *A. baumannii* colony-forming units (CFUs) were phage resistant is not reported.

The *A. baumannii* strain being targeted was reported as being sensitive to tigecycline and polymyxin E. Tigecycline was supplied intravenously twice daily for the 6 days prior to phage treatment through until 5 days after the initiation of phage treatment. During this phage treatment, polymyxin E was supplied separately by inhalation. The study commendably supplies a timeline indicating exactly when these antibiotics were administered relative to phage treatments, though it is difficult from the supplied graph to ascertain exactly when the different treatments started and ended. Nonetheless, ending about a week prior to the start of phage therapy were intravenous treatments consisting of amikacin, ceftazidime, and ciprofloxacin. Overlapping the use of intravenous tigecycline were also cefoperazone and sulbactam. These treatments were then followed by five days of the polymyxin E delivery. Judging by the supplied timeline, the first day of indicated elimination of *A. baumannii* coincided approximately with the last days of treatment with tigecycline, cefoperazone, and sulbactam and/or the first days of treatment with polymyxin E. The authors acknowledge ambiguity with regard to phage impact, as resulting from overlapping of phage treatments with antibiotics treatments. They do propose, however, that the appearance of phage-resistant bacterial mutants could serve as evidence of a phage impact. We don't disagree with this latter point, but do feel that evidence is lacking in this study that phages reduced bacterial numbers sufficiently to indicate a potential to have contributed substantially to the curing of this infection.

7.9. Aslam et al., 2020, various etiologies, intravenous

Aslam et al. [14] describe the compassionate-use intravenous treatment of 10 patients, with a number of cases also reported individually [11,15–19]. Patients were infected by *A. baumannii* (2), *E. coli* (1), *P. aeruginosa* (5), and *S. aureus* (2) etiologies. These all were of infections that had been found to resist curing with antibiotic treatments alone. A total of 70% of treatments were described as successful. Emphasis in this section is on the five patients, including two failures, that had not previously been reported on.

Delays between requests for phage therapy and initiation of phage therapy ranged from 28 day to over one year, with a reported median of just under 6 months (these numbers are based on 7 more cases than the 10 cases focused on in the report). This delay likely

indicates a generally prolonged inability to obtain successful outcomes prior to the initiation of phage therapy, including successful outcomes as a consequence of spontaneous remissions. Personalized treatment was performed with new phages introduced in the case of development of phage resistance, though it is difficult to tell exactly what total phage numbers were applied. A fever in one patient (case 8) was reported following each of the two consecutive doses of phage cocktail but which did not return upon reducing the phage dose. Remarkably, 6 patients were treated via (p. 4) "self-administered [phage therapy] via an indwelling peripherally inserted central catheter." A fascinating finding was that in three cases (p. 7), "bacterial isolates emerged either during the course of treatment or thereafter with different antibiotic susceptibility profiles than the original infecting strain that were more amenable to antibiotic therapy."

Two of the newly reported-on treatments, both of infections of ventricular-assist devises, were deemed failures, as noted above. These had *P. aeruginosa* etiologies and failure was suspected to be chronic-biofilm related. Both of these patients developed bacteremias soon after the initiation of phage treatment, but also had histories of developing bacteremias independent of phage treatments. One other newly reported-on case ("Recurrent bacteremia and probable aortic graft infection", p. 7) involved *P. aeruginosa* infection and was deemed successfully treated. It is difficult to tell from the information provided whether it was phage therapy or instead ciprofloxacin co-therapy that resulted in this success, however. There was a reported recurrence of infection with antibiotic treatment alone, but no recurrence given combination with phage treatment. Lastly was a newly reported treatment of an *E. coli* urinary tract infection. Phage-antibiotic combination therapy but not prior antibiotic treatment alone was able to prevent recurrence.

All of these newly described cases unfortunately suffer from a combination of limited reporting detail and that (p. 3), "All active infections were treated with a combination of phage and systemic antibiotics." Proof of phage involvement in treatment success tends to be dependent on the timing of initiation of phage vs. new antibiotic treatments, but this information is not provided. Similarly, information on whether antibiotics used in combination with phages had been previously used, without equivalent success, is not generally presented. Nonetheless, assuming that these antibiotics which were co-administered with phages were not new to the patient, then these case studies would be suggestive of a phage role in contributing to the positive outcomes. This conclusion comes with a further caveat, however, that antibiotics presumably were co-administered with phages in these cases with at least a hope of direct impact on efficacy.

7.10. Doub et al., 2020, S. aureus, prosthetic joint

Doub et al. [20] describe the treatment of a 72-year-old male for a multi-year infection of a prosthetic joint with methicillin-resistant *S. aureus*. The treatment phage, SaGR51ϕ1, was stored as aliquots containing <1 endotoxin unit/ml as following cesium chloride centrifugation-based purification and subsequent dialysis. Discontinuation by the patient of two years of treatment with oral doxycycline was followed by infection recurrence. The infection then worsened despite resumption of doxycycline treatment, with leg amputation indicated. Prosthesis removal, debridement, and local treatment with tobramycin and vancomycin instead were undertaken. Phage therapy via expanded access was then initiated locally (intra-articularly) with 5×10^9 PFU suspended in 10 ml of normal saline) and daily intravenously phage treatment (3×10^9 PFU suspended in 50 ml of normal saline) started on the following day and continued for 3 days. At the same time, intravenous daptomycin treatment was initiated and maintained for 6 weeks, where improvement was observed. For the sake of prevention, the patient received two additional intra-articular phage treatments despite showing no signs of infection (cultures were negative). Combined treatments resulted in clearance of the infection. As a number of antibiotics were applied concurrently with phage treatment, however, it is difficult for us to conclude with certainty that the phages were truly responsible for the observed treatment efficacy.

7.11. Ferry et al., 2020 (two separate publications), S. aureus prosthetic joint

Ferry et al. [21] treated, in a context of compassionate use, a 49-year-old male for a *S. aureus* prosthetic knee infection. A two-phage cocktail that was delivered locally within a hydrogel, as following tissue debridement. The two phages, PP1493 and PP1815, were supplied by Pherecydes Pharma, with the latter of the two phages deemed "less active" against the targeted bacterial strain by a variety of criteria, as determined *in vitro*, but this phage was used for treatments anyway. Titers upon mixing into the hydrogel were a little over 10^9/ml, in combination. Unfortunately, complications ensued and it consequently is difficult to infer to what extent phage treatments were efficacious, if at all, in this case.

Ferry et al. [22] treated three patients following debridement of *S. aureus* prosthetic knee infections. A three-phage cocktail of titers diluted to a reported 10^9 PFU/ml and also supplied by Pherecydes Pharma was applied locally. For each case, the etiology was found to have "high susceptibility" to at least two of the three phages. The authors report (p. 4) that "the outcome was favorable with a significant and impressive clinical improvement of the function for all patients", though subsequent complications are reported. Treatment with a variety of antibiotics immediately followed the start of phage treatment, however, so it is difficult to conclude (as the authors concur) that this treatment success was a product of phage action, except that these patients had previously been treated with antibiotics alone with less favorable outcomes, so similar outcomes therefore were expected. We agree that the results of these phage treatments are encouraging, but just not providing sufficient proof of phage-associated efficacy.

7.12. Gainey et al., 2020, Achromobacter spp., lung with cystic fibrosis

Gainey et al. [23] treated a 10-year-old, female patient—with cystic fibrosis—under the auspices of expanded access. This was done with a combination of a phage (Ax2CJ45φ2, supplied by Adaptive Phage Therapeutics), cefiderocol, and meropenem-vaborbactam, targeting an *Achromobacter* spp. Treatments initially consisted of 2 weeks of antibiotic alone, delivered intravenously, and then 2 weeks of bacteriophage alone, also delivered intravenously. Forced expiratory volume improved during the antibiotic treatment, but then declined during the phage treatment. The patient continued to be *Achromobacter* spp. positive, including with isolates that were resistant to the treatment antibiotics. A subsequent 2-week treatment with both antibiotics and phages, however, resulted in her sputum becoming *Achromobacter* spp. negative as determined 8 and 16 weeks following the end of this treatment. It did not appear, however, that forced expiratory volume during treatment (60%) improved substantially beyond that seen previously during antibiotic treatment alone (58%). We speculate nevertheless that phage treatment may have been effective at least in reducing numbers of target bacteria that were resistant to the treatment antibiotics. Nevertheless, we are unsure of the phage numbers which were able to reach the targeted bacteria, so consequently are not confident in the pharmacokinetics that would need to have underlain that result.

7.13. Petrovic Fabijan et al., 2020, S. aureus, intravenous

Petrovic Fabijan et al. [24] treated 13 patients for various *S. aureus* infections intravenously. This was done twice daily for 2 weeks using a three-phage cocktail (dubbed AB-SA01, as produced via good manufacturing practice). Given a dosage of 3×10^9 PFU, a combined titer of less than 10^6 PFU/ml of blood as present post-phage delivery is estimated, which should *not* be considered to be substantial for phage therapy purposes [9]. Patients are described as "seriously ill (premature death reasonably likely)", with 6 patients in total dying, including three who died at 15, 27, and 90 days after the completion of therapy.

Co-treatment was with various antibiotics and the authors note (p. 468) that, "the observed decline in inflammatory responses that occurred shortly after bacteriophage administration… may also reflect the 4–10 d of preceding antibiotic treatment in our cohort." It is possible that the supplied phages may have had localized impacts following replication in the presence, e.g., of bacterial biofilms [25]. The (1) relatively low numbers of phages delivered, (2) low numbers of bacteria present in the blood at the point of phage

application (~100-fold lower than concentration of phages in the blood), (3) the use of antibiotic co-treatment, and (4) a lack of reporting of prior antibiotic use, makes it difficult to conclude that phage cocktail played a substantial role in observed patient improvements. The study, however, was designed as a safety rather than efficacy trial.

7.14. Qin et al., 2020, K. pneumoniae, urinary tract

Qin et al. [26] treated a patient with a *K. pneumoniae* urinary tract infection. One to four phages from a collection of five phages (ΦJD902, ΦJD905, ΦJD907, ΦJD908, and ΦJD910) were used, with the first round of treatment using one phage, the second two, the third three, and the fourth four. Phage resistance was observed to develop among what was found to be a polyclonal *K. pneumoniae* infection. Phages were applied at 5×10^8 PFU/ml with the bladder irrigated with 50 ml every 48 h and the kidney with 10 ml, the latter via a percutaneous nephrostomy.

Antibiotic treatments were mostly interspersed among phage treatments, though in one case these were overlapping. For these three separate rounds of antibiotic treatment, a different combination of antibiotics was used for the middle round (amikacin and imipenem) vs. those used for the first and last antibiotic treatments (piperacillin-tazobactam). In looking at the timeline of treatments and culture positivity, for two rounds of treatments cultures became negative at the end or after the end of treatments rather than during phage treatment, whereas in another instance cultures became negative on the first day of phage treatment. Alternatively, in two instances cultures because negative at or approximately at the beginning of antibiotic treatment. Relevant to the recurrence of positive cultures between treatment episodes, the authors speculated (p. 7), that "heterogeneous pathogens in the renal pelvis were unreachable by phage cocktails via bladder irrigation." This led to renal pelvis irrigation with phages during what appears to have been the final round (of four) of phage treatment.

Though it is true that antibiotic and phage treatments mostly did not coincide, still it is difficult to interpret from the presented pattern of treatments and culture-negative results that phage therapy was responsible for declines in bacteria presence, unless phage treatment was intentionally discontinued at or about at the point that cultures because negative. Furthermore, successful elimination of the infection coincided with the third round of antibiotic treatment, which was only followed after about one month with phage co-treatment, the latter including via renal pelvis irrigation. Clearly as the same antibiotic treatment previously had not been successful in eliminating the infection (first round of antibiotic treatment), this does suggest that the phage treatment, perhaps particularly upon phage delivery to the renal pelvis, was crucial in curing this infection. Still, it could be instead that the antibiotic treatment during the third round of antibiotic application simply happened to be successful that time. Given the substantial overlap between phage and antibiotic treatments along with lack of association of phage treatments with observations of bacteria-negative cultures, it is difficult to conclude that this study provides sufficient evidence of phage anti-infection activity.

7.15. Aslam et al., 2019, P. aeruginosa and B. dolosa, lung transplant

Aslam et al. [17] treated, under expanded access, three lung-transplant recipients with phages in combination with antibiotics for lung infections with *P. aeruginosa* (two) or *B. dolosa* (one). Some bacterial resistance to phages was noted, but etiology sensitivity to co-administered antibiotics makes it difficult to conclude that substantial reduction in bacterial counts was seen as a consequence of phage action. Four treatment phage cocktails were obtained from a variety of sources and delivered intravenously (5.3×10^5 to 5×10^9 PFU; 0.2 to 200 endotoxin units/ml). Dosing intervals ranged from once every 2 hours to once every day.

Patient 1, a 67-year-old male, was infected with *P. aeruginosa* and experienced two episodes of pneumonia, both of which were treated with phage cocktails, both intravenously and by nebulizer. The first phage treatment was in addition to concurrent colistin

and piperacillin-tazobactam treatments, with the etiology deemed resistant to piperacillin-tazobactam while possessing only intermediately sensitive to colistin. Clinical improvement was seen after two weeks. It is possible that the only intermediate sensitivity of the etiology to colistin points to a phage role in eliminating the infection. This in combination with the isolation, post-treatment, of a *P. aeruginosa* strain that was resistant to all four phages in the original cocktail is suggestive of at least some phage impact. As proof of significant phage contribution, it would have been helpful to have a quantitative sense of the frequency of these bacterial mutants rather than that they were simply present, however.

Also for patient 1, there was a subsequent *P. aeruginosa* lung infection, with a strain possessing a different antibiotic-resistance pattern. The cocktail was modified with the addition of a new phage due to culturing of a phage-resistant isolate detected. In addition, tobramycin was added to the antibiotics previously used. As the infecting bacterial strain during the second episode was fully susceptible to tobramycin, this suggests a possibly diminished role for the phages in infection improvement, though phage-resistance was found to have developed.

Patient 2, a 57-year-old female, was treated with the same phage cocktail as patient 1 for four weeks, but only intravenously. She was treated as well with colistin, to which the infecting *P. aeruginosa* was fully sensitive. *P. aeruginosa* was no longer cultured from bronchoalveolar lavages once treatment had begun nor 60 days after completion of treatment. The treatment with colistin of a colistin-sensitive etiology, however, makes it difficult to conclude that the phage treatment contributed to her recovery.

Patient 3, a 28-year-old female, was infected with a *B. dolosa* strain that was fully sensitive to minocycline. She responded favorably to treatment with ceftazidime-avibactam and piperacillin-tazobactam, though the etiology otherwise was described as resistant. The same antibiotic regimen was initiated following a subsequent bout of pneumonia, with phage BdPF16phi4281 added after one week. Her condition improved after the start of phage treatment. The favorable response to the first round of antibiotic treatment, however, was not instantaneous, i.e., "She eventually responded favorably…" Therefore, without additional detail as to the time course of improvement, it is difficult to conclude that the phage played an important role in the subsequent improvement. Furthermore, the *B. dolosa* infection was never eliminated and the patient eventually died.

7.16. Gupta et al., 2019, various etiologies, chronic wound

Gupta et al. [27] studied the treatment (p. 1) of "Patients with chronic nonhealing wound not responding to conventional local debridement and antibiotic therapy", ages 12 to 60 years old. Three types of bacteria were targeted, *E. coli* (6 patients), *P. aeruginosa* (9 patients), and *S. aureus* (5 patients). Wounds were at least 6 weeks old at the time of treatment and bacterial counts were reported in excess of 10^6. Though presumably the latter is in CFU units, it is difficult to tell what the denominator of these measurements is. The authors note that (p. 1), "After confirmation of organism, a cocktail of customized bacteriophages [isolated by the authors] was topically applied over the wound on alternate days till the wound surface became microbiologically sterile." The sterility was seen 9 days post the start of phage treatment for 9 patients and after 13 days for the rest. Application was of 100 $\mu l/cm^2$ of 10^9 PFU/ml phage cocktail every other day. A total of 3 to 5 phage doses were applied and complete healing, by day 21, was seen with seven patients, whereas (p. 1) "in others healthy margins and healthy granulation tissue were observed". There unfortunately is no indication in the study of whether antibiotic treatment was discontinued prior to phage application, nor how long antibiotic treatment had been attempted prior to phage application. Therefore, though the results of complete sterility for all patients following phage treatment along with various degrees of wound healing would seem to be highly promising, it is difficult to conclude from the supplied information that it was phage treatment that was responsible for the positive results

7.17. Law et al., 2019, P. aeruginosa, lung with cystic fibrosis

Law et al. [19] performed intravenous anti-*P. aeruginosa* phage therapy on a 26-year-old female, with cystic fibrosis, who was experiencing pneumonia. Phage AB-PA01, supplied and produced using good manufacturing practices by AmpliPhi, was administered intravenously 4 times daily for 8 weeks at doses of 4×10^9 PFU/ml. Azithromycin, ciprofloxacin, colistin, doripenem, linezolid, and piperacillin–tazobactam were administered also, though azithromycin treatment began well prior to the start of phage treatment and treatment with colistin and linezolid was discontinued soon after phage treatment was initiated, according to their Figure 1. Initial use of ciprofloxacin was just prior to the start of phage treatment. The latter was then replaced with doripenem after 3 weeks, which had also been briefly used previously. Ciprofloxacin then replaced doripenem after about 7 weeks.

Patient oxygen requirements peaked at 70 liters/min a few days prior to the start of phage treatment, dropped to 30 liters just prior to the start of phage treatment, and then dropped to 10 liters/min after 1 week of phage treatment, before gradually declining to about 5 liters/min after 6 weeks of phage treatment. A majority of the reduction in oxygen requirements, from 70 to 30 liters/min, thus occurred just prior to phage treatment. There was no recurrence of *P. aeruginosa* pneumonia following phage treatment, and the targeted etiology remained mostly phage sensitive throughout the treatment period.

Assessment of the contribution of phages to the observed treatment success is complicated by the numerous concurrent antibiotic treatments, though a table of antibiotic sensitivity of the targeted bacteria is supplied in their Supplemental Materials. On days -4, 8, and 73, intermediate sensitivity to ciprofloxacin is reported, as well as full sensitivity also on day -4. On days 8 and 14, full sensitivity to doripenem is reported. Thus, during the first week of phage treatment, when oxygen requirements were reduced to 10 liters/min from 30 liters/min, it is possible that ciprofloxacin played an important role. Similarly, when doripenem was introduced, there may have been fully sensitive bacteria present, though at best there were only minor reductions in oxygen needs while these doripenem-sensitive bacteria were being eliminated. Oxygen needs peaked after colistin treatment was discontinued, prior to the start of phage treatment. Colistin treatment was briefly restarted, stopped, and then restarted again for a few days. This latter round of colistin treatment overlapped with the initial dramatic decline patient oxygen needs, from 70 to 30 liters/min, and during this time there possibly was a decline in *Pseudomonas* colistin sensitivity. In addition, linezolid application overlapped the latter colistin treatment, also coinciding with the noted dramatic improvement, though no data is provided as to sensitivity of isolated *P. aeruginosa* to linezolid. Initial ciprofloxacin treatment also overlapped with this period of dramatic decline in oxygen needs, as occurred prior to the start of phage treatment. Lastly, piperacillin–tazobactam was supplied starting 1 week prior to phage treatment, with isolated *Pseudomonas* displaying both sensitivity and resistance throughout phage treatment.

Overall, we find it difficult to conclude from the information provided in this publication that the noted patient improvement was a consequence of phage action. It is possible in particular that the newly introduced ciprofloxacin, acting on an otherwise somewhat antibiotic-impacted population of targeted bacteria, was responsible for the declines in oxygen needs from 30 to 10 liters/min, particularly as they occurred following the cessation of colistin, doripenem, and linezolid treatments. Alternatively, it is at least possible that phages were involved in further reductions in oxygen needs after ciprofloxacin was initially discontinued, though the evidence here for a phage role—vs. spontaneous improvement or instead a role for doripenem, or even azithromycin—is not strong.

7.18. Onsea et al., 2019, various etiologies, osteomyelitis

Onsea et al. [28] describe the compassionate-use treatment of osteomyelitis using combinations of phages and antibiotics, of a total of four patients. From p. 2, "These patients had a poor prognosis (i.e., need for amputation) after multiple failed medical and

surgical therapy regimens…" Infections were subject to debridement, irrigation with sodium bicarbonate solutions, and then various means of holding liquid phage preparations in place. Treatment phage types as well as the number of treatment phage types (BFC1 or Pyophage cocktails) varied depending on the infecting etiologies. Per patient, the latter consisted of (1 and 2) *P. aeruginosa* and *S. epidermidis*, (3) *Streptococcus agalactiae* and *S. aureus*, and (4) *Enterococcus faecalis*. Phage treatments lasted 7 to 10 days and patients were deemed to be free of the original infecting bacteria for over 8 to 16 months, depending on the patient. Though some evidence is presented of the susceptibility of targeted bacteria to treatment antibiotics, missing from the report are what specific antibiotics were used that failed to cure infections *prior to* phage application, in comparison to those antibiotics used in combination with phages. Though we recognize that previous efforts to cure these infections were unsuccessful, nevertheless it is difficult to conclude, from the information presented, that it was phages rather than concurrently used antibiotics that were responsible for the treatment successes.

7.19. Tkhilaishvili et al., 2019, P. aeruginosa, prosthetic joint

Tkhilaishvili et al. [29] treated an 80-year-old female for a combination of prosthetic knee infection and osteomyelitis. These were thought to be caused by *P. aeruginosa* but with other bacterial species found to be present as well (*K. pneumoniae*, *Providencia stuartii*, *S. epidermidis*, and to some degree *S. haemolyticus*). Compassionate use of phages was applied locally both during surgery (100 ml) and after (5 ml of 10^8 PFU/ml, as applied through four drains, three times daily for 5 days). These phages were supplied by the Eliava Institute in Tbilisi, Georgia. The surgery involved removal of the prosthesis, debridement, and rinsing with sodium bicarbonate.

Following surgery, ceftazidime, colistin, and meropenem treatments were also initiated, but prior to these treatments the *P. aeruginosa* etiologies (two distinct strains) were shown *in vitro* to be resistant to meropenem and one strain was found to be resistant also to ceftazidime. Gentamicin and clindamycin were also associated with a spacer used to temporarily replace the prosthesis, but the *P. aeruginosa* either were or likely were resistant. By contrast, both *P. aeruginosa* strains were determined to be sensitive to colistin.

P. aeruginosa could not be cultured from drainage fluid following the start of phage therapy (on days 3, 4, and 5). We wonder, though, whether the high numbers of phages applied could have interfered with the culturing of *P. aeruginosa* [30,31], though commendably fluid collection was done prior to daily phage instillation rather than after. Subsequent reimplantation of the prosthesis was successful, with no apparent reoccurrence of the infection over at least 10 months. Notwithstanding this success, we find it difficult to rule out a key role for colistin and perhaps also ceftazidime in bringing the infection under control.

7.20. Duplessis et al., 2018, P. aeruginosa, bacteremia

Duplessis et al. [32] treated a 2-year-old male for a *P. aeruginosa* infection complicated with bacteremia or sepsis. A U.S. Naval Medical Research Center-supplied phage cocktail consisting of two phages was administered intravenously with a dose of 3.5×10^5 PFU, four times daily. This inititally was limited to a total of 6 doses before being reinitiated 12 days later. Blood cultures became sterile 5 days after the first treatment, reverted to positive, became sterile again one day after the second treatment, and then again became positive. Possibly spontaneous blood culture sterility was also seen twice, i.e., as not seemingly associated with immediately previous phage treatment. In addition, the amount of phages supplied can be described as quite low [9], and likely were insufficient to result in blood sterility unless phage replication was occurring *in situ* in association with relatively high concentrations of bacteria. That possibility, however, was not explored. As ultimately the infection was not cured, and it is not straightforward to connect phage treatment to subsequent episodes of blood sterility, it is difficult to conclude that positive effects could be attributed to phage use, even though as reported the blood sterility was only seen after phage treatment had originally been initiated.

7.21. Ferry et al., 2018, P. aeruginosa, joint

Ferry et al. [33] treated a male in his early 60s for an infection (fistula) located in his sacroiliac joint of his pelvis. Phage treatment, using a four-phage cocktail supplied by Pherecydes Pharma and generated using good manufacturing practices, was combined with debridement and use of Ceftolozane-tazobactam. Phages were locally applied to the infection first as 10 ml of liquid preparation and then with the same delivered within compresses infused with 20 ml containing perhaps roughly 5×10^8 PFU/ml. This treatment was undertaken on every third day for a total of four phage treatments. Colistimethate was also supplied locally. From p. 2902, "At the time of surgical reconstruction at day 14, the macroscopic aspect was extremely favourable, and muscle and skin and soft tissue flap reconstruction was performed. No bacteria grew in the culture, and healing was rapid." Unfortunately, the patient died on day 45 for reasons unrelated to the infection. In terms of the role of phages in the infection's curing, it is difficult to differentiate their impact from that of the co-administered antibiotics.

7.22. Ferry et al., 2018, P. aeruginosa and S. aureus, prosthetic joint

Ferry et al. [34] treated via compassionate use an 80-year-old female with relapsing infections of her right-hip prosthetic joint. A six-phage cocktail was provided by Pherecydes Pharma (AB-SA01) containing three phages each targeting *P. aeruginosa* and *S. aureus*, two of which were later found to be active against an *S. aureus* strain isolated from the infection. These were injected once into the infected joint, prior to joint closure (a video of the injection is found in their supplemental materials). At the time, *P. aeruginosa* could not be cultured from the infection though methicillin-susceptible *S. aureus* was still present along with *E. faecalis* and *Staphylococcus lugdunensis*. Co-treatment with amoxicillin, clindamycin, and daptomycin were also undertaken, and later ciprofloxacin was added as well. During an operation subsequent to the start of this treatment, *S. aureus* could not be cultured. An absence of clinical signs of infection was noted at 18 months following phage treatment. As *S. aureus* can be susceptible to all four of the antibiotics administered, with the *S. aureus* strain being targeted sensitive to methicillin, it is difficult to determine what role if any the phages themselves played in patient recovery from this infection.

7.23. Fish et al., 2018, S. aureus, diabetic toe ulcers

The Fish et al. [35] is a continuations of the Fish et al. [36] study. By contrast to the 2016 study, this 2018 study did involve brief antibiotic treatment. From p. 3 of that publication: "A week into [phage] treatment, the erythema seemed to be increasing, suggesting the infection was worsening. At this time, she was also started on levofloxacin 500 mg, to which the bacteria were sensitive. At day seven, we noted no changes in the amount or intensity of the erythema or reduction of edema, suggesting that the antibiotic was not helping, so it was discontinued. No further antibiotics were given." Thus, it seems likely that resolution of the infection in this case probably was associated especially with phage action, but we cannot be truly certain of this due to the introduction of antibiotic treatment to which the etiology was sensitive.

7.24. LaVergne et al., 2018, A. baumannii, surgical wound

LaVergne et al. [16] treated a 77-year-old male with phages for an *A. baumannii* infection that arose following locally following cranial surgery. Antibiotic treatment prior to phage treatment was not successful and antibiotic treatment appears to have been stopped one day after phage treatment was started. The phage used was supplied by the Naval Medical Research Center-Frederick and applied intravenously with a titer of 2×10^7 within 4 ml, 12 times daily for 8 days (350 endotoxin units/ml). This resulted in roughly 10^0 to 10^2 PFU/ml observed in the blood, which in principle might be adequate for treating localized bacterial infections, assuming substantial local amplification of phage numbers [25], but would not be sufficient as an antibacterial treatment without such amplification [9], as indeed the authors acknowledge. Following phage treatment, signs of infection neverthe-

less ceased (p. 3): "…no further signs of infection at the craniotomy site after surgical debridement, and no purulence…" The authors concluded, however, that there was a "lack of response to bacteriophage therapy in our patient…" The patient died 1 day after the cessation of phage therapy.

7.25. Patey et al., 2018, S. aureus, chronic otitis

Patey et al. [37] describe the treatment via compassion use of a patient with chronic otitis associated with *S. aureus*. The description is brief (p. 8): "…bacteriophage suspension, active *in vitro* against the patient isolate, in combination with pristinamycin. Within 48 hours, the patient noticed a clear improvement: the cessation of purulent flow and pain. Subsequent consultations confirmed a favorable course: the absence of otorrhea or pain and disappearance of *Staphylococcus*. After three months, the ear examination was still very satisfactory and the treatment was stopped." Given the co-administration of phages with pristinamycin, of which no prior use is described, it is however difficult to conclude that this favorable outcome was a consequence of phage action. A total of 15 chronic cases that received phage treatment under compassionate use are outlined in a table, but the authors note that these treatments (p. 11) were always "accompanied by antibiotic therapy".

7.26. Ujmajuridze et al., 2018, various etiologies, urinary tract

Ujmajuridze et al. [38] treated urinary tract infections following prostate resection, with *Enterococcus* spp., *E. coli, P. aeruginosa, or Streptococcus* spp. targeted. An adapted Pyophage cocktail was used to treat 9 patients, with phages instilled into bladders twice daily at 20 ml per treatment for 7 days, and with retention for one-half to one hour. It does not appear that antibiotics were used as co-treatments other than with one patient for whom cephalosporin treatment was started on day 3 of phage treatment due to development of a fever (patient 9). Reductions in CFUs/ml as determined after phage treatment ranged from none to at least 3 logs, but it is challenging for us to reconcile data presented in their Table 1 with descriptions made in their text. In addition, given the results of Leitner et al. [5], it is difficult to conclude that the phage treatment, vs. simply instillation of the phage preparation into the bladder, was always responsible for all treatment success that was observed. The reductions from 10^7 to 10^5 (patient 1) and to 10^4 (patient 2) CFUs/ml of *E. coli*, however, are nonetheless suggestive that at least some phage-mediated treatment success was seen in this study.

7.27 Schooley et al., 2017, A. baumannii prostate infection

The Schooley et al. [15] study is reviewed in detail by Abedon [39]; see section 4 there, starting on p. 33. Overall, for reasons of equivocal association of the timing of phage treatment with that of symptomatic improvement, along with modification of antibiotic therapy early during phage treatment, as well as temporary discontinuation of phage treatment also early during therapy, it is difficult for us to conclude that treatment success can with certainty be attributed to phage anti-bacterial infection activity. Also in Abedon [39] is discussion of some of the pitfalls of using the isolation of phage-resistant bacteria as a marker of possible substantial phage contribution to treatment efficacy.

7.28. Kutateladze, 2015, various etiologies, various infection types

Kutateladze [40] is a summary of thousands of patient visits to the Eliava Phage Therapy Center that occurred between 2012 and 2014. The article reports that 95% of treatments resulted in (p. 81) "significant improvement and recovery". There is insufficient detail provided, however, to conclude from that article that this improvement was a consequence of phage action, though to the extent that antibiotic co-treatments were not used then such phage anti-bacterial infection action could very well be the case. This article is discussed further in Abedon [41].

7.29. Khawaldeh et al., 2011, P. aeruginosa, urinary tract

Khawaldeh et al. [42] compassionate-use treated a 67-year-old female with a six-phage cocktail for a *P. aeruginosa* urinary tract infection. Phages were instilled through a catheter into the bladder twice daily for 10 days (20 ml of ~10^7 PFU/ml, held for 30 min). Colistin and meropenem treatments were initiated on day 6, with the infection not previously treated with colistin. Bacterial viable counts were reduced ten-fold on day 1 and *P. aeruginosa* DNA was reduced about 100-fold, although DNA reductions were not consistently seen. Bacterial counts ultimately were reduced to zero, but this was only observed after the addition of antibiotics to the treatment. Overall, the introduction of colistin to the treatment, as well as the findings of Leitner et al. [5] as discussed above that bladder irrigation alone can have beneficial impacts on bacterial loads, together argue against this study providing convincing evidence of substantial phage-mediated anti-bacterial efficacy. Nevertheless, phages may have been replicating within the bladder before *P. aeruginosa* densities were reduced. This study is discussed as well by Abedon [39,41].

7.30. Marza et al., 2009, P. aeruginosa, burn wound

Marza et al. [43] describe the treatment of a burn wound that showed no improvement in a *P. aeruginosa* infection for several months. Phages were applied using 25 mm sterile filter-paper discs to which 0.2 ml of 10^3 PFU were added. Phage numbers were found to increase *in situ* following their application (suggesting replication by the treatment phages). Bacteria also were eliminated and a subsequent skin graft was successful. It is unclear from the text to what extent antibiotic may have been used as a co-treatment, however (p. 645): "Subsequently extensive grafting was successful, but the patient was receiving intravenous ceftazidime at the time, which might have been responsible for the improvement." Thus, it is uncertain whether phages were responsible for the treatment success.

7.31. Weber-Dąbrowska et al, 2006, Staphylococcus spp., otitis media

Weber-Dąbrowska et al. [44] present a case report of treating a 24-year-old female for otitis media associated with culturable *S. epidermidis* and *S. homis* as found in separate ears. Initial treatment was with the antibiotics, atecortin and dicortineff, as well as the steroid, elocon. Oral treatment with phage 676/T was then attempted, all without success. Treatment with two phages specific to *Staphylococcus*, A3/R as well as 676/T, was then commenced both orally and topically, at least one-half month after conclusion of the previous phage treatment, and was continued for 3 weeks. From p. 242, "Although some improvement in health condition was registered by endoscopic examination, the patient complained of persisting pain and discomfort." Symptoms were relieved upon subsequent bovine lactoferrin treatment. Though it is probable that antibiotic treatment was discontinued prior to the start of phage treatment, nevertheless the impact of phage treatment was at best inadequate, suggesting little evidence of phage-mediated efficacy in this case.

7.32. Markoishvili et al, 2002, various etiologies, wound

Markoishvili et al. [45] report on the use of PhagoBioDerm, an artificial skin impregnated containing Pyophage as well as ciprofloxacin, on 96 patients treated. Over time courses ranging from 6 days to 15 months it was observed that a total of 70% were found to have recovered completely and 25% showed improvement. It is unclear, however, that

the ciprofloxacin alone was not responsible for these results. See, however, the results of Jikia et al. [46], as discussed in the main article, which also used PhagoBioDerm. Additional summary of this study is found in Abedon [39,47].

7.33. Weber-Dąbrowska et al., 2001, various etiologies, various infection types

Weber-Dąbrowska et al. [48] describe the treatment of bacterial infections of cancer patients using phages. Phages were obtained from their own collection and were purified using a number of steps, though not including explicit removal of endotoxin. Infections were associated with a single etiology (n = 12) or were mixed infections (n = 8). Previous treatments were with antibiotics but without success. Treatment was either oral or oral in combination with local treatments. The authors indicate that (p. 131) "Cure of infection was achieved in all cases". Assuming that antibiotic treatments were discontinued (this is not explicitly specified), or based on the lack of previous success using antibiotics, then it is likely that the supplied phages mediated the resulting efficacy.

7.34.Weber-Dąbrowska et al, 2000, various etiologies, suppurative

Weber-Dąbrowska et al. [49] explored the impact of phage therapy on the production of cytokines, though with efficacy data included as well. Most infections were with *Staphylococcus* but included also in this article are descriptions of the treatment of *Escherichia*, *Klebsiella*, and *Pseudomonas* infections. Infections are described as "long-term" and suppurative, etiologies as "drug-resistant", and patients as having (p. 35) "various histories of disease and antibiotic therapy". Phages were delivered orally as well as locally. They noted (p. 32), "In 44 cases a complete remission of the disease symptoms was achieved, accompanied by negative bacteriologic tests." Though we consider it possibly likely that these positive results were a consequence of phage action, still it is difficult to say from the information supplied that the results were not instead a consequence of antibiotic treatments, including hypothetical newly introduced antibiotic treatments.

8. Little or No Efficacy Observed

In this section we provide summaries of clinical phage-therapy studies that we have categorized in Table 1 as not having a Phage Impact Score (PIS). These are studies for which it is difficult to conclude that efficacy was observed, though in many cases these studies were not intended to demonstrate efficacy.

8.1. Dedrick et al., 2021, M. abscessus, lung

Dedrick et al. [50] treated an 81-year-old male intravenously with a phage cocktail, presumably via expanded access, for an *M. abscessus* infection which previous antibiotic treatment had failed to clear. The cocktail consisted of three phages; see Dedrick et al. [51] (main text) for cocktail details. Treatment was with 10^9 PFUs applied two times per day for six months. The patient was treated with numerous antibiotics overlapping with the phage therapy: azithromycin, bactrim, cefdinir, clofazimine, ethambutol, imipenem, and omadacycline. During the first month of phage treatment there was a ~1 log drop in sputum bacterial burden, with treatment with clofazimine and imipenem occurring also during this month. At least within the sputum, bacterial densities prior to the start of phage treatment would not be expected to be sufficient to support substantial phage population growth, e.g., about 250 CFU/ml, and dosages of 10^9 PFUs would not be expected to reach those bacteria in sufficient numbers to result in inundation without substantial phage replication or otherwise substantial concentration in the lungs.

Sputum CFUs were then to rise substantially (multiple logs) starting at 3 months following the initiation of phage treatment. This rise was coincident with discontinuation of clofazimine treatment, which had been ongoing for ~20 months, though authors suggest that the patient's humoral immune response could have prevented further successful phage impact after that first month. Overall, we feel that evidence is not strong that phage therapy had a substantial impact on what reductions in bacterial numbers were seen, con-

sisting of one datum in association with clofazimine treatment that, upon clofazimine discontinuation, was associated with substantial increases in target-bacterium numbers. An argument against that conclusion, however, is that resistance to one of the phages used was detected starting two months after the start of phage treatment.

8.2. Grubb et al., 2020, E. coli, gastrointestinal tract health trial

Grubb et al. [52] is equivalent to the study by Febvre et al. [53] (above) except that it was done in association with the probiotic, Bifidobacterium animalis subsp. lactis (23 placebo, 24 probiotic only, 23 probiotic plus phages). Although differences such as in gastric function and stool consistency were reported between phage treatments with and without *B. animalis*, no impact on *E. coli* densities is indicated.

8.3. Gilbey et al., 2019, S. aureus, sepsis

Gilbey et al. [54] treated via special access a 65-year-old male with a *S. aureus* sepsis that was associated with a prosthetic heart valve-centered endocarditis. A three-phage cocktail, AB-SA01, was produced using good manufacturing practices and supplied by AmpliPhi. It contained 10^9 PFU of each phage per dose, and was delivered intravenously two times per day for two weeks. Flucloxacillin treatment began 9 days prior to the start of phage treatment, with the dosage increased 4 days after the start of phage treatment. Ciprofloxacin treatment began 7 days prior to phage treatment, and rifampicin treatment began 5 days prior to phage treatment. Noticeable drops in patient temperature, white cell counts, and C-reactive protein seem to have occurred especially between days 4 and 6 after the start of phage treatment, though with a 1°C drop in body temperature also occurring immediately following phage treatment. The authors note, though (p. 142), that "Blood cultures were negative at onset of bacteriophage therapy", as is indicated 3 days prior to the start of phage therapy. Given that the infection appears to have been cured prior to the start of phage treatment, it is difficult to associate efficacy effects with phage delivery.

8.4. Gindin et al., 2019, E. coli, oral safety trial

The study by Gindin et al. [55] is equivalent to that of Febvre et al. [53] (discussed above) except that it was limited to determination of phage safety.

8.5. McCallin et al., 2018, S. aureus, oral safety trial

McCallin et al. [7] performed a phase I safety study involving 21 females. Each patient was dosed twice orally with each of placebo, a single anti-*Staphylococcus* phage, and Pyophage cocktail (supplied by Eliava Institute), supplied in a randomized order. This was done on days 1, 8, and 15 with 10 women dosed orally and 11 dosed nasally. No determinations of impact on targeted bacteria were made.

8.6. Sarker et al., 2017, E. coli, phase I trial

Sarker et al. [56] present a phase I, placebo-controlled randomized trial employing an oral dose of an anti-*E. coli* phage cocktail. As this was a safety trial there was no efficacy component, with no efficacy results noted. See also Sarker et al. [57]

8.7. Sarker et al., 2016, E. coli-associated diarrhea

Sarker et al. [57] treated diarrhea in children positive for pathogenic *E. coli*. Phages were supplied orally but no resulting efficacy was observed relative to the placebo control group. This lack of efficacy may have been due to (i) a lack of phage impact on the *E. coli* present, (ii) something in addition to *E. coli* causing the diarrhea, or (iii) insufficient phage numbers supplied.

8.8. Łusiak-Szelachowska et al., 2014, various etiologies, various infection types

Łusiak-Szelachowska et al. [58] is primarily an analysis of human serum for neutralizing antibodies given phage treatment. Insufficient detail is provided to reach a conclusion about the role of phages in anti-infection efficacy. They do note, however, (p. 303) that "Our preliminary observations performed in 15 patients with high antiphage activity of sera suggest that the induction of antiphage activity of patients' sera during or after

[phage therapy] does not exclude a good result of [phage therapy].” See Łusiak-Szelachowska et al. [10] for extension of this study.

8.9. Rose et al., 2014, P. aeruginosa and S. aureus, burn wound

Rose et al. [59] reported on a small (nine adults) safety rather than efficacy, burn-wound treatment trial. Bacterial loads were mostly initially quite low and otherwise unchanged following treatments. The study is briefly discussed in Abedon [47].

8.10. McCallin et al., 2013, various etiologies, oral safety trial

McCallin et al. [60] present a small, oral phage safety trial consisting of ten children and five adults. They consumed a commercially available phage cocktail, ColiProteus, as sold by the Russian company, Microgen. No infection treatment was attempted though fecal microbiota was analyzed for potential modifications, though with no definite conclusions.

8.11. Sarker et al., 2012, E. coli, oral safety trial

Sarker et al. [61] present a small, oral phage safety trial using a nine-phage cocktail. Fifteen healthy adults were treated orally. Fecal microbiota was analyzed with no changes detected.

8.12. Rhoads et al., 2009, various etiologies, venous leg ulcers

Rhoads et al. [62] in a phase I, randomized, double-blind clinical trial, treated *P. aeruginosa*- or *S. aureus*-infected venous leg ulcers using a topically applied 8-phage cocktail (WPP-201) that was provided by Intralytix. No significant anti-infection effectiveness was reported, but this was a safety- rather than efficacy-designed study. See Kutter et al. [63] for detailed discussion. This study is also discussed in Abedon [39,41,47].

8.13. Bruttin and Brüssow, 2005, E. coli, oral safety trial

Bruttin and Brüssow [64] present a safety trial using oral doses of coliphage T4. There were no declines in fecal *E. coli* concentrations.

8.14. Weber-Dąbrowska et al, 2002, various etiologies, various infection types

Weber-Dąbrowska et al. [65] looked at the impact of phage therapy on neutrophil activity, with no efficacy results reported.

References

1. Doub, J. B.; Ng, V. Y.; Wilson, E.; Corsini, L.; Chan, B. K. Successful treatment of a recalcitrant *Staphylococcus epidermidis* prosthetic knee infection with intraoperative bacteriophage therapy. *Pharmaceuticals* **2021,** *14*.
2. Rohde, C.; Resch, G.; Pirnay, J. P.; Blasdel, B. G.; Debarbieux, L.; Gelman, D.; Górski, A.; Hazan, R.; Huys, I.; Kakabadze, E.; et al. Expert opinion on three phage therapy related topics: bacterial phage resistance, phage training and prophages in bacterial production strains. *Viruses* **2018,** *10,* 178.
3. Ferry, T.; Kolenda, C.; Batailler, C.; Gaillard, R.; Gustave, C. A.; Lustig, S.; Fevre, C.; Petitjean, C.; Leboucher, G.; Laurent, F. Case report: Arthroscopic "Debridement Antibiotics and Implant Retention" with local injection of personalized phage therapy to salvage a relapsing *Pseudomonas aeruginosa* prosthetic knee infection. *Front Med. (Lausanne)* **2021,** *8*, 569159.
4. Lebeaux, D.; Merabishvili, M.; Caudron, E.; Lannoy, D.; Van Simaey, L.; Duyvejonck, H.; Guillemain, R.; Thumerelle, C.; Podglajen, I.; Compain, F.; et al. A case of phage therapy against pandrug-resistant *Achromobacter xylosoxidans* in a 12-year-old lung-transplanted cystic fibrosis patient. *Viruses* **2021,** *13*.
5. Leitner, L.; Ujmajuridze, A.; Chanishvili, N.; Goderdzishvili, M.; Chkonia, I.; Rigvava, S.; Chkhotua, A.; Changashvili, G.; McCallin, S.; Schneider, M. P.; et al. Intravesical bacteriophages for treating urinary tract infections in patients undergoing transurethral resection of the prostate: a randomised, placebo-controlled, double-blind clinical trial. *Lancet Infect. Dis.* **2021,** *21*, 427-436.

6. Leitner, L.; Sybesma, W.; Chanishvili, N.; Goderdzishvili, M.; Chkhotua, A.; Ujmajuridze, A.; Schneider, M. P.; Sartori, A.; Mehnert, U.; Bachmann, L. M.; et al. Bacteriophages for treating urinary tract infections in patients undergoing transurethral resection of the prostate: a randomized, placebo-controlled, double-blind clinical trial. *BMC. Urol.* **2017,** *17*, 90.

7. McCallin, S.; Sarker, S. A.; Sultana, S.; Oechslin, F.; Brussow, H. Metagenome analysis of Russian and Georgian Pyophage cocktails and a placebo-controlled safety trial of single phage versus phage cocktail in healthy *Staphylococcus aureus* carriers. *Environ. Microbiol.* **2018,** *20*, 3278-3293.

8. Kutateladze, M.; Adamia, R. Phage therapy experience at the Eliava Institute. *Med. Mal. Infect.* **2008,** *38*, 426-430.

9. Danis-Wlodarczyk, K.; Dabrowska, K.; Abedon, S. T. Phage therapy: the pharmacology of antibacterial viruses. *Curr. Issues Mol. Biol.* **2021,** *40*, 81-164.

10. Lusiak-Szelachowska, M.; Miedzybrodzki, R.; Fortuna, W.; Borysowski, J.; Górski, A. Anti-phage serum antibody responses and the outcome of phage therapy. *Folia Microbiol.* **2021,** *66*, 127-131.

11. Ramirez-Sanchez, C.; Gonzales, F.; Buckley, M.; Biswas, B.; Henry, M.; Deschenes, M. V.; Horne, B.; Fackler, J.; Brownstein, M. J.; Schooley, R. T.; et al. Successful treatment of *Staphylococcus aureus* prosthetic joint infection with bacteriophage therapy. *Viruses* **2021,** *13*.

12. Rostkowska, O. M.; Miedzybrodzki, R.; Miszewska-Szyszkowska, D.; Górski, A.; Durlik, M. Treatment of recurrent urinary tract infections in a 60-year-old kidney transplant recipient. The use of phage therapy. *Transpl. Infect. Dis.* **2021,** *23*, e13391.

13. Tan, X.; Chen, H.; Zhang, M.; Zhao, Y.; Jiang, Y.; Liu, X.; Huang, W.; Ma, Y. Clinical experience of personalized phage therapy against carbapenem-resistant *Acinetobacter baumannii* lung infection in a patient with chronic obstructive pulmonary disease. *Front Cell Infect. Microbiol.* **2021,** *11*, 631585.

14. Aslam, S.; Lampley, E.; Wooten, D.; Karris, M.; Benson, C.; Strathdee, S.; Schooley, R. T. Lessons learned from the first 10 consecutive cases of intravenous bacteriophage therapy to treat multidrug-resistant bacterial infections at a single center in the United States. *Open Forum Infect. Dis.* **2020,** *7*, ofaa389.

15. Schooley, R. T.; Biswas, B.; Gill, J. J.; Hernandez-Morales, A.; Lancaster, J.; Lessor, L.; Barr, J. J.; Reed, S. L.; Rohwer, F.; Benler, S.; et al. Development and use of personalized bacteriophage-based therapeutic cocktails to treat a patient with a disseminated resistant *Acinetobacter baumannii* infection. *Antimicrob. Agents Chemother.* **2017,** *61*, e00954-17.

16. LaVergne, S.; Hamilton, T.; Biswas, B.; Kumaraswamy, M.; Schooley, R. T.; Wooten, D. Phage therapy for a multidrug-resistant *Acinetobacter baumannii* craniectomy site infection. *Open Forum Infect. Dis.* **2018,** *5*, ofy064.

17. Aslam, S.; Courtwright, A. M.; Koval, C.; Lehman, S. M.; Morales, S.; Furr, C.-L. L.; Rosas, F.; Brownstein, M. J.; Fackler, J. R.; Sisson, B. M.; et al. Early clinical experience of bacteriophage therapy in three lung transplant recipients. *Am. J Transplant.* **2019,** *19*, 2631-2639.

18. Aslam, S.; Pretorius, V.; Lehman, S. M.; Morales, S.; Schooley, R. T. Novel bacteriophage therapy for treatment of left ventricular assist device infection. *J Heart Lung Transplant.* **2019,** *38*, 475-476.

19. Law, N.; Logan, C.; Yung, G.; Furr, C. L. L.; Lehman, S. M.; Morales, S.; Rosas, F.; Gaidamaka, A.; Bilinsky, I.; Grint, P. Successful adjunctive use of bacteriophage therapy for treatment of multidrug-resistant *Pseudomonas aeruginosa* infection in a cystic fibrosis patient. *Infection* **2019,** *47*, 665-668.

20. Doub, J. B.; Ng, V. Y.; Johnson, A. J.; Slomka, M.; Fackler, J.; Horne, B.; Brownstein, M. J.; Henry, M.; Malagon, F.; Biswas, B. Salvage bacteriophage therapy for a chronic MRSA prosthetic joint infection. *Antibiotics (Basel)* **2020,** *9*.

21. Ferry, T.; Batailler, C.; Petitjean, C.; Chateau, J.; Fevre, C.; Forestier, E.; Brosset, S.; Leboucher, G.; Kolenda, C.; Laurent, F.; et al. The potential innovative use of bacteriophages within the DAC((R)) hydrogel to treat patients with knee megaprosthesis infection requiring "Debridement antibiotics and implant retention" and soft tissue coverage as salvage therapy. *Front Med. (Lausanne)* **2020,** *7*, 342.

22. Ferry, T.; Kolenda, C.; Batailler, C.; Gustave, C. A.; Lustig, S.; Malatray, M.; Fevre, C.; Josse, J.; Petitjean, C.; Chidiac, C.; et al. Phage therapy as adjuvant to conservative surgery and antibiotics to salvage patients with relapsing *S. aureus* prosthetic knee infection. *Front Med. (Lausanne)* **2020,** *7*, 570572.

23. Gainey, A. B.; Burch, A. K.; Brownstein, M. J.; Brown, D. E.; Fackler, J.; Horne, B.; Biswas, B.; Bivens, B. N.; Malagon, F.; Daniels, R. Combining bacteriophages with cefiderocol and meropenem/vaborbactam to treat a pan-drug resistant *Achromobacter* species infection in a pediatric cystic fibrosis patient. *Pediatr. Pulmonol.* **2020,** *55*, 2990-2994.

24. Petrovic Fabijan, P.; .; Lin, R. C. Y.; Ho, J.; Maddocks, S.; Ben Zakour, N. L.; Iredell, J. R.; Westmead Bacteriophage Therapy Team Safety of bacteriophage therapy in severe *Staphylococcus aureus* infection. *Nat. Microbiol.* **2020,** *5*, 465-472.

25. Abedon, S. T. Active bacteriophage biocontrol and therapy on sub-millimeter scales towards removal of unwanted bacteria from foods and microbiomes. *AIMS Microbiol.* **2017,** *3*, 649-688.

26. Qin, J.; Wu, N.; Bao, J.; Shi, X.; Ou, H.; Ye, S.; Zhao, W.; Wei, Z.; Cai, J.; Li, L.; et al. Heterogeneous *Klebsiella pneumoniae* co-infections complicate personalized bacteriophage therapy. *Front Cell Infect. Microbiol.* **2020,** *10*, 608402.

27. Gupta, P.; Singh, H. S.; Shukla, V. K.; Nath, G.; Bhartiya, S. K. Bacteriophage therapy of chronic nonhealing wound: clinical study. *Int. J Low Extrem. Wounds.* **2019,** *18*, 171-175.

28. Onsea, J.; Soentjens, P.; Djebara, S.; Merabishvili, M.; Depypere, M.; Spriet, I.; De, M. P.; Debaveye, Y.; Nijs, S.; Vanderschot, P.; et al. Bacteriophage application for difficult-to-treat musculoskeletal infections: development of a standardized multidisciplinary treatment protocol. *Viruses* **2019,** *11*.

29. Tkhilaishvili, T.; Winkler, T.; Muller, M.; Perka, C.; Trampuz, A. Bacteriophages as adjuvant to antibiotics for the treatment of periprosthetic joint infection caused by multidrug-resistant *Pseudomonas aeruginosa*. *Antimicrob. Agents Chemother.* **2019,** *64*, e00924-19.

30. Brown-Jaque, M.; Muniesa, M.; Navarro, F. Bacteriophages in clinical samples can interfere with microbiological diagnostic tools. *Sci. Rep.* **2016,** *6*, 33000.

31. Chibeu, A.; Balamurugan, S. Application of a virucidal agent to avoid overestimation of phage kill during phage decontamination assays on ready-to-eat meats. *Meth. Mol. Biol.* **2018,** *1681*, 97-105.

32. Duplessis, C.; Biswas, B.; Hanisch, B.; Perkins, M.; Henry, M.; Quinones, J.; Wolfe, D.; Estrella, L.; Hamilton, T. Refractory *Pseudomonas* bacteremia in a 2-year-old sterilized by bacteriophage therapy. *J. Pediatric Infect. Dis. Soc.* **2018,** *7*, 253-256.

33. Ferry, T.; Boucher, F.; Fevre, C.; Perpoint, T.; Chateau, J.; Petitjean, C.; Josse, J.; Chidiac, C.; L'Hostis, G.; Leboucher, G.; et al. Innovations for the treatment of a complex bone and joint infection due to XDR *Pseudomonas aeruginosa* including local application of a selected cocktail of bacteriophages. *J Antimicrob. Chemother.* **2018,** *73*, 2901-2903.

34. Ferry, T.; Leboucher, G.; Fevre, C.; Herry, Y.; Conrad, A.; Josse, J.; Batailler, C.; Chidiac, C.; Medina, M.; Lustig, S.; et al. Salvage debridement, antibiotics and implant retention ("DAIR") with local injection of a selected cocktail of bacteriophages: is it an option for an elderly patient with relapsing *Staphylococcus aureus* prosthetic-joint infection? *Open Forum Infect. Dis.* **2018,** *5*, ofy269.

35. Fish, R.; Kutter, E.; Bryan, D.; Wheat, G.; Kuhl, S. Resolving digital staphylococcal osteomyelitis using bacteriophage-a case report. *Antibiotics (Basel)* **2018,** *7*.

36. Fish, R.; Kutter, E.; Wheat, G.; Blasdel, B.; Kutateladze, M.; Kuhl, S. Bacteriophage treatment of intransigent diabetic toe ulcers: a case series. *J. Wound Care* **2016,** *25 Suppl 7*, S27-S33.

37. Patey, O.; McCallin, S.; Mazure, H.; Liddle, M.; Smithyman, A.; Dublanchet, A. Clinical indications and compassionate use of phage therapy: personal experience and literature review with a focus on osteoarticular infections. *Viruses.* **2018,** *11*.

38. Ujmajuridze, A.; Chanishvili, N.; Goderdzishvili, M.; Leitner, L.; Mehnert, U.; Chkhotua, A.; Kessler, T. M.; Sybesma, W. Adapted bacteriophages for treating urinary tract infections. *Front. Microbiol.* **2018,** *9*, 1832.

39. Abedon, S. T. Use of phage therapy to treat long-standing, persistent, or chronic bacterial infections. *Advanced Drug Delivery Reviews* **2019,** *145*, 18-39.

40. Kutateladze, M. Experience of the Eliava Institute in bacteriophage therapy. *Virol. Sin.* **2015,** *30*, 80-81.

41. Abedon, S. T. Bacteriophage clinical use as antibactertial "drugs": utility, precedent. *Microbiol. Spectr.* **2017,** *5*, BAD-0003-2016.

42. Khawaldeh, A.; Morales, S.; Dillon, B.; Alavidze, Z.; Ginn, A. N.; Thomas, L.; Chapman, S. J.; Dublanchet, A.; Smithyman, A.; Iredell, J. R. Bacteriophage therapy for refractory *Pseudomonas aeruginosa* urinary tract infection. *J Med. Microbiol.* **2011,** *60*, 1697-1700.

43. Marza, J. A. S.; Soothill, J. S.; Boydell, P.; Collyns, T. A. Multiplication of therapeutically administered bacteriophages in *Pseudomonas aeruginosa* infected patients. *Burns* **2006,** *32*, 644-646.

44. Weber-Dabrowska, B.; Zimecki, M.; Kruzel, M.; Kochanowska, I.; Lusiak-Szelachowska, M. Alternative therapies in antibiotic-resistant infection. *Adv. Med. Sci.* **2006,** *51*, 242-244.

45. Markoishvili, K.; Tsitlanadze, G.; Katsarava, R.; Morris, J. G., Jr.; Sulakvelidze, A. A novel sustained-release matrix based on biodegradable poly(ester amide)s and impregnated with bacteriophages and an antibiotic shows promise in management of infected venous stasis ulcers and other poorly healing wounds. *Int. J. Dermatol.* **2002,** *41*, 453-458.

46. Jikia, D.; Chkhaidze, N.; Imedashvili, E.; Mgaloblishvili, I.; Tsitlanadze, G.; Katsarava, R.; Glenn Morris, J. J.; Sulakvelidze, A. The use of a novel biodegradable preparation capable of the sustained release of bacteriophages and ciprofloxacin, in the complex treatment of multidrug-resistant *Staphylococcus aureus*-infected local radiation injuries caused by exposure to Sr^{90}. *Clin. Exp. Dermatol.* **2005,** *30*, 23-26.

47. Abedon, S. T. Bacteriophage-mediated biocontrol of wound infections, and ecological exploitation of biofilms by phages. In *Biofilm, Pilonidal Cysts and Sinuses. Recent Clinical Techniques, Results, and Research in Wounds, vol 1,* Shiffman, M.; Low, M., Eds.; Springer Nature: **2020**; 121-158.

48. Weber-Dabrowska, B.; Mulczyk, M.; Górski, A. Bacteriophage therapy for infections in cancer patients. *Clin. Appl. Immunol. Rev.* **2001,** *1*, 131-134.

49. Weber-Dabrowska, B.; Zimecki, M.; Mulczyk, M. Effective phage therapy is associated with normalization of cytokine production by blood cell cultures. *Arch. Immunol. Ther. Exp.* **2000,** *48*, 31-37.

50. Dedrick, R. M.; Freeman, K. G.; Nguyen, J. A.; Bahadirli-Talbott, A.; Smith, B. E.; Wu, A. E.; Ong, A. S.; Lin, C. T.; Ruppel, L. C.; Parrish, N. M.; et al. Potent antibody-mediated neutralization limits bacteriophage treatment of a pulmonary *Mycobacterium abscessus* infection. *Nat. Med.* **2021,** *27*, 1357-1361.

51. Dedrick, R. M.; Guerrero-Bustamante, C. A.; Garlena, R. A.; Russell, D. A.; Ford, K.; Harris, K.; Gilmour, K. C.; Soothill, J.; Jacobs-Sera, D.; Schooley, R. T.; et al. Engineered bacteriophages for treatment of a patient with a disseminated drug-resistant *Mycobacterium abscessus*. *Nat. Med.* **2019,** *25*, 730-733.

52. Grubb, D. S.; Wrigley, S. D.; Freedman, K. E.; Wei, Y.; Vazquez, A. R.; Trotter, R. E.; Wallace, T. C.; Johnson, S. A.; Weir, T. L. PHAGE-2 study: supplemental bacteriophages extend *Bifidobacterium animalis* subsp. *lactis* BL04 benefits on gut health and microbiota in healthy adults. *Nutrients.* **2020,** *12*.

53. Febvre, H. P.; Rao, S.; Gindin, M.; Goodwin, N. D. M.; Finer, E.; Vivanco, J. S.; Lu, S.; Manter, D. K.; Wallace, T. C.; Weir, T. L. PHAGE study: effects of supplemental bacteriophage intake on inflammation and gut microbiota in healthy adults. *Nutrients.* **2019,** *11*.

54. Gilbey, T.; Ho, J.; Cooley, L. A.; Petrovic, F. A.; Iredell, J. R. Adjunctive bacteriophage therapy for prosthetic valve endocarditis due to *Staphylococcus aureus*. *Med. J Aust.* **2019,** *211*, 142-143.

55. Gindin, M.; Febvre, H. P.; Rao, S.; Wallace, T. C.; Weir, T. L. Bacteriophage for gastrointestinal health (PHAGE) study: evaluating the safety and tolerability of supplemental bacteriophage consumption. *J. Am. Coll. Nutr.* **2019,** *38*, 68-75.

56. Sarker, S. A.; Berger, B.; Deng, Y.; Kieser, S.; Foata, F.; Moine, D.; Descombes, P.; Sultana, S.; Huq, S.; Bardhan, P. K.; et al. Oral application of *Escherichia coli* bacteriophage: safety tests in healthy and diarrheal children from Bangladesh. *Environ. Microbiol.* **2017,** *19*, 237-250.

57. Sarker, S. A.; Sultana, S.; Reuteler, G.; Moine, D.; Descombes, P.; Charton, F.; Bourdin, G.; McCallin, S.; Ngom-Bru, C.; Neville, T.; et al. Oral phage therapy of acute bacterial diarrhea with two coliphage preparations: a randomized trial in children from Bangladesh. *EBioMedicine* **2016,** *4*, 124-137.

58. Lusiak-Szelachowska, M.; Zaczek, M.; Weber-Dabrowska, B.; Miedzybrodzki, R.; Klak, M.; Fortuna, W.; Letkiewicz, S.; Rogoz, P.; Szufnarowski, K.; Jonczyk-Matysiak, E.; et al. Phage neutralization by sera of patients receiving phage therapy. *Viral Immunol.* **2014,** *27*, 295-304.

59. Rose, T.; Verbeken, G.; Vos, D. D.; Merabishvili, M.; Vaneechoutte, M.; Lavigne, R.; Jennes, S.; Zizi, M.; Pirnay, J. P. Experimental phage therapy of burn wound infection: difficult first steps. *Int. J. Burns Trauma* **2014,** *4*, 66-73.

60. McCallin, S.; Alam, S. S.; Barretto, C.; Sultana, S.; Berger, B.; Huq, S.; Krause, L.; Bibiloni, R.; Schmitt, B.; Reuteler, G.; et al. Safety analysis of a Russian phage cocktail: from metaGenomic analysis to oral application in healthy human subjects. *Virology* **2013,** *443*, 187-196.

61. Sarker, S. A.; McCallin, S.; Barretto, C.; Berger, B.; Pittet, A. C.; Sultana, S.; Krause, L.; Huq, S.; Bibiloni, R.; Bruttin, A.; et al. Oral T4-like phage cocktail application to healthy adult volunteers from Bangladesh. *Virology* **2012,** *434*, 222-232.

62. Rhoads, D. D.; Wolcott, R. D.; Kuskowski, M. A.; Wolcott, B. M.; Ward, L. S.; Sulakvelidze, A. Bacteriophage therapy of venous leg ulcers in humans: results of a phase I safety trial. *J. Wound Care* **2009,** *18*, 237-244.

63. Kutter, E.; De Vos, D.; Gvasalia, G.; Alavidze, Z.; Gogokhia, L.; Kuhl, S.; Abedon, S. T. Phage therapy in clinical practice: treatment of human infections. *Curr. Pharm. Biotechnol.* **2010,** *11*, 69-86.

64. Bruttin, A.; Brüssow, H. Human volunteers receiving *Escherichia coli* phage T4 orally: a safety test of phage therapy. *Antimicrob. Agents Chemother.* **2005,** *49*, 2874-2878.

65. Weber-Dabrowska, B.; Zimecki, M.; Mulczyk, M.; Górski, A. Effect of phage therapy on the turnover and function of peripheral neutrophils. *FEMS Immunol. Med. Microbiol.* **2002,** *34*, 135-138.
