# Supplementary material for: Phage Therapy in the 21st Century: Is There Modern, Clinical Evidence of Phage-Mediated Efficacy?
Source: Pharmaceuticals (Basel). 2021 Nov 13;14(11):1157. doi: 10.3390/ph14111157 (PMC8625828; doi:10.3390/ph14111157)
Supplement: Supplementary file 1 [file pharmaceuticals-14-01157-s001.zip › pharmaceuticals-1439557-supplementary/Supplementary Files/Table S1 (002, as finalized).pdf]

# **Table S1.** Summary of clinical phage therapy studies, 2000 to present.[a]

| Authors | Year | Targeted Etiologies[b] | Infection Type(s) | Number Treated | Efficacy Observed?[c] | Phage Impact Score[d] | Cocktail?[e] | # Phage Types | Titer (PFUs/ml)[f] | Dosage (PFUs)[g] | Volume (ml) | Dosing Frequency (hours)[h] | Number of Doses | Dosing Duration (days) | Route | Exotoxin Units (EUs)/ml | Antibiotic Co-treatments?[i] |
|---|---|---|---|---|---|---|---|---|---|---|---|---|---|---|---|---|---|

[The table proper can be found starting on the following page]

[a] **Abbreviations:** IA, intraarticular; IN, intranasal (including intrasinus); IP, intraperitoneal; IR, intrarectal; ISb, instillation into the bladder; IV, intravenous; Loc, local application; Neb, nebulized (to lungs); NR, not removed; PO, per os (i.e., oral delivery); POn, per os with acid neutralization; U, undetectable; UI, urethral instillation.

[b] **Abbreviations:** *Ab, Acinetobacter baumannii*; *Ac~, Achromobacter* spp.; *Ax, Achromobacter xylosoxidans*; *Bd, Burkholderia dolosa*; *Ci~, Citrobacter* spp.; *Eb~, Enterobacter* spp.; *Ec, Escherichia coli*; *Efm, Enterococcus faecium*; *Efs, Enterococcus faecalis*; *En~, Enterococcus* spp.; *Es~, Escherichia* spp.; *Enc, Enterobacter cloacae*; Gn, Gram negative; *Kl~, Klebsiella* spp.; *Ko, Klebsiella oxytoca*; *Kp, Klebsiella pneumoniae*; *Ma, Mycobacterium abscessus*; *Mm, Morganella morganii*; *Pa, Pseudomonas aeruginosa*; *Pm, Proteus mirabilis*; *Pr~, Proteus* spp.; *Ps~, Pseudomonas* spp.; *Sa, Staphylococcus aureus*; *Se, Staphylococcus epidermidis*; Sha, *Staphylococcus haemolyticus*; *Sho, Staphylococcus homis*; *Sl~, Salmonella* spp.; *Sme, Serratia marcescens*; *Smi, Streptococcus mitis*; *Sn, Stenotrophomonas* spp.; *So~, Streptococcus* spp.; *Ssi, Staphylococcus simularans*; *Sta, Streptococcus agalactiae*; *Ssa, Staphylococcus saprophyticus*; *Sy~, Staphylococcus* spp.

[c] "Efficacy observed" is as following phage treatment, whether or not microbiological or clinical improvement was observed, though whether or not efficacy was a consequence specifically of phage action is not addressed in this column.

[d] A "2" indicates that in our opinion there is reasonably strong evidence provided in the cited article to conclude that substantial anti-bacterial infection activity was phage mediated. A "1" indicates evidence that efficacy was phage mediated though either evidence or likely phage impact is not as substantial as for a score of "2:. A score of "0" indicates that we have not been convinced by the cited article that there is evidence that efficacy was phage mediated. The latter typically is because antibiotics to which the targeted bacterium is sensitive were concurrently applied without evidence provided of previous failures by the same antibiotics to treat the infection in question. This, though, should not be viewed as a judgment on the quality of a publication. A "0.5" indicates that we were uncertain whether to assign a value of "0" vs. "1". "NA" is used if efficacy is not observed. "M" means that microbiological evidence (reduction in numbers of bacteria) was used as an indicator of success while "C" indicates that the indicator was clinical, i.e., patient clinical improvement. We indicate only "M" if both "M" and "C" were the seen. As a caveat, note that any individual example of successful infection treatment could in principle have been a consequence of spontaneous resolution, and it is likely that many unsuccessful treatments have not been published. Therefore, these ranking cannot be used as substitutes for successful randomized, double-blinded efficacy trials.

[e] "Yes" if a cocktail was used for at least one case in a study. Cocktails consist of phage treatments consisting of at least two phage types.

[f] Plaque-forming units (PFUs)/ml units. Values calculated by us are shown in parentheses. "—" = information was not found or is difficult to interpret. "~" = too much information is presented in a study to summarize here.

[g] Total PFUs applied.

[h] Doses are presented either in reported hour intervals or as calculated based on 24/number of doses. Thus, for example, 8 hours would imply either actual treatment every 8 hours or instead treatment stated as occurring three times per day.

[i] "Yes" is indicated if there is any overlap between a phage treatment course and antibiotic treatment course, though the study could still contain treatments without antibiotic, as discussed in the main text.

| Authors | Year | Targeted Etiologies | Infection Type(s) | Number Treated | Efficacy Observed? | Phage Impact Score | Cocktail? | # Phage Types | Titer (PFUs/ml) | Dosage (PFUs) | Volume (ml) | Dosing Frequency (hours) | Number of Doses | Dosing Duration (days) | Route | Exotoxin Units (EUs)/ml | Antibiotic Co-treatments? |
|---|---|---|---|---|---|---|---|---|---|---|---|---|---|---|---|---|---|
| Cano et al. [1] | 2021 | *Kp* | Prosthetic joint | 1 | Yes | 1 (C) | No | 1 | ($1.3 \times 10^{9}$) | $6.3 \times 10^{10}$ | 50 | 24[j] | 40 | 56 | IV | 22 | Yes |
| Dedrick et al. [2] | 2021 | *Ma* | Lung | | No | NA | Yes | 3 | — | $1 \times 10^{9}$ | — | 12 | (~360) | ~180 | IV | U | Yes |
| Doub et al. [3] | 2021 | *Se* | Prosthetic joint | 1 | Yes | 0 | No | 1 | $2 \times 10^{9}$ | ($2 \times 10^{10}$) | 10 | — | 1 | — | IA | (<10) | Yes |
| Ferry et al. [4] | 2021 | *Pa* | Prosthetic joint | 1 | Yes | 0 | Yes | 3 | $3 \times 10^{9}$ | ($9 \times 10^{10}$) | 30 | — | 1 | — | IA | — | Yes |
| Johri et al. [5] | 2021 | *Efs, Sa, Sha, Smi* | Chronic prostatitis | 1 | Yes | 2 (M) | Yes | — | — | — | — | — | — | (~300) | IR, PO, UI | — | No |
| Lebeaux et al. [6] | 2021 | *Ax* | Lung | 1 | Yes | 0 | Yes | 3 to 4 | [k]$4 \times 10^{9}$; $5 \times 10^{8}$; $5 \times 10^{8}$ | ($2 \times 10^{10}$); ($1.5 \times 10^{10}$); ($2.5 \times 10^{9}$) | 5; 30; 5 | 8; 24; 8 | (6); 1; (42) | 2; —; 14 | Neb; Loc[l]; Neb | 176 | Yes |
| Leitner et al. [7] | 2021 | *En~, Ec, Pm, Pa, Sy~, So~* | Urinary tract | 1 | Yes | 0 | Yes | Pyo[m] | $1 \times 10^{4}$ to $\geq 1 \times 10^{5}$ | ($2 \times 10^{5}$) to ($\geq 2 \times 10^{6}$) | 20 | 12 | (14) | 7 | ISb | 0.5 | No |
| Łusiak-Szelachowska et al. [8] | 2021 | *Ec, Kp, Pa, Sa* | Rhinosinusitis | 25 | Yes | 0 | Yes | — | — | — | 4, 6, or 15 (× 2) | — | — | — | IN, POn | — | — |
| Ramirez-Sanchez et al. [9] | 2021 | *Sa* | Prosthetic joint | 1 | Yes | 0.5 | Yes | 3 | ($1.9 \times 10^{9}$) or $2.9 \times 10^{10}$ | — | — | 12 | (28) or (84) | 14 or 42 | IA, IV | <250 | Yes |
| Rostkowska et al. [10] | 2021 | *Kp* | Urinary tract | 1 | Yes | 0 | — | — | — | — | 10 | 12 | (58) | 29 | IR | — | Yes |
| Tan et al. [11] | 2021 | *Ab* | Lung | 1 | Yes | 0 | No | 1 | $1 \times 10^{6}$ to $1 \times 10^{10}$ | — | — | 12 or 24 | (30) | 16 | Neb | $10^{4}$ to $10^{5}$ | Yes |
| Wu et al. [12] | 2021 | *Ab* | Lung | 4 | Yes | 1 (M) | Yes | 1 or 2 | $1 \times 10^{8}$ | $1 \times 10^{9}$ | 10 | 12 | 2 | 1 | Neb, Loc | — | Yes |
| Aslam et al. [13] | 2020 | *Ma, Pa, Sa* | Various | 10 | Yes | 0 | Yes | 1 to 5 | $2 \times 10^{5}$ to $3 \times 10^{10}$ to $1 \times 10^{11}$ | — | — | 2, 8, or 12 | 1 to many | 8 to > 100 | IV | — | Yes |
| Bao et al. [14] | 2020 | *Kp* | Urinary tract | 1 | Yes | 2 (M) | Yes | 5, 5, 6 | ($2.5 \times 10^{9}$), —, — | ($1.25 \times 10^{11}$) , —, — | 50, —, — | 24, —, 24 | 5, —, 5 | 5, 5, 5 | ISb | — | Yes |
| Corbellino et al. [15] | 2020 | *Kp* | Gut decolonization | 1 | Yes | 1 (M) | No | 1 | $1 \times 10^{6}$ | $1 \times 10^{6}$ to $1 \times 10^{7}$ | 10 | 12 | (42) | 21 | IR, POn | — | Yes or no |
| Doub et al. [16] | 2020 | *Sa* | Prosthetic joint | 1 | Yes | 0 | No | 1 | ($5 \times 10^{8}$); ($6 \times 10^{7}$) | $5 \times 10^{9}$; $3 \times 10^{9}$ | 10; 50 | —; 24 | 1; 3 | —; 3 | IA; IV | <1 | Yes |
| Ferry et al. [17] | 2020 | *Sa* | Prosthetic joint | 1 | Yes | 0 | Yes | 2 | ($1.7 \times 10^{9}$) | $1.7 \times 10^{10}$ | 6 | — | 1 | — | Loc | — | Yes |
| Ferry et al. [18] | 2020 | *Sa* | Prosthetic joint | 3 | Yes | 0 | Yes | 3 | $1 \times 10^{9}$ | — | — | — | 1 | — | IA | — | Yes |
| Gainey et al. [19] | 2020 | *Ac~* | Lung (with cystic fibrosis) | 1 | Yes | 0.5 (M) | No | 1 | — | — | — | 24 | 14 | 14[n] | IV | — | No then Yes |

[j] Patient was dosed on weekdays only.

[k] Lists within entries separated by semicolons (";", but not by commas) refer to separate entries with orders that are consistent across individual rows, i.e., within a single row, one column's 'A; B; C' maps directly onto another column's 'A; B; C', e.g., in this row these are in terms of dosed titers, PFUs, volumes, frequencies, number of doses, durations, and routes.

[l] Injection into the lung.

[m] Pyo = Pyophage, a type of phage cocktail commercially available in former Soviet Republics.

[n] This was done twice, the first time without antibiotic, though following 2 weeks of antibiotic treatment, and the second time in association with antibiotic treatment.

| Authors | Year | Targeted Etiologies | Infection Type(s) | Number Treated | Efficacy Observed? | Phage Impact Score | Cocktail? | # Phage Types | Titer (PFUs/ml) | Dosage (PFUs) | Volume (ml) | Dosing Frequency (hours) | Number of Doses | Dosing Duration (days) | Route | Exotoxin Units (EUs)/ml | Antibiotic Co-treatments? |
|---|---|---|---|---|---|---|---|---|---|---|---|---|---|---|---|---|---|
| Grubb et al. [20] | 2020 | *Ec* | gastrointestinal tract health trial | 23 | No | NA | Yes | 4 | — | $5 \times 10^6$ | — | 24 | 28 | 28 | PO | — | No |
| Petrovic Fabijan et al. [21] | 2020 | *Sa* | Various | 13 | Yes | 0 | Yes | 3 | — | $3 \times 10^9$ | 50 to 100 | 12 | 5 to 28[o] | 14 | IV | — | Yes |
| Qin et al. [22] | 2020 | *Kp* | Urinary tract | 1 | Yes | 0 | Yes | 1 to 4 | $5 \times 10^8$ | $2.5 \times 10^{10}$ | 50 | 48 | (28) | $14 \times 4$ | ISb[p] | — | No then Yes |
| Rubalskii et al. [23] | 2020 | *Ec, Efm, Kp, Pa, Sa* | Cardiothoracic surgery | 8 | Yes | 1 (M) | Yes | 1 to 4 | $1 \times 10^8$ to $4 \times 10^{10}$[q] | ~ | 2 to 50 | ~ | ~ | 1 to 14 | Loc, Neb, PO | — | Yes |
| Aslam et al. [24] | 2019 | *Pa, Bd* | Lung transplant | 3 | Yes | 0 | Yes | 1 to 4 | $5.3 \times 10^6$ to $5 \times 10^9$ | $4 \times 10^9$ | — | 2 to 24 | —[r] | 38 to 84 | IV, Neb | 0.2 to 200 | Yes |
| Aslam et al. [25] | 2019 | *Sa* | Implanted device, osteomyelitis, bacteremia | 1 | Yes | 1 (M) | Yes | 3 | — | $3 \times 10^9$ | — | 12 | (56) | 28 | IV | — | Yes |
| Dedrick et al. [26] | 2019 | *Ma* | Disseminated infection | 1 | Yes | 1 (M) | Yes | 3 | — | $3 \times 10^9$ | — | 12 | (64) | 32 | IV | U | Yes |
| Febvre et al. [27] | 2019 | *Ec* | gastrointestinal tract health trial | 36 | Yes | 1 (M) | Yes | 4 | — | $5 \times 10^6$ | — | 24 | 28 | 28 | PO | — | No |
| Gilbey et al. [28] | 2019 | *Sa* | Sepsis | 1 | No | NA | Yes | 3 | — | $3 \times 10^9$ | — | 12 | (28) | 14 | IV | — | Yes |
| Gindin et al. [29] | | *Ec* | Oral safety trial | 36 | No | NA | Yes | 4 | — | $5 \times 10^6$ | — | 24 | 28 | 28 | PO | — | No |
| Gupta et al. [30] | 2019 | *Ec, Pa, Sa* | chronic wounds | 20 | Yes | 0 | Yes | 3 | $1 \times 10^9$ | — | $0.1/cm^2$ | 48 | 3 to 5 | (6 to 10) | Loc | — | — |
| Jault et al. [31] | 2019 | *Pa* | Burn wound | 12 | Yes | 2 (M) | Yes | 12 | $1 \times 10^1$ to $1 \times 10^2$ | — | $0.1/cm^2$ | 24 | 7 | 7 | Loc | — | Yes or no[s] |
| Kuipers et al. [32] | 2019 | *Kp* | Urinary tract | 1 | Yes | 1 (M) | — | — | — | — | — | 12, 24, 48 | — | 84 | ISb, PO | — | Yes |
| Law et al. [33] | 2019 | *Pa* | Lung (with cystic fibrosis) | 1 | Yes | 0 | No | 1 | $(8 \times 10^8)$ | $4 \times 10^9$ | 5 | 6 | (224) | 56 | IV | — | Yes |
| Maddocks et al. [34] | 2019 | *Pa* | Lung | 1 | Yes | 1 (M) | Yes | 4 | $(4 \times 10^9)$; $(4 \times 10^7)$ | $(1.6 \times 10^{10})$; $(4 \times 10^9)$ | 4, 101 | 12 | (14) | 7 | Neb; IV | — | Yes |
| Nir-Paz et al. [35] | 2019 | *Ab, Kp* | Osteomyelitis | 1 | Yes | 1 (M) | Yes | 2 | $5 \times 10^7$ | $(1 \times 10^8)$ | 2 | 8 | (36) | 5 + 7 | IV | 5 to 35 | Yes |
| Onsea et al. [36] | 2019 | *Efs, Pa, Sta, Sa, Se* | Osteomyelitis | 4 | Yes | 0 | Yes | 3; Pyo | $1 \times 10^7$; — | $(1 \times 10^8)$ to $(4 \times 10^8)$ | 10, 20, or 40[t] | 8 | (56) to (80) | 7 to 10 | Loc | —; NR | Yes |
| Ooi et al. [37] | 2019 | *Sa* | Rhinosinusitis | 9 | Yes | 1 (M) | Yes | 3 | $(1.2 \times 10^6)$; $(1.2 \times 10^6)$; $(1.2 \times 10^7)$ | $3 \times 10^8$; $3 \times 10^8$; $3 \times 10^9$ | 250[u] | 12 | (14); (28); (28) | 7; 14; 14 | IN | — | No |

[o] One patient appears to have been treated with phages twice, for 28 days each time.

[p] One course of phage treatments also included 10 ml delivery via the kidney.

[q] Uncertain if this is per phage within cocktails or for all phages present in combination.

[r] Total numbers of doses applied are difficult to parse for this study.

[s] As dependent on the discretion of the treating physician.

[t] In addition, a phage-soaked sponge was placed in contact with the infection.

[u] Rinses based on 240 ml of water combined with an undisclosed amount of salts and 1 ml of phages, which we have rounded to 250 ml.

| Authors | Year | Targeted Etiologies | Infection Type(s) | Number Treated | Efficacy Observed? | Phage Impact Score | Cocktail? | # Phage Types | Titer (PFUs/ml) | Dosage (PFUs) | Volume (ml) | Dosing Frequency (hours) | Number of Doses | Dosing Duration (days) | Route | Exotoxin Units (EUs)/ml | Antibiotic Co-treatments? |
|---|---|---|---|---|---|---|---|---|---|---|---|---|---|---|---|---|---|
| Rogóz et al. [38] | 2019 | *Sa*[v] | Orthopedic | 76 | Yes | [w] | Yes | [x]1; 3 | $1 \times 10^6$; $\geq 3 \times 10^5$ | $1 \times 10^7$; — | 10; — | 8, 12; 8 | (84), (56); — | 28; — | PO, Loc; Loc | — | Yes |
| Tkhilaishvili et al. [40] | 2019 | *Pa* | Prosthetic joint | 1 | Yes | 0 | No | 1[y] | —, $1 \times 10^8$ | —; ($5 \times 10^8$) | 100; 5 | —; 8 | 1; (15) | —; 5 | Loc | — | Yes |
| Chan et al. [41] | 2018 | *Pa* | Aortic graft | 1 | Yes | 1 (M[z]) | No | 1 | $1 \times 10^7$ | ($1 \times 10^8$) | 10 | — | 1 | — | Loc | 12.5 | Yes |
| Duplessis et al. [42] | 2018 | *Pa* | Bacteremia | 1 | Yes | 0 | Yes | 2 | — | $3.5 \times 10^5$ | — | 6; —[aa] | 6; — | 1.5; — | IV | — | Yes[bb] |
| Ferry et al. [43] | 2018 | *Pa* | Joint | 1 | Yes | 0 | Yes | 4 | $1.2 \times 10^8$ to $9.7 \times 10^8$ per phage | ($\sim 5 \times 10^9$); ($\sim 1 \times 10^{10}$) | 10; 20 | 72 | 4 | (9) | Loc | < 1 to 5 | Yes |
| Fish et al. [44] | 2018 | *Sa* | Diabetic toe ulcers | 1 | Yes | 0 | No | 1[cc] | $1 \times 10^7$ to $1 \times 10^8$ | ($1 \times 10^6$) to ($5 \times 10^7$) | 0.1 to 0.5 | 168 | >>1 | >>1 | Loc | — | Yes |
| Ferry et al. [45] | 2018 | *Pa, Sa* | Prosthetic joint | 1 | Yes | 0 | Yes | 3; 3[dd] | ($3 \times 10^9$) | ($3 \times 10^{10}$) | 10 | — | 1 | — | Loc | — | Yes |
| Fish et al. [46] | 2018 | *Sa* | Diabetic toe ulcers | 2 | Yes | [ee] | No | 1 | $1 \times 10^7$ to $1 \times 10^8$ | ($1 \times 10^6$) to ($5 \times 10^7$) | 0.1 to 0.5 | 168 | >>1 | >>1 | Loc | — | No |
| Hoyle et al. [48] | 2018 | *Ax* | Lung (with cystic fibrosis) | 1 | Yes | 1 (C) | Yes | 2 | ($1.2 \times 10^8$) | ($6 \times 10^8$) | 5 | 24; 12 | (20) | 20[ff] | Neb, PO | — | No |
| LaVergne et al. [49] | 2018 | *Ab* | Surgical wound | 1 | Yes | 0 | No | 1 | $2.1 \times 10^7$ | ($8.5 \times 10^7$) | 4 | 2 | 98 | 8 | IV | (321) | Yes then no |
| McCallin et al. [50] | | *Sa* | Oral safety trial | 21 | No | NA | Yes | 1, Pyo | $1 \times 10^6$, $1 \times 10^5$ | ($1 \times 10^7$), ($1 \times 10^6$) | 10 | 8 | 6 | 2 | IN or POn | — | No |
| Morozova et al. [51] | 2018 | *Sa* | Diabetic toe ulcers | 2 | Yes | 1 (M) | Yes | Pyo | ($5 \times 10^6$) to ($1 \times 10^9$)[gg] | — | — | ≥ 6 | — | ≥ ~336 to ≤ ~504 | Loc | — | (No) |

---

[v] Mostly associated with this species.

[w] This study should be included with that of Międzybrodzki et al. [39], with a ranking of 2.

[x] Shown in this row is the information provided for the two case studies discussed in detail by this publication.

[y] From p. 4 of this publication, "The most active phage was purified and supplied to our hospital…"

[z] The study notes that cultures taken 4 weeks following treatment were negative and also that ongoing infection was deemed lacking based on blood cultures.

[aa] Phage therapy was restarted on day 12, but it is difficult to tell for how long it then continued.

[bb] Assuming that antibiotic treatments were not discontinued upon the start of phage therapy.

[cc] All of the Fish et al. studies are entered in the table as identical in their treatment characteristics, other than whether antibiotic co-treatments were used.

[dd] Three different phages for each targeted bacterium.

[ee] This study should be included with that of Fish et al. [47], with a ranking of 2.

[ff] With this treatment regimen repeated 4 times over the course of one year.

[gg] Phage titers between $10^7$ and $10^{10}$ PFU/ml are diluted two- to 10-fold.

| Authors | Year | Targeted Etiologies | Infection Type(s) | Number Treated | Efficacy Observed? | Phage Impact Score | Cocktail? | # Phage Types | Titer (PFUs/ml) | Dosage (PFUs) | Volume (ml) | Dosing Frequency (hours) | Number of Doses | Dosing Duration (days) | Route | Exotoxin Units (EUs)/ml | Antibiotic Co-treatments? |
|---|---|---|---|---|---|---|---|---|---|---|---|---|---|---|---|---|---|
| Patey et al. [52] | 2018 | *Sa* | Chronic otitis | 15 | Yes | 0 | Yes | Pyo, etc. | — | — | — | — | — | 15 to 30, etc. | IR, Loc, PO | — | Yes |
| Ujmajuridze et al. [53] | 2018 | *En~, Ec, Pa, So~* | Urinary tract | 9 | Yes | 0 | Yes | Pyo | — | — | 20 | 12 | (84) | 7 | ISb | — | No (for 8 of 9) |
| Jennes et al. [54] | 2017 | *Pa* | Septicemia | 1 | Yes | 2 (M) | Yes | 2 | — | — | —; 50 | 24; 8 | (10); (80) | 10 | IV; Loc | — | No |
| Łusiak-Szelachowska et al. [55] | 2017 | *Efs*, Gn, *Sa* | Various | 62 | Yes | [hh] | Yes | ≥ 1 | $1 \times 10^6$ to $1 \times 10^8$ or $1 \times 10^9$ | [ii] | 10; —; 10 | 12; 8 or 12; 8 or 12 | — | ≤ 168 | IR; Loc; POn | — | No |
| Sarker et al. [56] | 2017 | *Ec* | Diarrhea | 38 | No | NA | Yes | 9; etc. | $1 \times 10^5$; $1 \times 10^7$ | $1 \times 10^6$; $1 \times 10^8$ | (10) | (8) | 6 | 2 | PO | — | No (for 14 of 15) |
| Schooley et al. [57] | 2017 | *Ab* | Pancreatitis and disseminated | 1 | Yes | 0 | Yes | 9 | — | $5 \times 10^9$; — | — | 2, 6, 8, or 12 | — | 126 | IV; Loc | [jj]; — | Yes |
| Zhvania et al. [58] | 2017 | *Sa* | Skin | 1 | Yes | 1 (M) | Yes | 1 + Pyo, etc. | $(1 \times 10^7)$[kk] | $(1 \times 10^8)$ | 10 | 24 | 20 × 2 | 20 × 2 | Loc, POn | — | No |
| Fish et al. [47][ll] | 2016 | *Sa* | Diabetic toe ulcers | 9 | Yes | 2 (C) | No | 1 | $1 \times 10^7$ to $1 \times 10^8$ | $(1 \times 10^6)$ to $(5 \times 10^7)$ | 0.1 to 0.5 | 168 | >>1 | >>1 | Loc | — | No |
| Sarker et al. [59] | 2016 | *Ec* | Diarrhea | 15 | No | NA | Yes | 11; etc. | $(1.2 \times 10^7)$; $(4.7 \times 10^7)$ | $3.6 \times 10^8$; $1.4 \times 10^9$ | 30 | 8 | (12) | 4 | POn | — | No |
| Fadlallah et al. [60] | 2015 | *Sa* | Ocular | 1 | Yes | 1 (M) | No | 1 | — | — | — | — | — | 28 | IV, Loc | — | No[mm] |
| Kutateladze [61] | 2015 | Various | Various | 1,000s | Yes | 0 | Yes | Pyo, etc. | — | — | — | — | — | — | — | — | — |
| Łusiak-Szelachowska et al. [62] | 2014 | *Efs, Kp, Pa, Sme, Enc, Sa* | Various | 15 | No | NA | Yes | — | — | — | 10 to 20; —; 10 to 20 | 12; 12; 8 | — | ≤ 168 | IR; Loc; POn | < 5 | — |
| Rose et al. [63] | 2014 | *Pa, Sa* | Burn wound | 9 | No | NA | Yes | 3 | $3 \times 10^9$ | $1 \times 10^7$ | 0.03 | — | 1 | — | Loc | [nn] | Yes |
| Lecion et al. [64] | 2013 | *Sa* | Various | 6 | Yes | 1 (M) | No | 3[oo] | $3.6 \times 10^7$ to $6.3 \times 10^8$ | — | 2 to 3; 10 | ~5 to 24; 8 | — | ≥ 28 | Loc; PO | — | No |
| McCallin et al. [65] | 2014 | *Ec, Pr~* | Oral safety trial | 10 | No | NA | Yes | 18 | $> 7 \times 10^6$ | — | 10, 20 | — | 3 | 2 | POn | — | — |

[hh] This study should be included with that of Międzybrodzki et al. [39], with a ranking of 2.

[ii] Ten-fold higher than indicated titers for IR and PO. Uncertain for Loc.

[jj] Phages were diluted to result in no more than 5 endotoxin units per kg body mass per hour.

[kk] This value is reported without units so we are assuming that the intended units were PFUs/ml. Note that three different phage preparations were used with information reported in this row on a per individual-preparation basis.

[ll] This case is discussed in conjunction with and under the heading of Fish et al.. [46]

[mm] A lack of antibiotic treatment in association with phage treatment is, however, not explicitly stated.

[nn] From p. 68, each phage was “purified of endotoxin”.

[oo] Though three different phages are named, it is not obvious that they were used in combination.

| Authors | Year | Targeted Etiologies | Infection Type(s) | Number Treated | Efficacy Observed? | Phage Impact Score | Cocktail? | # Phage Types | Titer (PFUs/ml) | Dosage (PFUs) | Volume (ml) | Dosing Frequency (hours) | Number of Doses | Dosing Duration (days) | Route | Exotoxin Units (EUs)/ml | Antibiotic Co-treatments? |
|---|---|---|---|---|---|---|---|---|---|---|---|---|---|---|---|---|---|
| Międzybrodzki et al. [39] | 2012 | *Ci~, En~, Eb~, Ec, Pr~, Sl~, Sn~* | Various | 153 | No[pp] | 2 (M[qq]) | No | — | $1 \times 10^6$ to $1 \times 10^9$ | — | 10 to 20; —; —; 10 to 20 | 12; —; —; 8 | — | ≤ 168 | IR; Neb; Loc; POn | — | Yes or no |
| Sarker et al. [66] | 2012 | *Ec* | Oral safety trial | 9 | No | NA | Yes | 9 | $6 \times 10^5$; $6 \times 10^7$ | $3 \times 10^7$; $3 \times 10^9$ | 50 | — | 3 | 1 | POn | — | No |
| Khawaldeh et al. [67] | 2011 | *Pa* | Urinary tract | 1 | Yes | 0 | Yes | 6 | $(1 \times 10^{6)}$ | $2 \times 10^7$ | 20 | 12 | (20) | 10 | ISb | — | Yes |
| Kvachadze et al. [68] | 2011 | *Pa, Sa* | Lung (with cystic fibrosis) | 1 | Yes | 1 (M) | Yes | 1 + Pyo | — | — | — | — | 9[rr] | — | Neb | — | Yes |
| Letkiewicz et al. [69] | 2010 | *Efs, Ec, Kp, Pa, Sa* | Chronic prostatitis | 22 | Yes | [ss] | — | — | — | — | — | — | — | 22-99 | IR, PO, Loc | — | — |
| Letkiewicz et al. [70] | 2009 | *Efs* | Chronic prostatitis | 2 | Yes | 2 (M) | No | [tt] | $\sim 1 \times 10^8$ | $(\sim 1 \times 10^9)$ | 10 | 12 | (≥ 56) | 28 to 33 | IR | — | No |
| Rhoads et al. [71] | 2009 | *Ec, Pa, Sa* | Venous leg ulcers | 39 | No | NA | Yes | 8 | $(8 \times 10^9)$ | — | — | 168 | (12) | 84 | Loc | — | Yes[uu] |
| Wright et al. [72] | 2009 | *Pa* | Chronic otitis | 12 | Yes | 1 (M) | Yes | 6 | $3 \times 10^7$ | $6 \times 10^5$ | 0.2 | — | 1 | — | Loc | — | No |
| Kutateladze and Adamia [73] | 2008 | *Sa* | Sepsis | 46[vv] | Yes | 2 (C[ww]) | — | — | — | — | — | — | — | — | — | — | Yes |
| Leszczynski et al. [74] | 2006 | *Sa* | Gut decolonization | 1 | Yes | 2 (M) | Yes | 3 | $7 \times 10^8$ | $(7 \times 10^9)$ | (10) | 8 | (84) | 28 | POn | — | No |
| Marza et al. [75] | 2006 | *Pa* | Burn wound | 1 | Yes | 0 | — | — | $(5 \times 10^3)$ | $1 \times 10^3$ | 0.2 | — | 1 | — | Loc | — | Yes |
| Weber-Dąbrowska et al. [76] | 2006 | *Se, Sho* | Otitis media | 1 | Yes | 0 | Yes | 2 | — | — | — | — | — | —, 21 | PO, Loc | — | No |

---

[pp] From p. 87, "In the case of a mixed infection with two bacterial strains at the same time, if it was possible to prepare phages against both bacteria, the patient was administered individual doses of both phage preparations alternately."

[qq] Clinical successes, however, tend to outnumber microbiologically determined successes. In addition, microbiological success is lumped in with clinical success, i.e., with "pathogen eradication and/or recovery (pathogen eradication was confirmed by the results of bacterial cultures; recovery refers to wound healing or complete subsidence of the infection symptoms)" (p. 87), as representing their top-ranking category of treatment success.

[rr] Doses were provided every 4 to 6 weeks.

[ss] This study should be included with that of Letkiewicz et al. [70], with a ranking of 2.

[tt] It seems that different phages were used on different patients and we infer that these were single phages being used from the statement, "The phage titer in the preparation…"

[uu] From p. 239 [71]: "Antibiotic administration to patients in the test and control groups was permitted if clinical signs of acute wound infection (redness, heat, tenderness, oedema) were observed."

[vv] As treated with phages, only. The publication also considers the treatment of additional pathogens in additional contexts.

[ww] As this was treatment of sepsis, it is possible that total recovery was characterized microbiologically, though this is not stated explicitly in the publication.

| Authors | Year | Targeted Etiologies | Infection Type(s) | Number Treated | Efficacy Observed? | Phage Impact Score | Cocktail? | # Phage Types | Titer (PFUs/ml) | Dosage (PFUs) | Volume (ml) | Dosing Frequency (hours) | Number of Doses | Dosing Duration (days) | Route | Exotoxin Units (EUs)/ml | Antibiotic Co-treatments? |
|---|---|---|---|---|---|---|---|---|---|---|---|---|---|---|---|---|---|
| Bruttin and Brüssow [77] | 2005 | *Ec* | Oral safety trial | 15 | No | NA | No | 1 | $1 \times 10^3$; $1 \times 10^5$ | ($1.5 \times 10^5$); ($1.5 \times 10^7$) | 150 | — | 1; 1 | — | POn | —[xx] | No |
| Jikia et al. [78] | 2005 | *Sa* | Radiation burn | 2 | Yes | 1 (M) | Yes | Pyo | — | — | — | — | 1 | — | Loc | — | Yes |
| Weber-Dąbrowska et al. [79] | 2003 | *Ec, Kp, Pa, Sa* | Sepsis | 94 | Yes[yy] | 2 (C) | — | — | — | — | 5, 10 | 8 | — | 29[zz] | POn | — | Yes |
| Markoishvili et al. [80] | 2002 | *Ec ,Pa, Pr~, Sa, Se, So~* | Wound | 107 | Yes | 0 | Yes | Pyo | — | [aaa] | — | ~72 to ~168 | — | 6 to ~450 | Loc | — | Yes |
| Weber-Dąbrowska et al. [81] | 2002 | *Ec, Ko, Mm, Sa, Se, Sho, Ssa, Ssi* | Various | 42 | No | NA | — | — | — | — | — | 8 | (63) | 21 | POn | — | — |
| Weber-Dąbrowska et al., [82] | 2001 | *Ec, Ko Kp, Pa, Sa* | Various | 20 | Yes | 0 (C) | — | — | $1 \times 10^8$ | — | — | 8 | — | 14 to 63 | Loc, PO | — | — |
| Weber-Dąbrowska et al. [83] | 2000 | *Eb~, Ec, Kl~, Pr~, Ps~, Sa* | Suppurative | 1307 | Yes | 1 (C) | — | — | — | — | — | —; 8 | — | 7 to 84 | IP or Loc; POn | — | — |
| Weber-Dąbrowska et al. [84] | 2000 | *Es~, Kl~, Ps~, Sy~* | Suppurative | 51 | Yes | 0 | — | — | — | — | — | — | — | — | Loc, PO | — | — |

[xx] Endotoxin levels were measured, but not in the dosed phage volume nor convertible to that volume.

[yy] That cocktails were used we infer from the treatment of mixed infections.

[zz] Median treatment duration.

[aaa] Doses are indicated as $1 \times 10^6$ PFU/cm$^2$ of PhagoBioDerm.

## References


1. Cano, E. J.; Caflisch, K. M.; Bollyky, P. L.; Van Belleghem, J. D.; Patel, R.; Fackler, J.; Brownstein, M. J.; Horne, B.; Biswas, B.; Henry, M.; et al. Phage therapy for limb-threatening prosthetic knee *Klebsiella pneumoniae* infection: case report and *in vitro* characterization of anti-biofilm activity. *Clin. Infect. Dis.* **2021,** *73*, e144-e151.

2. Dedrick, R. M.; Freeman, K. G.; Nguyen, J. A.; Bahadirli-Talbott, A.; Smith, B. E.; Wu, A. E.; Ong, A. S.; Lin, C. T.; Ruppel, L. C.; Parrish, N. M.; et al. Potent antibody-mediated neutralization limits bacteriophage treatment of a pulmonary *Mycobacterium abscessus* infection. *Nat. Med.* **2021,** *27*, 1357-1361.

3. Doub, J. B.; Ng, V. Y.; Wilson, E.; Corsini, L.; Chan, B. K. Successful treatment of a recalcitrant *Staphylococcus epidermidis* prosthetic knee infection with intraoperative bacteriophage therapy. *Pharmaceuticals* **2021,** *14*.

4. Ferry, T.; Kolenda, C.; Batailler, C.; Gaillard, R.; Gustave, C. A.; Lustig, S.; Fevre, C.; Petitjean, C.; Leboucher, G.; Laurent, F. Case report: Arthroscopic "Debridement Antibiotics and Implant Retention" with local injection of personalized phage therapy to salvage a relapsing *Pseudomonas aeruginosa* prosthetic knee infection. *Front Med. (Lausanne)* **2021,** *8*, 569159.

5. Johri, A. V.; Johri, P.; Hoyle, N.; Pipia, L.; Nadareishvili, L.; Nizharadze, D. Case report: Chronic bacterial prostatitis treated with phage therapy after multiple failed antibiotic treatments. *Front Pharmacol.* **2021,** *12*, 692614.

6. Lebeaux, D.; Merabishvili, M.; Caudron, E.; Lannoy, D.; Van Simaey, L.; Duyvejonck, H.; Guillemain, R.; Thumerelle, C.; Podglajen, I.; Compain, F.; et al. A case of phage therapy against pandrug-resistant *Achromobacter xylosoxidans* in a 12-year-old lung-transplanted cystic fibrosis patient. *Viruses* **2021,** *13*.

7. Leitner, L.; Ujmajuridze, A.; Chanishvili, N.; Goderdzishvili, M.; Chkonia, I.; Rigvava, S.; Chkhotua, A.; Changashvili, G.; McCallin, S.; Schneider, M. P.; et al. Intravesical bacteriophages for treating urinary tract infections in patients undergoing transurethral resection of the prostate: a randomised, placebo-controlled, double-blind clinical trial. *Lancet Infect. Dis.* **2021,** *21*, 427-436.

8. Lusiak-Szelachowska, M.; Miedzybrodzki, R.; Fortuna, W.; Borysowski, J.; Górski, A. Anti-phage serum antibody responses and the outcome of phage therapy. *Folia Microbiol.* **2021,** *66*, 127-131.

9. Ramirez-Sanchez, C.; Gonzales, F.; Buckley, M.; Biswas, B.; Henry, M.; Deschenes, M. V.; Horne, B.; Fackler, J.; Brownstein, M. J.; Schooley, R. T.; et al. Successful treatment of *Staphylococcus aureus* prosthetic joint infection with bacteriophage therapy. *Viruses* **2021,** *13*.

10. Rostkowska, O. M.; Miedzybrodzki, R.; Miszewska-Szyszkowska, D.; Górski, A.; Durlik, M. Treatment of recurrent urinary tract infections in a 60-year-old kidney transplant recipient. The use of phage therapy. *Transpl. Infect. Dis.* **2021,** *23*, e13391.

11. Tan, X.; Chen, H.; Zhang, M.; Zhao, Y.; Jiang, Y.; Liu, X.; Huang, W.; Ma, Y. Clinical experience of personalized phage therapy against carbapenem-resistant *Acinetobacter baumannii* lung infection in a patient with chronic obstructive pulmonary disease. *Front Cell Infect. Microbiol.* **2021,** *11*, 631585.

12. Wu, N.; Dai, J.; Guo, M.; Li, J.; Zhou, X.; Li, F.; Gao, Y.; Qu, H.; Lu, H.; Jin, J.; et al. Pre-optimized phage therapy on secondary *Acinetobacter baumannii* infection in four critical COVID-19 patients. *Emerg. Microbes. Infect.* **2021,** *10*, 612-618.

13. Aslam, S.; Lampley, E.; Wooten, D.; Karris, M.; Benson, C.; Strathdee, S.; Schooley, R. T. Lessons learned from the first 10 consecutive cases of intravenous bacteriophage therapy to treat multidrug-resistant bacterial infections at a single center in the United States. *Open Forum Infect. Dis.* **2020,** *7*, ofaa389.

14. Bao, J.; Wu, N.; Zeng, Y.; Chen, L.; Li, L.; Yang, L.; Zhang, Y.; Guo, M.; Li, L.; Li, J.; et al. Non-active antibiotic and bacteriophage synergism to successfully treat recurrent urinary tract infection caused by extensively drug-resistant *Klebsiella pneumoniae*. *Emerg. Microbes. Infect.* **2020,** *9*, 771-774.

15. Corbellino, M.; Kieffer, N.; Kutateladze, M.; Balarjishvili, N.; Leshkasheli, L.; Askilashvili, L.; Tsertsvadze, G.; Rimoldi, S. G.; Nizharadze, D.; Hoyle, N.; et al. Eradication of a multidrug-resistant, carbapenemase-producing *Klebsiella pneumoniae* isolate following oral and intra-rectal therapy with a custom made, lytic bacteriophage preparation. *Clin. Infect. Dis.* **2020,** *70*, 1998-2001.

16. Doub, J. B.; Ng, V. Y.; Johnson, A. J.; Slomka, M.; Fackler, J.; Horne, B.; Brownstein, M. J.; Henry, M.; Malagon, F.; Biswas, B. Salvage bacteriophage therapy for a chronic MRSA prosthetic joint infection. *Antibiotics (Basel)* **2020,** *9*.

17. Ferry, T.; Batailler, C.; Petitjean, C.; Chateau, J.; Fevre, C.; Forestier, E.; Brosset, S.; Leboucher, G.; Kolenda, C.; Laurent, F.; et al. The potential innovative use of bacteriophages within the DAC((R)) hydrogel to treat patients with knee megaprosthesis infection requiring "Debridement antibiotics and implant retention" and soft tissue coverage as salvage therapy. *Front Med. (Lausanne)* **2020,** *7*, 342.

18. Ferry, T.; Kolenda, C.; Batailler, C.; Gustave, C. A.; Lustig, S.; Malatray, M.; Fevre, C.; Josse, J.; Petitjean, C.; Chidiac, C.; et al. Phage therapy as adjuvant to conservative surgery and antibiotics to salvage patients with relapsing *S. aureus* prosthetic knee infection. *Front Med. (Lausanne)* **2020,** *7*, 570572.

19. Gainey, A. B.; Burch, A. K.; Brownstein, M. J.; Brown, D. E.; Fackler, J.; Horne, B.; Biswas, B.; Bivens, B. N.; Malagon, F.; Daniels, R. Combining bacteriophages with cefiderocol and meropenem/vaborbactam to treat a pan-drug resistant *Achromobacter* species infection in a pediatric cystic fibrosis patient. *Pediatr. Pulmonol.* **2020,** *55*, 2990-2994.

20. Grubb, D. S.; Wrigley, S. D.; Freedman, K. E.; Wei, Y.; Vazquez, A. R.; Trotter, R. E.; Wallace, T. C.; Johnson, S. A.; Weir, T. L. PHAGE-2 study: supplemental bacteriophages extend *Bifidobacterium animalis* subsp. *lactis* BL04 benefits on gut health and microbiota in healthy adults. *Nutrients.* **2020,** *12*.

21. Petrovic Fabijan, P.; .; Lin, R. C. Y.; Ho, J.; Maddocks, S.; Ben Zakour, N. L.; Iredell, J. R.; Westmead Bacteriophage Therapy Team Safety of bacteriophage therapy in severe *Staphylococcus aureus* infection. *Nat. Microbiol.* **2020,** *5*, 465-472.

22. Qin, J.; Wu, N.; Bao, J.; Shi, X.; Ou, H.; Ye, S.; Zhao, W.; Wei, Z.; Cai, J.; Li, L.; et al. Heterogeneous *Klebsiella pneumoniae* co-infections complicate personalized bacteriophage therapy. *Front Cell Infect. Microbiol.* **2020,** *10*, 608402.

23. Rubalskii, E.; Ruemke, S.; Salmoukas, C.; Boyle, E. C.; Warnecke, G.; Tudorache, I.; Shrestha, M.; Schmitto, J. D.; Martens, A.; Rojas, S. V.; et al. Bacteriophage therapy for critical infections related to cardiothoracic surgery. *Antibiotics (Basel)* **2020,** *9*.

24. Aslam, S.; Courtwright, A. M.; Koval, C.; Lehman, S. M.; Morales, S.; Furr, C.-L. L.; Rosas, F.; Brownstein, M. J.; Fackler, J. R.; Sisson, B. M.; et al. Early clinical experience of bacteriophage therapy in three lung transplant recipients. *Am. J Transplant.* **2019,** *19*, 2631-2639.

25. Aslam, S.; Pretorius, V.; Lehman, S. M.; Morales, S.; Schooley, R. T. Novel bacteriophage therapy for treatment of left ventricular assist device infection. *J Heart Lung Transplant.* **2019,** *38*, 475-476.

26. Dedrick, R. M.; Guerrero-Bustamante, C. A.; Garlena, R. A.; Russell, D. A.; Ford, K.; Harris, K.; Gilmour, K. C.; Soothill, J.; Jacobs-Sera, D.; Schooley, R. T.; et al. Engineered bacteriophages for treatment of a patient with a disseminated drug-resistant *Mycobacterium abscessus*. *Nat. Med.* **2019,** *25*, 730-733.

27. Febvre, H. P.; Rao, S.; Gindin, M.; Goodwin, N. D. M.; Finer, E.; Vivanco, J. S.; Lu, S.; Manter, D. K.; Wallace, T. C.; Weir, T. L. PHAGE study: effects of supplemental bacteriophage intake on inflammation and gut microbiota in healthy adults. *Nutrients.* **2019,** *11*.

28. Gilbey, T.; Ho, J.; Cooley, L. A.; Petrovic, F. A.; Iredell, J. R. Adjunctive bacteriophage therapy for prosthetic valve endocarditis due to *Staphylococcus aureus*. *Med. J Aust.* **2019,** *211*, 142-143.

29. Gindin, M.; Febvre, H. P.; Rao, S.; Wallace, T. C.; Weir, T. L. Bacteriophage for gastrointestinal health (PHAGE) study: evaluating the safety and tolerability of supplemental bacteriophage consumption. *J. Am. Coll. Nutr.* **2019,** *38*, 68-75.

30. Gupta, P.; Singh, H. S.; Shukla, V. K.; Nath, G.; Bhartiya, S. K. Bacteriophage therapy of chronic nonhealing wound: clinical study. *Int. J Low Extrem. Wounds.* **2019,** *18*, 171-175.

31. Jault, P.; Leclerc, T.; Jennes, S.; Pirnay, J. P.; Que, Y. A.; Resch, G.; Rousseau, A. F.; Ravat, F.; Carsin, H.; Le, F. R.; et al. Efficacy and tolerability of a cocktail of bacteriophages to treat burn wounds infected by *Pseudomonas aeruginosa* (PhagoBurn): a randomised, controlled, double-blind phase 1/2 trial. *Lancet Infect. Dis.* **2019,** *19*, 35-45.

32. Kuipers, S.; Ruth, M. M.; Mientjes, M.; de Sevaux, R. G. L.; van, I. J. A Dutch case report of successful treatment of chronic relapsing urinary tract infection with bacteriophages in a renal transplant patient. *Antimicrob. Agents Chemother.* **2019,** *64*, e01281-19.

33. Law, N.; Logan, C.; Yung, G.; Furr, C. L. L.; Lehman, S. M.; Morales, S.; Rosas, F.; Gaidamaka, A.; Bilinsky, I.; Grint, P. Successful adjunctive use of bacteriophage therapy for treatment of multidrug-resistant *Pseudomonas aeruginosa* infection in a cystic fibrosis patient. *Infection* **2019,** *47*, 665-668.

34. Maddocks, S.; Petrovic Fabijan, P.; .; Ho, J.; Lin, R. C. Y.; Ben Zakour, N. L.; Dugan, C.; Kliman, I.; Branston, S.; Morales, S.; et al. Bacteriophage therapy of ventilator-associated pneumonia and empyema caused by *Pseudomonas aeruginosa*. *Am. J. Respir. Crit Care Med.* **2019,** *200*, 1179-1181.

35. Nir-Paz, R.; Gelman, D.; Khouri, A.; Sisson, B. M.; Fackler, J.; Alkalay-Oren, S.; Khalifa, L.; Rimon, A.; Yerushalmy, O.; Bader, R.; et al. Successful treatment of antibiotic resistant poly-microbial bone infection with bacteriophages and antibiotics combination. *Clin. Infect. Dis.* **2019,** *69*, 2015-2018.

36. Onsea, J.; Soentjens, P.; Djebara, S.; Merabishvili, M.; Depypere, M.; Spriet, I.; De, M. P.; Debaveye, Y.; Nijs, S.; Vanderschot, P.; et al. Bacteriophage application for difficult-to-treat musculoskeletal infections: development of a standardized multidisciplinary treatment protocol. *Viruses* **2019,** *11*.

37. Ooi, M. L.; Drilling, A. J.; Morales, S.; Fong, S.; Moraitis, S.; Macias-Valle, L.; Vreugde, S.; Psaltis, A. J.; Wormald, P. J. Safety and tolerability of bacteriophage therapy for chronic rhinosinusitis due to *Staphylococcus aureus*. *JAMA Otolaryngol. Head Neck Surg* **2019,** *145*, 723-729.

38. Rogóz, P.; Amanatullah, D. F.; Miedzybrodzki, R.; Manasherob, R.; Weber-Dabrowska, B.; Fortuna, W.; Letkiewicz, S.; Górski, A. Phage therapy in orthopaedic implant-associated infections. In *Phage Therapy: A Practical Approach*, Górski, A., Miedzybrodzki, R.; Borysowski, J., Eds.; Springer: **2019**; 189-211.

39. Miedzybrodzki, R.; Borysowski, J.; Weber-Dabrowska, B.; Fortuna, W.; Letkiewicz, S.; Szufnarowski, K.; Pawelczyk, Z.; Rogoz, P.; Klak, M.; Wojtasik, E.; et al. Clinical aspects of phage therapy. *Adv. Virus Res.* **2012,** *83*, 73-121.

40. Tkhilaishvili, T.; Winkler, T.; Muller, M.; Perka, C.; Trampuz, A. Bacteriophages as adjuvant to antibiotics for the treatment of periprosthetic joint infection caused by multidrug-resistant *Pseudomonas aeruginosa*. *Antimicrob. Agents Chemother.* **2019,** *64*, e00924-19.

41. Chan, B. K.; Turner, P. E.; Kim, S.; Mojibian, H. R.; Elefteriades, J. A.; Narayan, D. Phage treatment of an aortic graft infected with *Pseudomonas aeruginosa*. *Evol. Med. Pub. Health* **2018,** *1*, 60-66.

42. Duplessis, C.; Biswas, B.; Hanisch, B.; Perkins, M.; Henry, M.; Quinones, J.; Wolfe, D.; Estrella, L.; Hamilton, T. Refractory *Pseudomonas* bacteremia in a 2-year-old sterilized by bacteriophage therapy. *J. Pediatric Infect. Dis. Soc.* **2018,** *7*, 253-256.

43. Ferry, T.; Boucher, F.; Fevre, C.; Perpoint, T.; Chateau, J.; Petitjean, C.; Josse, J.; Chidiac, C.; L'Hostis, G.; Leboucher, G.; et al. Innovations for the treatment of a complex bone and joint infection due to XDR *Pseudomonas aeruginosa* including local application of a selected cocktail of bacteriophages. *J Antimicrob. Chemother.* **2018,** *73*, 2901-2903.

44. Fish, R.; Kutter, E.; Bryan, D.; Wheat, G.; Kuhl, S. Resolving digital staphylococcal osteomyelitis using bacteriophage-a case report. *Antibiotics (Basel)* **2018,** *7*.

45. Ferry, T.; Leboucher, G.; Fevre, C.; Herry, Y.; Conrad, A.; Josse, J.; Batailler, C.; Chidiac, C.; Medina, M.; Lustig, S.; et al. Salvage debridement, antibiotics and implant retention ("DAIR") with local injection of a selected cocktail of bacteriophages: is it an option for an elderly patient with relapsing *Staphylococcus aureus* prosthetic-joint infection? *Open Forum Infect. Dis.* **2018,** *5*, ofy269.

46. Fish, R.; Kutter, E.; Wheat, G.; Blasdel, B.; Kutateladze, M.; Kuhl, S. Compassionate use of bacteriophage therapy for foot ulcer treatment as an effective step for moving toward clinical trials. *Meth. Mol. Biol.* **2018,** *1693*, 159-170.

47. Fish, R.; Kutter, E.; Wheat, G.; Blasdel, B.; Kutateladze, M.; Kuhl, S. Bacteriophage treatment of intransigent diabetic toe ulcers: a case series. *J. Wound Care* **2016,** *25 Suppl 7*, S27-S33.

48. Hoyle, N.; Zhvaniya, P.; Balarjishvili, N.; Bolkvadze, D.; Nadareishvili, L.; Nizharadze, D.; Wittmann, J.; Rohde, C.; Kutateladze, M. Phage therapy against *Achromobacter xylosoxidans* lung infection in a patient with cystic fibrosis: a case report. *Res. Microbiol.* **2018,** *169*, 540-542.

49. LaVergne, S.; Hamilton, T.; Biswas, B.; Kumaraswamy, M.; Schooley, R. T.; Wooten, D. Phage therapy for a multidrug-resistant *Acinetobacter baumannii* craniectomy site infection. *Open Forum Infect. Dis.* **2018,** *5*, ofy064.

50. McCallin, S.; Sarker, S. A.; Sultana, S.; Oechslin, F.; Brussow, H. Metagenome analysis of Russian and Georgian Pyophage cocktails and a placebo-controlled safety trial of single phage versus phage cocktail in healthy *Staphylococcus aureus* carriers. *Environ. Microbiol.* **2018,** *20*, 3278-3293.

51. Morozova, V. V.; Kozlova, Y. N.; Ganichev, D. A.; Tikunova, N. V. Bacteriophage treatment of infected diabetic foot ulcers. *Meth. Mol. Biol.* **2018,** *1693*, 151-158.

52. Patey, O.; McCallin, S.; Mazure, H.; Liddle, M.; Smithyman, A.; Dublanchet, A. Clinical indications and compassionate use of phage therapy: personal experience and literature review with a focus on osteoarticular infections. *Viruses.* **2018,** *11*.

53. Ujmajuridze, A.; Chanishvili, N.; Goderdzishvili, M.; Leitner, L.; Mehnert, U.; Chkhotua, A.; Kessler, T. M.; Sybesma, W. Adapted bacteriophages for treating urinary tract infections. *Front. Microbiol.* **2018,** *9*, 1832.

54. Jennes, S.; Merabishvili, M.; Soentjens, P.; Pang, K. W.; Rose, T.; Keersebilck, E.; Soete, O.; Francois, P. M.; Teodorescu, S.; Verween, G.; et al. Use of bacteriophages in the treatment of colistin-only-sensitive *Pseudomonas aeruginosa* septicaemia in a patient with acute kidney injury-a case report. *Crit. Care* **2017,** *21*, 129.

55. Lusiak-Szelachowska, M.; Zaczek, M.; Weber-Dabrowska, B.; Miedzybrodzki, R.; Letkiewicz, S.; Fortuna, W.; Rogoz, P.; Szufnarowski, K.; Jonczyk-Matysiak, E.; Olchawa, E.; et al. Antiphage activity of sera during phage therapy in relation to its outcome. *Future Microbiol.* **2017,** *12*, 109-117.

56. Sarker, S. A.; Berger, B.; Deng, Y.; Kieser, S.; Foata, F.; Moine, D.; Descombes, P.; Sultana, S.; Huq, S.; Bardhan, P. K.; et al. Oral application of *Escherichia coli* bacteriophage: safety tests in healthy and diarrheal children from Bangladesh. *Environ. Microbiol.* **2017,** *19*, 237-250.

57. Schooley, R. T.; Biswas, B.; Gill, J. J.; Hernandez-Morales, A.; Lancaster, J.; Lessor, L.; Barr, J. J.; Reed, S. L.; Rohwer, F.; Benler, S.; et al. Development and use of personalized bacteriophage-based therapeutic cocktails to treat a patient with a disseminated resistant *Acinetobacter baumannii* infection. *Antimicrob. Agents Chemother.* **2017,** *61*, e00954-17.

58. Zhvania, P.; Hoyle, N. S.; Nadareishvili, L.; Nizharadze, D.; Kutateladze, M. Phage therapy in a 16-year-old boy with Netherton syndrome. *Front Med. (Lausanne)* **2017,** *4*, 94.

59. Sarker, S. A.; Sultana, S.; Reuteler, G.; Moine, D.; Descombes, P.; Charton, F.; Bourdin, G.; McCallin, S.; Ngom-Bru, C.; Neville, T.; et al. Oral phage therapy of acute bacterial diarrhea with two coliphage preparations: a randomized trial in children from Bangladesh. *EBioMedicine* **2016,** *4*, 124-137.

60. Fadlallah, A.; Chelala, E.; Legeais, J. M. Corneal infection therapy with topical bacteriophage administration. *Open. Ophthalmol. J.* **2015,** *9*, 167-168.

61. Kutateladze, M. Experience of the Eliava Institute in bacteriophage therapy. *Virol. Sin.* **2015,** *30*, 80-81.

62. Lusiak-Szelachowska, M.; Zaczek, M.; Weber-Dabrowska, B.; Miedzybrodzki, R.; Klak, M.; Fortuna, W.; Letkiewicz, S.; Rogoz, P.; Szufnarowski, K.; Jonczyk-Matysiak, E.; et al. Phage neutralization by sera of patients receiving phage therapy. *Viral Immunol.* **2014,** *27*, 295-304.

63. Rose, T.; Verbeken, G.; Vos, D. D.; Merabishvili, M.; Vaneechoutte, M.; Lavigne, R.; Jennes, S.; Zizi, M.; Pirnay, J. P. Experimental phage therapy of burn wound infection: difficult first steps. *Int. J. Burns Trauma* **2014,** *4*, 66-73.

64. Lecion, D.; Fortuna, W.; Dabrowska, K.; Miedzybrodzki, R.; Weber-Dabrowska, B.; Górski, A. Application of microbiological quantitative methods for evaluation of changes in the amount of bacteria in patients with wounds and purulent fistulas subjected to phage therapy and for assessment of phage preparation effectiveness (*in vitro* studies). *Adv. Med. Sci.* **2013,** *2*, 1-8.

65. McCallin, S.; Alam, S. S.; Barretto, C.; Sultana, S.; Berger, B.; Huq, S.; Krause, L.; Bibiloni, R.; Schmitt, B.; Reuteler, G.; et al. Safety analysis of a Russian phage cocktail: from metaGenomic analysis to oral application in healthy human subjects. *Virology* **2013,** *443*, 187-196.

66. Sarker, S. A.; McCallin, S.; Barretto, C.; Berger, B.; Pittet, A. C.; Sultana, S.; Krause, L.; Huq, S.; Bibiloni, R.; Bruttin, A.; et al. Oral T4-like phage cocktail application to healthy adult volunteers from Bangladesh. *Virology* **2012,** *434*, 222-232.

67. Khawaldeh, A.; Morales, S.; Dillon, B.; Alavidze, Z.; Ginn, A. N.; Thomas, L.; Chapman, S. J.; Dublanchet, A.; Smithyman, A.; Iredell, J. R. Bacteriophage therapy for refractory *Pseudomonas aeruginosa* urinary tract infection. *J Med. Microbiol.* **2011,** *60*, 1697-1700.

68. Kvachadze, L.; Balarjishvili, N.; Meskhi, T.; Tevdoradze, E.; Skhirtladze, N.; Pataridze, T.; Adamia, R.; Topuria, T.; Kutter, E.; Rohde, C.; et al. Evaluation of lytic activity of staphylococcal bacteriophage Sb-1 against freshly isolated clinical pathogens. *Microb. Biotechnol.* **2011,** *4*, 643-650.

69. Letkiewicz, S.; Miedzybrodzki, R.; Klak, M.; Jonczyk, E.; Weber-Dabrowska, B.; Górski, A. The perspectives of the application of phage therapy in chronic bacterial prostatitis. *FEMS Immunol. Med. Microbiol.* **2010,** *60*, 99-112.

70. Letkiewicz, S.; Miedzybrodzki, R.; Fortuna, W.; Weber-Dabrowska, B.; Górski, A. Eradication of *Enterococcus faecalis* by phage therapy in chronic bacterial prostatitis--case report. *Folia Microbiol.* **2009,** *54*, 457-461.

71. Rhoads, D. D.; Wolcott, R. D.; Kuskowski, M. A.; Wolcott, B. M.; Ward, L. S.; Sulakvelidze, A. Bacteriophage therapy of venous leg ulcers in humans: results of a phase I safety trial. *J. Wound Care* **2009,** *18*, 237-244.

72. Wright, A.; Hawkins, C. H.; Anggård, E. E.; Harper, D. R. A controlled clinical trial of a therapeutic bacteriophage preparation in chronic otitis due to antibiotic-resistant *Pseudomonas aeruginosa*; a preliminary report of efficacy. *Clin. Otolaryng.* **2009,** *34*, 349-357.

73. Kutateladze, M.; Adamia, R. Phage therapy experience at the Eliava Institute. *Med. Mal. Infect.* **2008,** *38*, 426-430.

74. Leszczynski, P.; Weber-Dabrowska, B.; Kohutnicka, M.; Luczak, M.; Górecki, A.; Górski, A. Successful eradication of methicillin-resistant *Staphylococcus aureus* (MRSA) intestinal carrier status in a healthcare worker - a case report. *Folia Microbiol.* **2006,** *51*, 236-238.

75. Marza, J. A. S.; Soothill, J. S.; Boydell, P.; Collyns, T. A. Multiplication of therapeutically administered bacteriophages in *Pseudomonas aeruginosa* infected patients. *Burns* **2006,** *32*, 644-646.

76. Weber-Dabrowska, B.; Zimecki, M.; Kruzel, M.; Kochanowska, I.; Lusiak-Szelachowska, M. Alternative therapies in antibiotic-resistant infection. *Adv. Med. Sci.* **2006,** *51*, 242-244.

77. Bruttin, A.; Brüssow, H. Human volunteers receiving *Escherichia coli* phage T4 orally: a safety test of phage therapy. *Antimicrob. Agents Chemother.* **2005,** *49*, 2874-2878.

78. Jikia, D.; Chkhaidze, N.; Imedashvili, E.; Mgaloblishvili, I.; Tsitlanadze, G.; Katsarava, R.; Glenn Morris, J. J.; Sulakvelidze, A. The use of a novel biodegradable preparation capable of the sustained release of bacteriophages and ciprofloxacin, in the complex treatment of multidrug-resistant *Staphylococcus aureus*-infected local radiation injuries caused by exposure to $Sr^{90}$. *Clin. Exp. Dermatol.* **2005,** *30*, 23-26.

79. Weber-Dabrowska, B.; Mulczyk, M.; Górski, A. Bacteriophages as an efficient therapy for antibiotic-resistant septicemia in man. *Transplant. Proc.* **2003,** *35*, 1385-1386.

80. Markoishvili, K.; Tsitlanadze, G.; Katsarava, R.; Morris, J. G., Jr.; Sulakvelidze, A. A novel sustained-release matrix based on biodegradable poly(ester amide)s and impregnated with bacteriophages and an antibiotic shows promise in management of infected venous stasis ulcers and other poorly healing wounds. *Int. J. Dermatol.* **2002,** *41*, 453-458.

81. Weber-Dabrowska, B.; Zimecki, M.; Mulczyk, M.; Górski, A. Effect of phage therapy on the turnover and function of peripheral neutrophils. *FEMS Immunol. Med. Microbiol.* **2002,** *34*, 135-138.

82. Weber-Dabrowska, B.; Mulczyk, M.; Górski, A. Bacteriophage therapy for infections in cancer patients. *Clin. Appl. Immunol. Rev.* **2001,** *1*, 131-134.

83. Weber-Dabrowska, B.; Mulczyk, M.; Górski, A. Bacteriophage therapy of bacterial infections: An update of our institute's experience. *Arch. Immunol. Ther. Exp.* **2000,** *48*, 547-551.

84. Weber-Dabrowska, B.; Zimecki, M.; Mulczyk, M. Effective phage therapy is associated with normalization of cytokine production by blood cell cultures. *Arch. Immunol. Ther. Exp.* **2000,** *48*, 31-37.
